# Supporting Children With a Chronic Disease and Their Parents When Admitted to Hospital: A Scoping Review of Psychosocial Supports

**DOI:** 10.1111/apa.70492

**Published:** 2026-03-30

**Authors:** Lyndsay Jerusha MacKay, Nolan Lagrisolal, Una Chang, Matthew Christoffersen, K. Alix Hayden, Kathryn Birnie, Tammie Dewan

**Affiliations:** ^1^ College of Nursing, Texas A & M Bryan Texas USA; ^2^ School of Nursing, Trinity Western University Langley British Columbia Canada; ^3^ Cumming School of Medicine, Department of Pediatrics University of Calgary Calgary Alberta Canada; ^4^ Department of Biological Sciences University of Calgary Calgary Alberta Canada; ^5^ Library and Cultural Resources University of Calgary Calgary Alberta Canada; ^6^ Cumming School of Medicine, Department of Anaesthesiology, Perioperative and Pain Medicine, University of Calgary Calgary Alberta Canada

**Keywords:** caregivers/psychology, chronic disease, family therapy, hospitalized, medical traumatic stress, mental health, paediatric care, parent/psychology, psychosocial interventions, social supports

## Abstract

**Aim:**

The aim of this scoping review was to identify, examine, and summarize available evidence regarding psychosocial supports provided to children with a chronic disease when admitted to hospital and their parents.

**Method:**

The JBI methodology for conducting and reporting scoping reviews was followed. Seven databases were searched, 15 181 titles and abstracts were screened, and data from the included studies were extracted. The psychosocial supports were grouped into the following domains: emotional and psychological, informational, social, spiritual, and practical. Data describing ho the psychosocial support was offered within each domain were thematically analysed.

**Results:**

A total of 59 studies met inclusion criteria. Emotional and psychological interventions were the most frequently utilized. Most interventions were multimodal, delivered in person, and healthcare professionals and researchers served as primary administrators of the interventions. A vast majority of the interventions yielded positive outcomes, and there were no reported harms.

**Conclusion:**

Comprehensive and multimodal psychosocial interventions should be developed and implemented among hospitalized children with a chronic disease and their parents. The predominance of emotional and psychological support interventions reflects the critical need to address the mental health impacts of chronic illness and hospitalization on children with a chronic disease and their families.

## Summary

- Children with chronic disease and their parents experience significant stress, anxiety, and depression when admitted to hospital, yet psychosocial supports are not regularly provided despite the known benefits.
- Findings show psychosocial supports were predominantly multimodal, encompassed emotional, psychological, information, social, spiritual, and practical domains, with positive outcomes.
- Paediatric healthcare organizations should prioritize the development of comprehensive psychosocial support interventions to improve outcomes and reduce long‐term psychological impacts.

## Graphical abstract

Importance of psychosocial support.

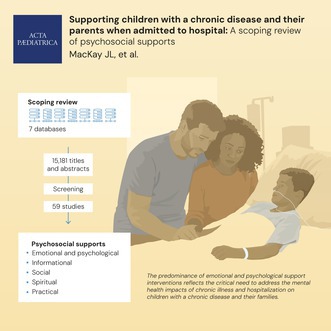

**Abbreviations**
CBT: cognitive behavioural therapy
HCP: healthcare professionals
PMTS: paediatric medical traumatic stress

## 1 Introduction

Admission to hospital can be a time of great uncertainty for children and their parents, with potential for long‐lasting impacts on their mental health and well‐being. Studies have demonstrated that children and their parents experience significant levels of stress, anxiety, depression, and posttraumatic stress [1, 2, 3, 4, 5], as well as risk for Paediatric Medical Traumatic Stress (PMTS) [6]. PMTS is “a set of psychological and physical responses of children and their families to pain, injury, serious illness, medical procedures, and invasive or frightening treatment experiences” [7]. Children are in a time of rapid and complex development; therefore, PMTS can have significant implications for children's social, emotional, and relational development [8]. Also, children and their parents can experience varying degrees of PMTS, which can become disruptive to their ability to function [9]. In their qualitative study describing the experiences of parents of children with medical complexity, Dewan and colleagues [10] found that the complex role of the parent (e.g., providing expert medical care), interactions with healthcare professionals, and system‐level factors could exacerbate parental PMTS. They recommended that enhanced screening and parental mental health services should be provided to better support parents and families. One way to do this is to offer them access to psychosocial support interventions in the hospital.

Psychosocial care is defined as “the culturally sensitive provision of psychological, social, and spiritual care” [11]. Psychosocial care depends upon healthcare professionals' (HCP) quality communication skills, including verbal and non‐verbal, where empathy can be displayed during quality interactions with patients and families [11]. Care domains included in psychosocial care are: physical, informational, emotional, psychological, social, spiritual, and practical [12]. HCP who spend significant amounts of time with children and parents in the hospital setting are well‐positioned to provide psychosocial supports. By providing psychosocial care to children and their parents, HCP can decrease length of stay and help reduce anxiety and stress among children and families when in hospital [13]. This is because higher levels of support can be protective against psychological outcomes, leading to decreased depressive symptoms and less anxiety among parents [1].

Over 1/3rd of hospital admissions are for children with a chronic condition; these children and their families may be in even greater need for psychosocial care [14]. HCP who spend a significant amount of time with children and parents in the hospital setting (e.g., nurses, physicians, and allied health care professionals) do not receive training on how to provide psychosocial supports [15]. Low confidence in and increased barriers to providing psychosocial care have been associated with increased burnout among HCP [15]. Although it is widely supported that psychosocial supports are beneficial in protecting parents from the negative impacts of their children's hospitalization [16, 17], they are not regularly provided to children with a chronic disease and their parents. The objective of this review was to identify, examine, and summarizeliterature regarding psychosocial supports provided to children with a chronic disease admitted to hospital and their parents. These findings will help inform future research on the development, implementation, and evaluation of a psychosocial intervention.

## 2 Methods

This scoping review was conducted in accordance with the JBI methodology for scoping reviews [17, 18] and reported in accordance with the Preferred Reporting Items for Systematic Reviews and Meta‐Analysis extension for Scoping Reviews (PRISMA‐ScR) checklist (see Appendix S1) [18, 19, 20]. It has been registered with the Open Science Framework (https://doi.org/10.17605/OSF.IO/8VNZ3).

### 2.1 Search Strategy

The search strategy, developed by an expert evidence synthesis librarian (AH), focused on five concepts: chronic disease, psychosocial support, children (ages 0–18), hospital setting, and interventions. First, the team identified seed studies that were used to develop an initial search. As well, other published reviews were analysed to inform the search terms for the complex concepts of chronic disease and psychosocial supports [21, 22, 23]. The initial search was developed in Medline All (Ovid) and then translated for other databases. The search included both keywords and subject headings, was conducted May 2024, and had no time limitations. Databases searched included: Medline All (Ovid), Embase (Ovid), APA PsycInfo (Ovid), JBI EBP Database (Ovid), Cochrane Database of Systematic Reviews (Ovid), Cochrane Central Register of Controlled Trials (Ovid), and CINAHL Plus with Full Text (EBSCOHost). The final search strategies are available in Appendix S2.

### 2.2 Study Selection

**FIGURE 1:**
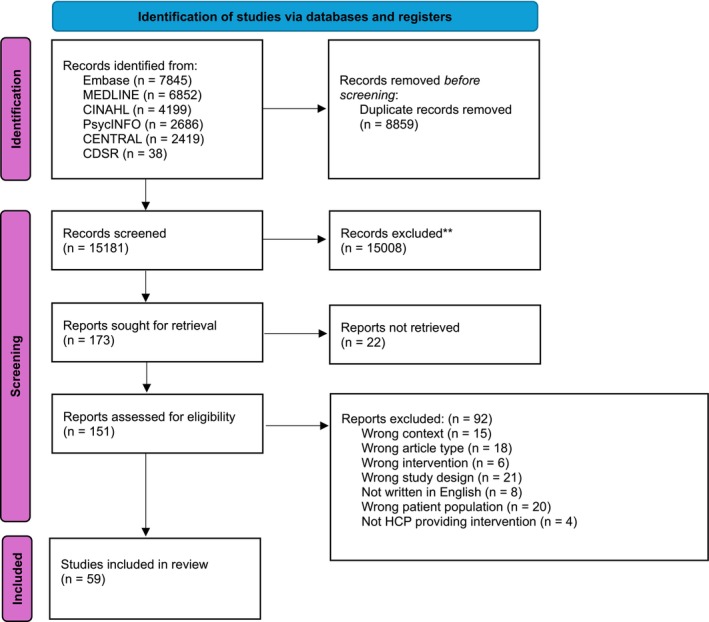
PRISMA flow diagram of source identification for scoping review.

All database records were uploaded into Covidence [24]. All reviewers (N.L., M.C., and L.M.) attained a minimum of 90% test of inter‐rater reliability on 50 titles and abstracts in Excel using the inclusion and exclusion criteria prior to screening in Covidence. See Table 1 for inclusion and exclusion criteria. Two reviewers then screened all titles and abstracts against the inclusion and exclusion criteria in Covidence. Potentially relevant records were retrieved in full text and assessed by two independent reviewers. Reasons for exclusion were documented. Disagreements for both phases were resolved through discussion. Full‐text articles that met all inclusion and exclusion criteria were collated. The results of the search and study inclusion process are presented in Figure 1.

**TABLE 1:** Inclusion and exclusion criteria.

Inclusion	Exclusion
Population	
– Children and infants aged 0–18 who have at least *one chronic disease*. Chronic disease is a medical condition that lasts for longer than 1 year and requires ongoing medical attention. – Sample has a minimum of 50% of children with a chronic disease and 50% of children under the age of 18 – Healthcare professionals and volunteers who deliver the intervention, including: nurses, physicians, psychiatrists, psychologists, social workers, child‐life specialists, occupational therapists, physical therapists, and chaplains – Parents (biological, adoptive, foster parents, legal guardians), caregivers, family members (grandparents, uncles, aunts etc.), siblings *Can be from the perspective of the child, parent, or healthcare professional	– Children with oncological, mental health, or developmental diagnoses without a chronic medical diagnosis – Care coordination and pain management because this literature is extensive and warrants a specific review
Context	
– The intervention took place, or at least a portion of the intervention, within the hospital setting, including inpatient care units. Hospitals include: tertiary paediatric care centers, community hospitals, and regional hospitals – The child must be actively admitted to hospital and spending the night to receive medical care	– Neonatal Intensive Care Unit (NICU) – Doctor office – Mental health care settings – School settings – Public health clinics – Emergency Department – Rehabilitation centre – Hospices and palliative care centre – Any setting outside of a paediatric hospital – Children coming to the hospital for short periods of time to receive medical treatment that does not involve them spending the night
Concept	
– Psychosocial support interventions, defined as, “the culturally sensitive provision of psychological, social, and spiritual care” [25] provided by a healthcare professional in the hospital setting – Care domains of psychosocial care are: physical, informational, emotional, psychological, social, spiritual, and practical [26]. – Psychosocial support interventions can be geared toward the child, family (including parents and siblings), or both – Psychosocial support interventions can be provided by HCP or by peer‐to‐peer support – Psychosocial support interventions can aim to meet the following needs: informational, emotional, psychological, social, spiritual, and practical [26]	– Care coordination – Psychosocial interventions for pain management or procedural pain prevention – Palliative and/or end‐of‐life care approaches
Article type	
– Qualitative research studies – Quantitative research studies – Mixed‐methods research studies – Review (systematic reviews, meta‐analysis and scoping reviews) – Literature reviews for reference reviewing – Case studies	– Grey literature – Popular literature – Letters to the editor – Opinion papers – Studies published in a language other than English – Abstracts only/conference presentations – Dissertations

### 2.3 Data Extraction and Analysis

Data from each study were extracted by one reviewer into a modified version of the JBI data extraction instrument [18] and verified by a second reviewer (N.L., M.C., L.M., and U.C.), which included: author, date, country, purpose/aim, sample, participant characteristics, method, intervention type (including information about the psychosocial care domain [12]), measurement tools, main results, and harms. The data extraction tool was pilot tested on five studies to ensure all study information, data, and results were extracted. Data were descriptively mapped and presented in tables using frequency counts of the following: study method, country, participants, intervention domain type, intervention target, intervention administrator, mode of intervention delivery, length of intervention, child age group, and child diagnosis. There were often multiple approaches within each psychosocial support, which were grouped into the following care domains: physical, informational, emotional, psychological, social, spiritual, and practical [12]. Data describing the approaches to delivering psychosocial support within each domain were thematically analysed to develop themes characterizing the delivery approaches [27].

## 3 Results

### 3.1 Study Characteristics

A total of 15 181 studies were screened and 59 studies that investigated psychosocial supports were included in this scoping review. Two of the studies were qualitative, 42 were quantitative, with randomized controlled trials being the most prominent quantitative method, one case report, six mixed methods, and eight reviews. The studies were conducted in a total of 36 different countries, with authors from the United States, Iran, United Kingdom, and Canada having the most published studies. Publication dates ranged from 1992 to 2024. Intervention types were grouped into five different care domains [12], including: emotional and psychological, informational, social, spiritual, and practical. Most interventions were multimodal in nature, meaning they used a variety of approaches across multiple domains to offer psychosocial support. An in‐depth discussion of each approach used within each of the five care domains is provided below. The majority of interventions were provided in person; however, other modes of delivery included telephone, virtual, computer‐based, and offering a booklet. A summary of the included studies can be found in Table 2. Detailed information on the characteristics of the studies can be found in Table 3, including the target populations and intervention administrators. Due to a large number of diagnoses, children's medical diagnoses were grouped under body systems in Table 3, while specific diagnoses can be found in Table 2.

**TABLE 2:** Summary of Included Studies.

Author, date, country	Purpose, aim	Sample, participant characteristics	Method	Intervention type	Measurement tools	Main results
Aite, Trucchi, Nahom, Zaccara, Casaccia & Bagolan [28], 2003, Italy	To assess the impact on maternal anxiety of a short‐term intervention in a particularly stressful situation, such as a surgical anomaly diagnosed only at birth after repeated negative prenatal ultrasound	Mothers (*N* = 30) of newborns requiring surgical correction of a congenital anomaly Child age group: Term neonatal	Randomized controlled trial	Emotional, Psychological, Informational, and Social: Parent anxiety support intervention Intervention length: Weekly 1‐h meetings Intervention administrator: Psychologist Meetings designed to enable parents to emotionally and cognitively consolidate their experience and give them a chance to express their feelings, facilitate discussion of their concerns and doubts about their baby, and support and reinforce positive parenting behaviour and caregiving skills. Also consisted of weekly team meetings with the paediatric surgeon, psychologist, and head nurse to evaluate the treatment, discuss parental input and priorities, and coordinate team efforts	Spielberger State–Trait Anxiety Inventory (STAI–S)	No significant differences were found at birth in the STAI–S scores of the two groups. At discharge, the intervention group exhibited a much lower STAI–S score than the group without short‐term intervention. The authors concluded that psychological counselling for parents of newborn babies has been shown to be helpful
Akyurek, Gurlek, Ozturk & Bumin [29], 2023, Turkey	To examine the effectiveness of the parent‐based occupational therapy intervention programme on coping skills and stress levels in parents of children with cerebral palsy	Children aged 3–12 (*N* = 21) with cerebral palsy and their parents. Admitted to the paediatric rehabilitation unit, Child age group: early and middle childhood, early adolescence	Randomized controlled trial	Physical, Informational, and Emotional: Parent‐based occupational therapy intervention Intervention length: 10 sessions (twice a week for 5 weeks), 45 min per session Intervention administrator: Occupational therapist The control group received standard occupational therapy, while the study group received parent‐based occupational therapy. While the therapist, parent, and child were in the therapy area in the first session, the child received the same standard occupational intervention as the control group, and during this process, the parents recorded the therapy with a video recorder. In the second 20 min, three activities (such as eating, dressing, or mobility activities), were performed by the child and helped by the parent and video‐recorded by the occupational therapist. Parents were provided with the video and instructed to watch the videos at home. At the next session, the parent and therapist discussed the videos of the previous session, including the parents' feelings and what they learned. At the end of the session, home programme activities were given	Coping Attitudes Assessment Scale Emotional Skills and Competence Questionnaire (ESCQ) Questionnaire on Resources and Stress Freidrich short form (QRS‐F)	The study group showed a statistically significant decrease in stress levels and increases in coping skills, emotional skills and competencies
Asnani, Francis, Knight‐Madden, Chang‐Lopez, King & Walker [30], 2021, Jamaica	To assess the feasibility of a problem‐solving skills training intervention in improving psychological outcomes in mothers of infants with sickle cell disease (SCD)	Mothers (*N* = 64) of children aged 6–12 months with severe SCD genotypes (Hb SS disease and Hb Sβ0 thalassaemia) attending the clinic at the Sickle Cell Unit. Child age group: Infancy	Randomized controlled trial	Social, Practical, and Informational: Problem‐solving therapy Intervention length: In groups, 5 sessions conducted over 2 weeks and totalling 9 h Intervention administrator: SCD clinic nurses Aimed to empower patients or caregivers in attending to daily social and other challenges that might arise, especially with the presence of a chronic illness. The approach is based on cognitive behavioural therapy and has been shown to be of use in primary care settings. This intervention was adapted from the ‘Bright IDEAS’ problem‐solving skills training intervention. Stages of training include: identification of the problem(s), generating possible solutions, evaluating the options, implementing the preferred solution, and evaluating to see if the solutions were successful. The interventions were done in groups. The mothers also received an intervention to build their skills in promoting their child's development	Paediatric Inventory for Parents (PIP) Coping Health Inventory for Parents (CHIP) Social Problem Solving Inventory‐Revised: Short Form (SPSI‐R:S) Centre for Epidemiologic Studies Depression Scale (CESD)	The intervention resulted in no change to the mothers' problem solving skills, coping behaviours, or depressive symptoms. There was a significant benefit of reducing mothers' difficulty managing parental stress. Depression scores decreased significantly more in mothers of the intervention group compared to the control group
Badr, Ibrahim & Saleh [31], 2023, Egypt	To investigate the effect of Benson's relaxation technique versus music intervention on physiological parameters and stress of children with thalassaemia during blood transfusions compared to a control group who received standard care	Children aged 3–6 years (*N* = 120) with thalassaemia undergoing a scheduled blood transfusion Child age group: Early and middle childhood	Randomized controlled trial	Psychological and Physical: Benson's relaxation group Intervention length: Single sessions prior to/during blood transfusion Intervention administrator: Researcher The researcher educated each child individually on how to do Benson's relaxation technique in a quiet, separate room before and during the blood transfusions. First, the child sat calmly in a comfortable position and was instructed to close his eyes. Second, progressively relax all muscles deeply, beginning at the feet and progressing up to the face. Finally, the child was instructed to breathe easily and naturally through his nose and be aware of breathing patterns, and during breaths out, say the word “one” silently Psychological and Physical: Music intervention Every child listened to recorded classic relaxation Mozart's music in a quiet separate room before and throughout the blood transfusions procedure by using an MP3 player	Physiological parameters (respiratory rate, heart rate, and oxygen saturation) Observational Scale of Behavioural Distress (OSBD)	Participants in the music and Benson group had significantly lower physiological parameters and behavioural stress responses during and after the procedure compared to the control group
Bahrami, Pahlavanzade & Marofi [32], 2019, Iran	To design a program based on the adaptive behaviours presented by Tak and McCubbin and determine the effect of this program on the children's anxiety and their mothers' caregiver burden.	Children ages 6–12 (*N* = 122) with a diagnosis of nephrological disorders including: chronic kidney diseases, end‐stage of renal disease, nephrotic syndrome, and urinary tract infection. The mothers of children were included. Child age group: Middle childhood, early adolescence	Randomized controlled trial	Informational and Psychological: Supportive training program Intervention length: 5 sessions, 45–50 min each Intervention administrator: Unspecified/Various The supportive training program was a self‐regulated program. The contents were developed based on the needs of the subjects, review of scientific articles, and opinion of psychiatry professors, psychologists, and psychiatric groups. A group comprised six to eight children and mothers and the program was held for children and their mothers at same time but separately. At the end of each session, the materials covered on the same day were printed and given to the mothers	Faces Anxiety Scale (FAS) Zarit Caregiver Burden Scale (ZBS)	After the intervention and 1 month after the intervention, the mean children's anxiety and their mothers' caregiver burden score of the control group was significantly higher than the experimental group. The results showed that the supportive training program can reduce children's anxiety and their mothers' caregiver burden
Bee, Pedley, Rithalia, Richardson, Pryjmachuk, Kirk & Bower [33], 2018, United Kingdom, United States, Canada	To determine which models of self‐care support for long‐term conditions (LTCs) are associated with significant reductions in health utilization and costs without compromising outcomes for children and young people	Children and young people aged 0–18 years with a long‐term physical or mental health condition (e.g., asthma, depression) 127 papers reporting on 97 studies were included Child age group: Term neonatal, infancy, toddler, early and middle childhood, early adolescence	Systematic review with meta‐analysis of 77 articles	Social, Physical, Psychological, and Informational: Self‐care support delivered in a health, social care, or educational setting Intervention length: Unspecified/Various Intervention administrator: Unspecified/Various Self‐care support intervention was defined as: any intervention primarily designed to develop the abilities of children and young people (and/or their adult carers) to undertake management of their long‐term health condition through education, training, and support to develop their knowledge, skills, or psychological and social resources	Reported quantitative data on patient outcomes and health‐care utilization.	Meta‐analysis of all study data demonstrated that self‐care support was associated with statistically significant but minimal improvements in QoL. Self‐care support was associated with minimal but statistically significant reductions in ED use.
Biabani, Kermansaravi & Navidian [34], 2020, Iran	To explore the effect of group training on adaptive behaviours and caregiving burden of mothers of children with thalassaemia major	Mothers (*N* = 70) of children aged 1–5 years with thalassaemia major. Child age group: Toddler, early childhood	Randomized controlled trial	Informational, Psychological, and Spiritual: Group‐based training program Intervention length: 4 sessions (one 120‐min session per week) in groups of 8–9 people. Intervention administrator: Researcher and psychiatric nurse During the sessions, mothers were encouraged to ask their questions and engage in group discussions. Session 1: disease overview, complications, diet, activity, relaxation. Session 2: review of session 1 with a discussion of challenges and problem‐solving. Session 3: review of previous sessions and discussion about stress, anxiety, and anger management. Session 4: a review of previous sessions with a discussion about acceptance, social support, and spiritual considerations. At the end of each session, the researcher and the psychiatric nurse summarized the materials. The subjects were asked to practice the presented skills at home; they were also provided with the researcher's phone number for counselling and solving their possible questions	Coping Health Inventory for Parents (CHIP) Caregiver Burden Scale (CBS)	Group training with mothers resulted in a significant decrease in caregiver burden and an increase in adaptive behaviours
Blue, Kasparian, Sholler, Kirk & Winlaw [35], 2015, Australia	To assess the efficacy of individualized genetic counselling sessions in improve knowledge of causation and psychosocial functioning in parents of children with congenital heart disease (CHD)	Parents (*N* = 57) attending preadmission clinic prior to their child's elective cardiac surgery Child age group: Unspecified/Various	Pre‐test post‐test quantitative study	Informational and Emotional: 1‐h genetic counselling session Intervention length: 1 session (1‐h) Intervention administrator: Genetic counsellor The format was semi‐structured, covering a set of key issues and tailoring information such as recurrence risk estimates and emotional support to individual participants' needs. Key areas covered in the session included an overview of CHD incidence, detailed family history, and other topics	Sources of Genetics Information Measure Emotional Aspects of Having a Child with CHD Measure Knowledge Measure Perceived Personal Control (PPC) Personal Feelings Questionnaire (PFQ‐2) Depression, Anxiety and Stress Scale (DASS‐21) Genetic Counselling Satisfaction Scale (GCSS) Perceptions of the Genetic Counselling Session	Knowledge scores improved significantly, and parents retained information over time. Perceived personal control increased with decreases in guilt, shame, depression, anxiety, and stress. Satisfaction with the intervention was high
Bradshaw, Bem, Shaw, Taylor, Chiswell, Salama Bassett, Kaur & Cummins [36], 2019, United States, United Kingdom, Australia, Canada, Germany, Switzerland, Iceland, Sweden, Chile, China, Denmark, Iran, Japan, Malaysia, Mexico, New Zealand, Spain, Thailand	Synthesize interventions aimed at improving the health, well‐being, and parenting skills for children with special health care needs (CSHCN) and medical complexity	Parents of CSHCN who underwent interventions aimed at improving their well‐being and parenting skills Child age group: Unspecified/Various	Scoping review of 65 studies	Social, Information, Practical, Psychological, and Emotional Five main categories: parenting programs, parent behaviour change interventions, preparation and support for hospitalization, and other Length of intervention: Unspecified/Various Intervention administrator: Unspecified/Various Parenting programs: Based on the premise that such programs can reduce child behavioural, emotional or psychological problems, and improve child and parent outcomes Peer support programs: The primary mechanism of action was the development of peer support, including online peer support groups, an email list service, telephone peer support groups, and one to one peer matching Preparation and support for hospital admission or discharge: To provide support to parents around their child's hospital admission, including pre‐admission preparation, information on admission, and post‐discharge support Targeted parent behaviour change interventions: Aimed to change aspects of parenting behaviour including: Acceptance and Commitment Therapy (ACT), Cognitive Behavioural Therapy (CBT), Problem Solving Skills Therapy (PSST), coping skills training, multi‐family therapy, individual family therapy, adapted Chronic Disease Self‐Management based programs, group meetings, residential programs, an interactive online application, participatory training, and filial therapy, based on play therapy Others: child massage training, mindfulness/relaxation, narrative therapy, therapeutic conversations, wish granting, communication skills training, and financial counselling	Intervention descriptions were assessed according to the Template for Intervention Description and Replication (TiDieR) framework	Half of the studies reported findings that were fully in favour of the intervention, (*n* = 31, 52%) with the rest producing mixed results (*n* = 25, 42%). Although not routinely collected across the studies, there were no reports of serious harm to participants. Interventions that took a behavioural or cognitive‐based approach tended to report more favourable outcomes than those taking a family and ecological systems‐based approach. Studies that took a mixed approach encompassing behavioural cognitive and family and ecological systems theories reported fully positive findings
Brooks & Palau [37], 2023, United States	To utilize evidence from the literature and available resources to improve the self‐efficacy of caregivers of children with seizures at a large paediatric medical center in the southern United States.	Caregivers (*N* = 31) who were to be sent home with a seizure rescue medication for their children who were either recently diagnosed with seizures or who had incurred a change to their seizure treatment plan were included Child age group: Unspecified/Various	Quantitative Evidence based practice study	Informational, Physical, and Practical: Parent simulation education Intervention length: 1 session (45 min‐1.5 h) Intervention administrator: Clinical nurse leader Parent simulation education sessions took place by a clinical nurse leader in a private treatment room on the unit, but away from the child's bedside, and lasted approximately 45 min to 1.5 h. Each session was adapted to align with the individual child's seizure treatment plan and discharge instructions. Parents touched and handled their home rescue medication, and parents responded to a seizure in the human‐patient simulator and correctly administered the rescue medication. After the simulation, parents were offered guidance on improvements and praise for correct responses. Parents were also offered a time to ask questions	KidSIM‐ASPIRE Parent Seizure Self‐Efficacy Questionnaire	A statistical comparison of the pre‐ and post‐KidSIM‐ASPIRE Parent Seizure Self‐efficacy Questionnaire responses suggested participation in the simulation training increased caregiver self‐efficacy to respond to a seizure in the home environment
Brown, Krieg & Belluck [38], 1995, United States	To describe a psychosocial group intervention program developed at a Cystic Fibrosis Center in a children's hospital to maximize adaptive and functional responses. Also, to describe the issues and themes which emerge for patients, families, and staff as current treatment brings chronically ill children into adolescence and young adulthood	Parents who have children experiencing a cystic fibrosis (CF) Child age group: Term neonatal, infancy, toddler, early and middle childhood, early and late adolescence	Descriptive study/framework discussion	Informational, Social, and Emotional: Group intervention program Intervention length: Parent groups of children under 4 years old meet for 1 year semi‐monthly. Parent groups of children over 18 meets semi‐monthly for 4–6 sessions Intervention administrator: Social workers Focuses on information‐giving and education, socialization, sharing and emotional support, and behavioural changes necessitated by the patient's condition or care needs	Interviews	The following five themes have emerged from both groups: the value of supportive networking, patient care and disease management, grief reactions, impact of CF on the family, role of the CF Center and Medical Center in their lives
Burke, Harrison, Kauffmann & Wong [39], 2001, Canada	To test the effectiveness of a family‐focused, supportive intervention, Stress‐Point Intervention by Nurses (SPIN), designed to reduce family problems	Nurses (*N* = 23) and children (*N* = 115) with an average age of 7. Primary medical diagnoses were cerebral palsy (*n* = 23), spina bifida (*n* = 16); congenital genitourinary defects (*n* = 15); cancer, responding to treatment (*n* = 14); chronic renal disease (*n* = 12); cystic fibrosis (*n* = 6); congenital hip defects (*n* = 4); other orthopaedic conditions (*n* = 8); cardiac defects (*n* = 3); gastrointestinal conditions (*n* = 3); muscular dystrophy (*n* = 3); cleft palate (*n* = 2); diabetes (*n* = 2); epilepsy (*n* = 2); and other (*n* = 2). Child age group: Toddler, early and middle childhood, early adolescence	Clinical trial	Psychological: Stress‐point intervention by nurses (SPIN) Intervention length: Occurred 2 weeks before hospitalization, included hospitalization, and concluded about 2 weeks after discharge. Nurse contact time per family ranged from a few minutes to 8.5 h, with an average of 3.3 h per family. Intervention administrator: Nurses SPIN is a psychosocial and educational intervention that focuses on parents' issues and concerns surrounding their child's hospitalizations. It involves: (a) identifying the family's own particular stressful issues surrounding the expected or anticipated hospitalization, (b) developing a plan with the parent to handle their specific issues, and (c) following up to praise strengths and successes, modify, and evaluate the success of the intervention	Feetham Family Functioning Survey (FFFS) Coping Health Inventory for Parents (CHIP) Nurse Contact Activity and Intervention Record Burke Stressor Checklist	Parents who engaged in SPIN were more satisfied with family functioning and had better coping after hospitalization than parents who received usual care. Increased nurse time had a significant positive effect on overall coping scores. Parents in the SPIN group had increased family coping by maintaining family integration, cooperation, and an optimistic definition of the situation and health care communication coping by understanding the medical situation through communicating and consultation
DeMaso, Gonzalez‐Heydrich, Erickson, Grimes & Strohecker [40], 2000, United States	To test the feasibility and safety of a computer‐based application designed to facilitate the healthy coping of children and their families who must contend with significant congenital heart disease (CHD)	Mothers (*N* = 9 in phase 1, *N* = 40 in phase 2) who had children aged 6 weeks to 25 years hospitalized for cardiac disease at a paediatric medical centre Child age group: Term neonatal, infancy, toddler, early and middle childhood, early and late adolescence	Mixed methods	Psychological, Emotional, and Social: Experience journal (EJ) Intervention length: Unspecified/Various Intervention administrator: Computer‐based application The EJ is a psychoeducational intervention based on a narrative model involving the sharing of personal stories about an illness. The EJ works by accepting descriptions from families about what it has been like to live and cope with a medical condition or illness (ranging from short written explanations of an experience to pictures, poems, stories, and videos). Child life specialists, psychiatry, and social workers collected contributions from patients and families	Semi‐structured interviews to elicit both quantitative ratings and qualitative data. Parents provided ratings on 7 categories along with descriptions of each during interviews for phase 1 and phase 2	The mean overall satisfaction (mean = 5.7) was quite high, as was the satisfaction (mean = 6.0) with the presentation of the stories in the EJ. Mothers also reported that the EJ assisted in decreasing social isolation. Mothers reported moderate increases in their understanding of their own, their whole family's, their spouse's, and their children's feelings about their child's heart disease that they attributed to using the EJ
Dews, Pokowitz, Votta, Yan, Pituch & Deldin [41], 2023, United States	To pilot test the adapted program of Mood Lifters, assessing for feasibility and acceptability, and evaluate the preliminary impact of the program on the mental health of parents of medically complex children	Adult parents (*N* = 18) of chronically ill or medically complex children Child age group: Unspecified/Various	Quantitative, Quasi‐experimental	Psychological, Emotional, and Social: Mood lifters: a peer‐led wellness program to equip parents of medically complex children with evidence‐based strategies to manage their mental health while also reducing barriers to support Intervention length: 15 weekly 1‐h meetings Intervention administrator: Peer leader who had previously participated in the program and obtained certification by completing a training course. Participants learn evidence‐based strategies to improve stress, depression, and anxiety in a group setting. It follows a biopsychosocial approach to mental health, combining skills from CBT, dialectical behaviour therapy, and acceptance and commitment therapy. Each meeting centered on 1 of 6 biopsychosocial domains (behaviour, mind, mood, body, sleep, and social). Following each meeting, participants were encouraged to earn points by practicing the skills introduced throughout the program. Participants logged their points on an accompanying app between meetings. At the following meeting, participants had the opportunity to discuss their practice from the previous week	Patient health questionnaire (PHQ‐9) Generalized Anxiety Disorder‐7 (GAD‐7) Perceived Stress Scale (PSS) Scale of Positive and Negative Experience (SPANE) Flourishing Scale (FLO) Social Functioning Questionnaire (SFQ)	Data from participants showed improvements in depression, anxiety, perceived stress, and positive and negative emotions
Dinc, Yildiz & Ercan [42], 2024, Turkey	Evaluate the effectiveness of an education program based on healthcare transition provided to adolescents diagnosed with asthma	Adolescents (*N* = 52) ages 14–18 with mid‐to‐moderate severity of asthma Child age group: Early adolescence	Randomized controlled trial	Informational: Education program Intervention length: 10 sessions (within 3 months) (30–40 min per session) Intervention administrator: Researcher Content included asthma symptoms and triggers, attacks, action plans, asthma management, asthma camps, associations, and situations that increase treatment compliance, and the differences between paediatric and adult asthma care, and communication with asthma team.	Transition Readiness Assessment Questionnaire (TRAQ) Self‐Efficacy Scale for Children and Adolescents with Asthma (SESCAA) Mind the Gap Scale (MGS)	Children who received the intervention had significantly increased transition readiness assessment questionnaire and self‐efficacy scores than the control group, while the mind the gap scores were significantly lower
[43] Donegan, Boyle, Crandall, Dotson, Lemont, Moon & Kim, 2016, United States	To describe the feasibility of the development and implementation of a volunteer, parent mentoring program that went from an inflammatory bowel disease (IBD)‐patient focused program to one that rapidly expanded to a hospital‐wide program involving more than 200 mentors matched to over 300 mentees within a 2‐year period.	Families (*N* = 378) of children with IBD Child age group: Unspecified/Various	Case report	Informational, Social, and Emotional: Parent mentor program Intervention length: 3 phone calls between parent mentor and mentee Intervention administrator: Parent mentor (veteran parent whose child has been diagnosed with an illness for longer than 1 to 2 years) Parent mentoring programs connect an experienced parent who underwent parent mentor training with a parent of a newly diagnosed child with a similar diagnosis. Parent mentoring programs offer a unique one‐to‐one relationship. Typically, the mentor has completed a comprehensive training program. The focus of parent support includes affirmational support, informational support, and emotional support. When the child is hospitalized, parent mentoring is also offered	N/A	The parent mentor program demonstrated feasibility in the development and implementation within a relatively short time frame and with limited additional resources Benefits: parents feel more comfortable expressing concerns to mentor vs. GI team. Mentors offered practice tips and helped parents navigate websites. The mentor experienced personal gratification for helping others Challenges: it was difficult for mentors to provide support when in hospital, and the IBD team did not receive funding or release time
Duffy & Vessey [44], 2016, United States	To test the efficacy of the Creating Opportunities for Parent Empowerment (COPE) intervention for parents of children with neurological conditions	Parents of children aged 2–6 (*N* = 46) with a chronic neurological condition Child age group: Toddler, early and middle childhood	Randomized controlled trial	Informational, Emotional, Psychological: Creating Opportunities for Parent Empowerment (COPE) Intervention length: Unspecified/Various Intervention administrator: Researcher COPE teaches parents how their child might react to being hospitalized and then instructs parents on how to best respond to their child's needs	Parental Beliefs Scale Beck Depression Inventory II (BDI‐II) State–trait Anxiety Inventory (STAI) Behaviour Assessment System for Children‐Parent Report Scale	Parental belief scores increased among the intervention group, demonstrating COPE had an impact on parents' confidence. There was no change in depression or anxiety. However, parents' anxiety and children's internalizing behaviours are highest when in hospital. The COPE intervention may not be successful among children with chronic neurologic conditions
Fung, Ho, Fung, Leung, Chow, Ha & Barlaan [45], 2011, Hong Kong	To examine the efficacy of a strength‐based mutual support group for reducing stress and enhancing psychological well‐being of caretakers of children with cerebral palsy	Primary caretakers (*N* = 12) of cerebral palsy patients aged 8 to 19 years attending follow‐up orthopaedic and traumatology clinic. Child age group: Middle childhood, early and late adolescence	Pre‐ and post‐ intervention	Informational, Psychological, and Social: The strength‐focused support group for caretakers Intervention length: 4 weekly sessions (90 min each) Intervention administrator: Cinical psychologists The objective of this intervention was to help caretakers to identify positive resources and key strengths of their child, as well as to coach them to develop their child's identified key strengths. Caretakers were also guided to identify their own character strengths and enhance their positive emotions, for which handouts, experiential exercises, videos and questionnaires were used. Parents were coached on how to identify current parent problems and set concrete plans about how to make use of the child's strengths to resolve these problems	Appraisal of Severity of Disability Parenting stress (PSI‐SF) Multidimensional Scale of Perceived Social Support (MSPSS) The Social Avoidance and Distress Scale The Hospital Anxiety and Depression Scale (HADS) The Satisfaction with Life Scale (CSWLS26) The Subjective Happiness Scale The Rosenberg Self‐Esteem Scale (RSES) The Hope Scale	Half of the caretakers attended the full intervention program. Parenting stress, depression, social support, and hope improved significantly. Perceived social support was significantly increased during the intervention but not after it ended
Gholami, Reyhani, Toosi & Vashani [46], 2016, Iran	To investigate the effect of a Supportive Educational Program on self‐efficacy of mothers with epileptic children	Mothers of children with epilepsy (*N* = 100) admitted to hospital. Children aged 1–12 years old, at least 6 months diagnosed with the disease Child age group: Toddler, early and middle childhood, early adolescence	Randomized controlled trial	Informational: Supportive educational program Intervention length: 5 sessions (60‐min each) Intervention administrator: Researcher, group discussion (7–8 participants) It consists of three phases: phase one (threat perception: causes of epilepsy relapse and complications), phase two (problem‐solving: empowered on how to implement emergency measures), and phase three (evaluation).	Steffen's Revised Scale for Caregiving Self‐Efficacy	Self‐efficacy was significantly higher in the study group compared to the control group. The Supportive Educational Program contributed to an increase in maternal awareness about how to provide care, reasons for recurrence, and emergency care
[47] Gordon, Gordon & Basu, 2023, United Kingdom, United States, France	This review aimed to explore the existing literature on use of social prescribing (SP) for children and young people (CYP) with neuro‐disability within a hospital setting	Included studies (*N* = 8) of SP as an intervention for CYP (aged 0–22) Child age group: Infancy, toddler, early and middle childhood, early and late adolescence	Systematic review	Social, Practical, Emotional, and Psychological: Social prescribing (SP) Intervention length: Unspecified/Various Intervention administrator: Unspecified/Various Non‐medical intervention which involves professionals referring patients to a link worker, who connects them with appropriate (e.g., financial, social, mental well‐being, or practical) support. To achieve this, the link worker takes time to understand their client's agenda (often expressed in lay terms as ‘what matters to me?’) and develops a holistic, personalized plan to address their needs	Study characteristics and findings including both qualitative and quantitative outcomes were presented in tables and summarized narratively	Outcomes from the interventional studies demonstrated significant increases in children's health and well‐being, feelings of support, information received, and satisfaction with the interventions
Grootenhuis, Maurice‐Stam, Derkx & Last [48], 2009, Netherlands	To evaluate whether a psychoeducational group intervention (aiming to enhance information seeking and giving about the disease, relaxation, social competence, and positive thinking) can strengthen the coping efforts of adolescents with inflammatory bowel disease (IBD) and have a positive effect on their Health‐Related Quality of Life (HRQoL)	Children aged 12–18 years (*N* = 40) with IBD. Child age group: Early adolescence	Randomized controlled trial	Informational, Psychological, Social, and Emotional: Op Koers (OK) program in 6 sessions. Intervention length: 6 sessions Intervention administrator: Psychologists This program uses a cognitive behaviour therapeutic approach, such as modelling, contingency management, exposure exercises, and cognitive techniques. This program consists of information giving and seeking, storytelling, role playing, social competence, and relaxation exercises. Feelings of being different because of their disease is discussed. Positive thinking is encouraged by identifying and correcting inaccurate thoughts.	The Cognitive Control Strategies Scale for Paediatric Patients (CCSS) The Self‐Perception Profile for Adolescents (SPPA) Dutch version of the State–Trait Inventory for Children (ZBV‐K) Dutch Children's AZL/TNO Quality of Life Questionnaire (DUX‐25)	Children who received the intervention had increased use of predictive control strategies and significantly more positive expectations (coping), self‐esteem, self‐worth, physical appearance, and health‐related quality of life
Hampel, Rudolph, Stachow & Petermann [49], 2003, Germany	To investigate the efficacy of a multimodal education program for children and adolescents with asthma	Children aged 8–16 years (*N* = 49) children with asthma Child age group: Middle childhood, early adolesence	Randomized controlled trial	Psychological, Emotional, and Informational: Multi‐modal patient education program that uses cognitive restructuring and acquisition of emotion‐focused coping skills Intervention length: 10 sessions (1‐h each) in groups Intervention administrator: Psychological, educational, and medical staff Sessions provided information about the disease, treatments, triggers, self‐care techniques, anti‐stress training, and cognitive behavioural stress management training. This programed aimed to improve regiment adherence, identify trigger factors for exacerbation of asthma, and develop and employ effective strategies to cope with daily and asthma‐specific stressors	Disease‐related Measures German Coping Questionnaire for Children and Adolescents (SVF‐KJ)	The experimental treatment elicited significant improvements in adaptive coping in adolescents aged from 14 to 16 years. In contrast, substantial effects were not yielded for the control treatment. The results suggest that multimodal patient education training has beneficial effects on stress management in adolescents with asthma
Hashemi, Darshori, Sharif, Karimi & Zare [50], 2015, Iran	To determine the effect of teaching coping strategies to adolescents with thalassemic disease	Adolescents (*N* = 87) aged 11–18 with major Thalassaemia Child age group: Middle childhood, early adolescence	Randomized controlled trial	Psychological, Informational, and Emotional: Training sessions Intervention length: 7 sessions (1.5 h) every 2 weeks Intervention administrator: Researcher and clinical psychologist Sessions included information about thalassaemia, its treatment and complications, daily activities, physical activities, negative emotions and their control, reasonable thinking, stress and its side effects, and managing stress and efficient coping strategies. The training content was presented in the form of lecture, questions, and answers	Jalowice's Coping Strategies Questionnaire	The mean scores of problem‐focused and emotion‐focused coping strategies significantly increased in the experimental group at 1 and 2 months after the intervention. Teaching coping strategies improved the use of problem‐focused and emotion‐focused coping strategies in adolescents with thalassaemia major, specifically coping with stress and the disease
He, Zhu, Chan, Klainin‐Yobas, & Wang [51], 2015, United States, Hong Kong, Australia	To examine the effectiveness of therapeutic play intervention in reducing children's perioperative anxiety, negative behaviours, and postoperative pain, as well as their parents' perioperative anxiety	Six studies with children (*N* = 625) aged 2–12 years old and their parents (*N* = 140) who underwent various types of surgeries Child age group: Toddler, early and middle childhood, early adolescence	Systematic Review	Psychological, Emotional, and Informational: Therapeutic play Intervention length: Unspecified/Various Intervention administrator: Unspecified/Various Any intervention using therapeutic play, which refers to the use of play intervention, videos, and dolls to help children regain control, express feelings of anxiety, gain information about hospital procedures, prepare for medical events, and transform children from passive sufferers to active agents of their perioperative care in the hospital	Narrative summaries were created from the studies.	There were conflicting outcomes regarding effectiveness of therapeutic play interventions in children's anxiety, negative behaviours, and postoperative pain. Two studies showed that the intervention significantly reduced parents' preoperative anxiety. Another study demonstrated children became more calm with play but were no more cooperative during the medical procedure. Evidence on effectiveness of therapeutic play interventions on anxiety, negative behaviours, and pain is inconclusive
Hoare & Kerley [52], 1992, United Kingdom	To evaluate the effectiveness of a parents' group counselling program to reduce the psychosocial morbidity of children with epilepsy and their families and to identify the factors associated with successful or unsuccessful outcome	Parents (*N* = 108) of children aged 5–15 years with epilepsy Child age group: early and middle childhood, early adolescence	Mixed methods	Informational, Emotional, and Social: Group counselling meetings Intervention length: Unspecified/Various Intervention administrator: Unspecified/Various Series of group meetings with other parents and healthcare providers to discuss various psychosocial problems connected with epilepsy for themselves and their children. Topics of discussion included medial aspects of treatment, educational problems and effects on family life. Precise content of the meetings was determined at the first meeting. Meeting choices were flexible for parents to maximize compliance and involve fathers	Rutter Parent and Teacher Questionnaires (GHQ‐30) Golombok‐Rush Inventory of Marital Satisfaction (GRIMS) Holroyd Questionnaire on Resources and Stress Edinburgh Parental Attitude Scale to Epilepsy (EPASE) Questionnaire	This intervention project was remarkably unsuccessful in demonstrating the value of parents' groups for reducing psychosocial morbidity among children with epilepsy and their families. This study also showed that parents prefer individual counselling to the group approach and that they would like the opportunity to see a trained counsellor attached to the clinic
Hughes, Shelton, Penny & Thompson [53], 2023, United States, Canada, France, Iran	To conduct a synthesis of findings related to the potential role of mindfulness‐based interventions for promoting psychological well‐being in children, adolescents, and families affected by childhood illnesses	Eighteen studies with children and adolescents (8–18 years old) and their parents or caregivers with conditions such as chronic pain/diseases, cancer, heart conditions, headaches, oesophageal atresia irritable bowel disease, and polycystic ovary syndrome Child age group: Middle childhood, early adolescence	Systematic review	Psychological: Mindfulness‐based interventions (MBIs) Intervention length: Unspecified/Various Intervention administrator: Unspecified/Various Mindfulness‐based stress reduction aimed at reducing behaviours associated with stress, and mindfulness based cognitive therapy incorporating mindfulness exercise with cognitive behavioural therapy techniques. Mindfulness‐based interventions are based on the central concept described by Jon Kabat‐Zinn (1994, 4) as developing a sense of “awareness that arises through paying attention on purpose, in the present moment, non‐judgmentally”	Findings were presented as a summary of the interventions themselves, parent involvement, children's anxiety and depression, caregiver stress and anxiety, and feasibility and acceptability	Ten studies reported significant improvements in measures of anxiety, and six reported significant improvements in depression following participation in MBIs. Parents reported the value of concurrent caregiver sessions and felt that incorporating mindfulness into their own lives and connecting with other parents helped to reduce stress. MBIs were feasible and acceptable and show promise in improving anxiety and depression of children, but limited support for reducing stress in the family unit
Jahri Sheijani, Chehrzad, Reza Masouleh, Nezhad Leyli & Bidabadi [54], 2020, Iran	To investigate the effect of Creating Opportunities for Parent Empowerment (COPE) program on parents of children with epilepsy or other chronic neurological conditions	Mothers (*N* = 88) of 3–12 year old children with epilepsy or other chronic neurological disorders associated with epileptic seizure. Child age group: early and middle childhood, early adolescence	Quasi‐experimental trial	Informational: COPE Intervention length: Unspecified/Various Intervention administrator: Unspecified/Various The program focuses on increasing parents' knowledge and understanding of child behavioural changes during hospitalization and after discharge from the hospital, as well as parents' direct involvement in the physical and emotional care of their children	Spielberger State–Trait Anxiety Inventory (STAI)	Mothers had the highest level of anxiety during hospitalization with their child. Mothers in the intervention group experienced significantly lower levels of anxiety 1 week and 8 weeks after discharge compared to the usual care group
Karbandi, Far, Salari, Asgharinekah & Izie [55], 2020, Iran	To determine the effect of music therapy and distraction cards on anxiety in hospitalized children with chronic diseases to reduce the destructive effects of anxiety on children after hospitalization	Children (*N* = 83) with chronic diseases aged 8–12 years who were hospitalized Child age group: Middle childhood and early adolescence	Randomized controlled trial	Psychological and Physical: Music therapy and distraction cards Intervention length: 2 sessions (20 min each) Intervention administrator: Researcher In the music therapy group, the children listened to some music of their interest through headphones In the distraction cards group, five‐eight‐cm cards were used, each containing a different image and shape. Firstly, children carefully looked at the cards and asked the researcher about them, followed by interactive playing for a minimum of 20 min	Spielberger State Trait Anxiety Inventory (STAI) Spence Children's Anxiety Scale (SCAS)	Children in the cards and cards with music groups had significantly decreased anxiety compared to the music only group. Playing with distraction cards decreased anxiety and fear in children to a greater extent compared to the music therapy only group
Kavitha, Padmaja & Basheerahamed [56], 2024, India	To evaluate the impact of the Supportive and Coping strategies, Ongoing Assessment, Prevention of Complications, and Empowerment (SCOPE) Program on the health and health‐related quality of life (HRQoL)of children with thalassaemia	Children (*N* = 80) with beta‐thalassaemia major age 6 to 18 years Child age group: Middle childhood, early adolescence	A quasi‐experimental pretest–posttest control group with a sequential follow‐up design	Social, Emotional, and Informational: Supportive and Coping strategies, Ongoing Assessment, Prevention of Complications, and Empowerment (SCOPE) program Intervention length: Once a month assessment for 6 months and weekly phone calls Intervention administrator: Unspecified/Various This program provides social support in the form of a parent support group, reinforcement of positive coping skills, advocacy for free healthcare services, on‐going assessment of condition, prevention of complications via education about maintain hygiene, vaccination, prevention of infections, nutritional counselling, and empowerment via individualized, family‐centered education, and weekly phone calls	Paediatric Quality of Life Inventory (PedsQL)	The children's self‐reported HRQoL scores during the pretest were poor in all domains in both the intervention and control groups. However, the mean HRQoL scores of the children in the intervention group improved significantly during the posttest and subsequent follow‐up evaluation. There was a statistically significant difference in all four dimensions of HRQoL, namely physical functioning, emotional functioning, social functioning, and school functioning. It suggests that the SCOPE Program intervention is effective in improving the HRQoL for children with thalassaemia
Khanjari, Jahanian & Haghani [57], 2018, Iran	To investigate the effect of blended training on quality of life in children with nephrotic syndrome	Children (*N* = 76) ages 8–12 years with nephrotic syndrome Child age group: Middle childhood, early adolescence	Quasi‐experimental	Informational, Physical, Emotional, and Practical: Nephrotic syndrome blended training program Intervention length: 4 sessions (30–45 min each) Intervention administrator: Researcher The nephrotic syndrome training program included sessions on each on the following: knowledge and awareness of patients about their illness, training on the recognition and observance of the recommended diet, the recognition and proper use of prescription drugs, identification of drug complications, recognition of recurrence symptoms, participation in sports activities and social activities, emotional control, and participation in educational activities	Persian Version of the Paediatric Quality of Life Generic Core Questionnaire. (PedsQL)	Children who received the blended training had significantly higher PedsQL scores than those who did not. The main difference in the quality of life in the intervention group before and after the intervention was related to physical performance and school performance
Li, Solomon, Zhang, Franklin, Ji & Chen [58], 2018, China	To examine the efficacy of solution‐focused brief therapy (SFBT) in a Chinese hospital for parental distress among parents with children with congenital heart disease (CHD)	Psychologically distressed parents (*N* = 40) of a hospitalized child with CHD Child age group: Unspecified/Various	Randomized controlled trial	Psychological and Emotional: Solution‐Focused Brief Therapy (SFBT) Intervention length: 4‐stuctured individual sessions Intervention administrator: Graduate‐level providers trained by two licensed clinical social workers This intervention shifts client's attention to their existing resources and solutions, increasing positive expectancies and emotions, like hope and optimism, to address feelings of sadness, worry, fear, and other emotions that are central psychological components of depression and anxiety. The study providers use questions to shift clients' focus to their strengths and past positive coping strategies and to identify if what they can do differently can alleviate psychosocial challenges in caring for their child	The Chinese version of the Brief Symptom Inventory‐18 (BSI‐18) The Chinese version of Herth Hope Index (HHI)	Results of the intent‐to‐treat analysis indicated a significant decrease in parental distress and increase in parents' levels of hope in the intervention group compared with the control group
Limperg, Haverman, Beijlevelt, Van Der Pot, Zaal, De Boer, Fijnvandraat, Peters & Grootenhuis [59], 2017, Netherlands	To give a description of psychosocial care provided by the multidisciplinary team of the Haemophilia Comprehensive Care Centre (HCCC) at the Emma Children's Hospital in Amsterdam, the Netherlands	Boys with haemophilia (BWH) (*N* = 100) and children with other congenital bleeding disorders (*N* = 130). Child age group: Term neonatal, infancy, toddler, early and middle childhood, early adolescence	Descriptive study	Practical, Emotional, Social, Informational, and Psychological: Various psychosocial supports offered by the multidisciplinary team Intervention length: Unspecified/Various Intervention administrator: Unspecified/Various Supports include monitoring and screening of health‐related quality of life (HRQOL) patient reported outcomes, psychoeducation via the 3‐day “Haemophilia Camp” and Haemophilia School, parent meetings, practical psychosocial care including employment securing and child life specialists, clinical interventions by paediatric psychologists, and individual tailored care by a social workers	N/A	No outcomes noted. Study described authors current program
Liu, Song, Zhu, Chen, Xie, Hu, Zeng & Tan [60], 2020, China	To examine the effectiveness of the family management style on improving the quality of family life in children with epilepsy	Children with epilepsy aged 0–6 years, parents aged 18 to 45 years (*N* = 130 families) Child age group: Term neonatal, infancy, toddler, early and middle childhood	Randomized controlled trial	Informational and Practical: Family management style Intervention length: Unspecified/Various Intervention administrator: Researcher, physicians, pharmacists, nurses Family management style combined with routine care was applied in the intervention group. It was implemented as follows: first, determine the family management plan considering the patients' disease condition and antiepileptic medications. Second, health education on family management style was provided at a guidance center, including the educating children and parents, personal guidance for disease knowledge, instructions on medication administration, assigning family tasks, identification of symptoms and treatment, home environment management (diet, exercise, sleep, study, etc.). Third, home self‐management was used by monitoring the application and effectiveness of this style at home by telephone, email, or WeChat messages every week after discharge	Beach Center Family Quality of Life Scale (FQOL)	FQQL scores were significantly higher for parents and children who received the family management style program compared to the control group. Specifically, family interaction, material well‐being, emotional well‐being, and disease‐related support scores improved in the intervention group, but no difference in the control group. The family management style can effectively improve the family quality of life in children with epilepsy, especially at the satisfaction level of family emotional well‐being and disability‐related support
Lopes‐Junior, Bomfim, Olson, Neves, Silveira, Nunes, Nascimento, Pereira‐da‐Silva & Lima [61], 2020, Italy, Israel, Brazil, Portugal, Canada, Columbia, Denmark, Germany, South Korea, and Spain34	To evaluate evidence from randomized controlled trials and non‐randomized controlled trials on the effectiveness of hospital clowns for a range of symptom clusters in children and adolescents admitted to hospital with acute and chronic conditions	24 studies including adolescents and children (*N* = 1612) in hospital who received hospital clown interventions Child age group: Unspecified/Various	Systematic review	Psychological and Emotional: Hospital clowns Intervention length: Unspecified/Various Intervention administrator: Hospital clowns Clown provide a complementary form of healthcare by using techniques such as music, juggling, improvisation, magic, storytelling, and puppetry to entertain children and adolescents in hospital; they help create a positive emotional state and environment that promotes interaction between parents and child and foster a hopeful attitude	Symptom management of inpatient children and adolescents	Studies showed that children and adolescents who were in the presence of hospital clowns, either with or without a parent present, reported significantly less anxiety during a range of medical procedures, as well as improved psychological adjustment. Three studies that evaluated chronic conditions showed favourable results for the intervention of hospital clowns with significant reduction in stress, fatigue, pain, and distress
Mai & Chaimongkol [62], 2022, Vietnam35	To determine the effectiveness of a Family Management Intervention Program (FMIP) on family quality of life and caregiver burden	Caregivers (*N* = 40) of children (4–9 years old) with autism Child age group: Early and middle childhood	Randomized controlled trial	Informational, Emotional, Psychological, and Social: The Family Management Intervention Program (FMIP) Intervention length: 4 1‐h sessions over 4 weeks Intervention administrator: Researcher The program content was adapted from the Building on Family Strengths program and guided by the FMSF. It included the following five content areas: basic knowledge related to autism and current interventions; managing the family caregivers' emotional dimensions; supporting family relationships, improving family communications and parenting; finding available resources; and transitioning into a more meaningful life	Beach Center Family Quality of Life (FQoL) Scale Caregiver's Strain Questionnaire (CSQ)	The findings revealed that the participants in the intervention group had a significantly higher family quality of life and significantly lower caregiver burden than those in the control group after attending the intervention and remaining overtime
Marshall, Pincus, Tesson, Lingam, Woolfenden & Kasparian [63], 2024, United States and United Kingdom	To synthesize and critically evaluate evidence on the effectiveness of integrated psychological care models for children with complex chronic illness within paediatric hospital settings and provide recommendations for successful implementation	15 studies (*N* = 2744 families) with children and adolescents with congenital or acquired heart disease, epilepsy, type 1 diabetes, cancer, inflammatory bowel disease, and neurocritical injury Child age group: Unspecified/Various	Systematic review	Psychological, Emotional, and Practical: Integrated psychological care models Intervention length: Unspecified/Various Intervention administrator: Mental health professional Examples of interventions named in studies include cognitive behavioural therapy, psychological distress and behavioural interventions, behavioural management strategies, problem‐solving skills training, parenting and relaxation techniques. Primarily led by a mental health professional (paediatric psychologist, psychiatrist, paediatric neuropsychologist)	Outcomes measured in studies include patient or parent physical, psychological, or behavioural outcomes, patient physical health outcomes, change in blood glucose level, health‐related quality of life (HRQOL). Various outcome measurement tools used	9 studies assess psychological service, 5 examined psychosocial screening, and 1 examined a neuropsychology service. 3 studies demonstrated the effectiveness of integrated psychological services in improving child or parent physical, psychological, or behavioural health outcomes. Integrated psychological services offering consultations at the same time and location as the child's medical visit reported the highest rates of uptake. The available evidence supports co‐location of child medical and psychological services
Melnyk, Alpert‐Gillis, Feinstein, Crean, Johnson, Fairbanks, Small, Rubenstein, Slota & Corbo‐Richert [64], 2004, United States	To evaluate the effects of a preventive educational‐behavioural intervention program, the Creating Opportunities for Parent Empowerment (COPE) program, initiated early in the intensive care unit hospitalization on the mental health/psychosocial outcomes of critically ill young children and their mothers.	Mothers (*N* = 163) with children (1–7 years old) admitted to the PICU Child age group: Toddler, early and middle childhood	Randomized controlled trial	Informational, Practical, and Psychological: COPE Intervention length: 3‐phase delivery, phase I in PICU, phase II in general ward, phase III post‐discharge Intervention administrator: Researcher via audiotapes, written information and workbook Program focused on increasing parents' knowledge and understanding of the range of behaviours and emotions that young children typically display during and after hospitalization and direct parent participation in their children's emotional and physical care. After discharge parents received a booster intervention that included a 5 min phone call that reinforced the children's typical postdischarge emotions and behaviours and parenting behaviours that would continue to facilitate positive coping outcomes for the children	State Anxiety Inventory (A‐State) Parental Stressor Scale: PICU (PSS:PICU) Involvement in Physical Care (VAS‐PC) Involvement in Emotional Care (VAS‐EC) Parental Beliefs Scale (PBS) The Post‐Hospital Stress Index (PSI‐C) Post‐Hospital Stress Index for Children (PSI‐P) Index of Parent Participation (IPP) Behavioural Assessment System for Children (BASC)	COPE mothers reported significantly less parental stress and participated more in their children's physical and emotional care on the paediatric unit, compared with control mothers, as rated by nurses who were blinded with respect to study group. COPE mothers reported less negative mood state, less depression, and fewer PTSD symptoms. COPE children exhibited significantly fewer withdrawal symptoms 6 months after discharge, as well as fewer negative behavioural symptoms and externalizing behaviours at 12 months.
Melnyk, Alpert‐Gillis, Hensel, Cable‐Beiling & Rubenstein [65], 1997, United States	To pilot test the effects of a theoretically driven intervention program, Creating Opportunities for Parent Empowerment (COPE), on the coping outcomes of critically ill children and their mothers	Mothers (*N* = 30) of children (1–6 years old) admitted to the PICU Child age group: Toddler, early and middle childhood	Two‐group experimental design	Informational, Psychological and Practical: Creating Opportunities for Parent Empowerment (COPE) program Intervention length: 2 phases, phase I in PICU, phase II in general ward Intervention administrator: Researcher via audiotapes, written information and workbook The COPE program consisted of two phases. Phase I occurred shortly after the child's admission to PICU and consisted of audiotaped information, also provided in written form, which focused on providing (a) child behavioural information which described young children's responses as they recovered from critical illness and (b) parental role information which gave mothers suggestions as to how they could facilitate their children's adjustment to hospitalization. Phase II of the COPE program occurred shortly after transfer from the PICU to the general paediatric unit. This “booster” intervention consisted of (a) audiotaped information which provided mothers with additional information on children's responses to hospitalization and how mothers could continue to enhance their children's adjustments; and (b) a parent–child activity workbook which contained three activities designed to assist parents in enhancing their children's coping during and following the stressful hospital experience	Index of Parent Support during Intrusive Procedures (IPS) Index of Parent Participation/Hospitalized Child (IPP) State–Trait Anxiety Inventory (STAI) Profile of Mood States (POMS) Paediatric Stressor Scale: Paediatric Intensive Care (PSS:PICU) Post‐Hospital Stress Index for Parents (PSI‐P) Post‐Hospital Behaviour Questionnaire (PBQ)	Mothers who received the COPE program provided more support to their children during intrusive procedures, provided more emotional support to their children, reported less negative mood state and less parental stress related to their children's emotions and behaviours, and reported fewer post‐traumatic stress symptoms and less parental role change 4 weeks following hospitalization.
Minor, Carlson, Mackenzie, Zernicke & Jones [66], 2006, Canada	To determine the changes in stress that might occur in a group of caregivers participating in mindfulness‐based stress reduction (MBSR) program for caregivers of children with chronic conditions	Caregivers (*N* = 44) of children (3–18 years old) with special needs and chronic conditions including diabetes, asthma, epilepsy, Down's syndrome, Chrone's disease, colitis, irritable bowel syndrome, cancer and attention deficit disorder Child age group: early and middle childhood, early adolescence	Pre‐post intervention design	Psychological, Physical, and Emotional: Mindfulness‐based stress reduction (MBSR) program Intervention length: 8‐week program, weekly 2‐h session in group setting Intervention administrator: Social worker and family physician The program consists of mindfulness meditation and yoga. Each session was composed of didactic teaching and discussion component around group themes and support for home practice, experimential practice of some form of meditation, and some form of yoga exercise, focusing on body awareness. Attitudes of non‐judging, patience, acceptance, trust, letting go, beginners mind, and non‐attachment were encouraged and reinforced. A program manual, mindful parenting manual, and two CDs were produced for the program. Each of the 8‐weeks had specific content	Profile of Mood States (POMS) Symptoms of Stress Inventory (SOSI)	Caregiver's stress decreased substantially over the 8‐week program, with an overall reduction in stress symptoms and in total mood disturbance
Mohammed, Mohamed & Zaki [67], 2021, Egypt	To assess the effect of psycho‐educational nursing intervention on coping strategies and psychological well‐being among family caregivers of children with Down Syndrome (DS)	Family caregivers (*N* = 60) of children with Down Syndrome Child age group: Unspecified/Various	Quasi‐experimental design	Informational, Emotional, Practical, and Psychological: Psycho‐educational program Intervention length: 2 part‐program through 33 sessions (in 25 h) Intervention administrator: Researcher The aim of the psycho‐educational program was to enhance coping strategies and psychological well‐being among family caregivers of children with Down Syndrome. Part 1: Theoretical, knowledge of down syndrome, family caregivers role in caring for he child, available services, types of coping strategies, and steps of effective coping Part 2: Practical, guidelines to deal with the child problems, modifying child behaviours, methods of effective coping and enhancement of psychological well‐being, anger management, and ways of expressing emotion	Interviewing Socio Demographic Questionnaire Brief‐COPE inventory Ryff's Psychological well‐being scale (1989)	Caregivers who received the psychoeducational program had significant positive effects on the well‐being of family caregivers, emotion‐focused coping strategies, active coping, positive reframing, problem‐focused coping strategies, humour, and acceptance, with decreased denial, self‐blaming, self‐distraction, and behaviour disengagement. Families with increased religious beliefs had increased psychological well‐being
Nabors, Stough, Combs, & Elkins [68], 2019, United States	To implement a new anxiety management manual for children and use qualitative methods to examine how children personalized strategies from the manual; and to examine parent perceptions of child coping	Children (*N* = 17 girls and 9 boys, ages 4–16) with chronic illnesses including: heart, gastrointestinal, Ehlers‐Danlos Syndrome, lung, and cancer. Parents of the children	Mixed methods	Emotional and Psychological: Anxiety management manual for children Interviewers reviewed the manual called ‘Coping Positively with my Worries Manual for Kids’ with the children while the parent observed. Children discussed how they would use different coping strategies and recorded this on the manual for use in the future The manual includes an overview of anxiety management strategies and coping strategies. Coping strategy include: (a) thinking and talking positively, (b) relaxation techniques including breathing and muscle relaxation, (c) distraction and doing fun things, and (d) imagination After reviewing the manual, children complete a coping menu that they keep. In the menu the children record worry triggers and coping strategies they can use	Parents listed their child's worries related to having a chronic illness and reported coping strategies their child was using and rated how often they helped their child reduce worries on a scale of 1–7 Parents rated how often they praise their child for dealing with their worries on a scale of 1–7 Parents listed coping strategies from the manual they thought their child would use in the future	Children personalized coping strategies taught in the anxiety coping manual. Parents felt their children were most likely to use relaxation and distraction strategies. Parents reported that children had some pre‐existing coping strategies, similar to those presented in the manual
[69] Pereira, MacDonald, Drobot, Bennett, Ali, Garros, 2021, Canada	To facilitate patient and family centered care at a Canadian Children's hospital by offering patients age‐appropriate, non‐medical interaction via volunteers and offering families guidance through their hospital experience via peer mentors (PM)	Paediatric intensive care unit (PICU) focus group: *N* = 5 health care professionals (HCPs) (nurses, occupational therapy and volunteer coordinator). PICU HCPs Survey: *N* = 120 PICU HCPs (allied health, unit clerks, social workers, health care aides, PICU administrator, occupational therapists, and pharmacist). Volunteer Surveys: *N* = 25 families of children admitted to PICU Peer Mentor Surveys: *N* = 21 families of children admitted to PICU Child age group: Unspecified/Various	Mixed methods program evaluation	Emotional, and Social: PICU peer and volunteer (P/V) program Intervention length: Unspecified/Various Intervention administrator: Peer mentors and volunteers Non‐medical interactions provided by Volunteers and Peer Mentors. Interactions with volunteers included cuddling, rocking, reading, playing, colouring, singing, crafting, or hand holding. Interactions with peer mentors included providing peer support, normalization, validation, and guidance throughout current PICU families' hospital stays	Primary outcome measures were patient and family experience and satisfaction. Four surveys were created for PICU HCPs and Family/Caregivers in collaboration by the PICU and evaluation teams	All stakeholder groups agreed that the PICU P/V Program was a valuable resource for PICU patients and their families. HCPs reported that they lack both time and training to provide regular developmental care to patients. However, the P/V Program may influence both families' and HCP's confidence in their ability to offer non‐medical interaction to children in the PICU
Rice, Matlack, Kristen, Simmons, Steinfeld, Laws, Dovey, Mark, & Cohen [70], 2015, United States	To evaluate the impact of LEAP, a volunteer‐based, inpatient asthma education program for families of inner‐city children with asthma	Children (*N* = 711) ages 2–17 years with status asthmaticus Child age group: Toddler, early and middle childhood, early adolescence	Randomized controlled trial	Informational: Lay‐educators for asthma program (LEAP) Intervention length: 1 (30–60 min) individualized, family‐based asthma education session Intervention administrator: Trained volunteer lay asthma educator The goal of the program was to decrease asthma morbidity by improving asthma knowledge, asthma‐related self‐efficacy, and asthma self‐management skills. Interactive education sessions included a guided discussion about asthma and diseases management, covering the following material: basics of asthma, trigger identification and avoidance, recognition of asthma flare‐ups, appropriate management responses, proper medication delivery, and importance of regular check‐ups	Paediatric Quality of Life Asthma (Peds QL SF22) Parent Asthma Management Self‐Efficacy Questionnaire	Families randomized to the intervention group were more likely to report use of a controller and a valved‐holding chamber, and were more likely to have an asthma action plan at follow up. Asthma self‐efficacy scores were significantly improved among those who received the intervention. Inpatient asthma education by trained lay volunteers was associated with improved asthma management behaviours
Sassmann, De Hair, Danne & Lange [71], 2012, Germany	To assess initial efficacy and feasibility of a structured behavioural group training (DELFIN) for parents of children with diabetes type 1 to reduce parenting stress and to improve parenting skills	Parents of children (2–10 year old) (*N* = 65; *N* = 33 mothers and *N* = 32 fathers) with type 1 diabetes Child age group: Toddler, early and middle childhood	Randomized controlled trial	Informational, Social, and Psychological: The DELFIN program Intervention length: Weekly 2‐h group sessions over a period of 5 weeks and receive an individual phone contact the week after Intervention administrator: Psychologist Structured group intervention for parents based on behavioural principles to strengthen their general and diabetes specific education competencies. Session content includes: processing dysfunctional cognitions, goal setting and theoretical discussion of communication skills, strategies to work on positive relationships with children, helpful parenting skills to solve typical family conflict, and practical skill training on challenges of families with children with chronic illnesses. Weekly homework was given at the end of each session	The Parenting Scale (PS 39) Questions to Education Behaviour Form (FZEV) The Depression‐Anxiety‐Stress Scale (DASS) Strengths and Difficulties Questionnaire (SDQ)	Parenting behaviour in conflict situations improved significantly after 3 months in the intervention group and control group. It remained stable over 12 months. Depression and anxiety scores of parents decreased for both groups. Even though the outcome in the intervention group was more positive, the differences between both study arms failed to reach statistical significance
Serlachius, Scratch, Northam, Frydenberg, Lee & Cameron [25], 2016, Australia	To evaluate a cognitive behaviour therapy–based program to improve glycaemic control and psychosocial well‐being in adolescents with type 1 diabetes	Adolescents (*N* = 147) aged 13 to 16 years with type 1 diabetes. Child age group: Early adolescence	Randomized controlled trial	Informational, Social, and Psychological: The Best of Coping (BOC) Intervention length: 5 2‐h weekly group sessions Intervention administrator: Health psychologists The BOC program is a cognitive‐behaviour therapy‐based program with key topics covering coping strategies, goal setting, positive and negative self‐talk, problem solving, and conflict resolution. The sessions included group discussion and written exercises. At the final session participants were given interactive CD‐ROMs to re‐enforce productive coping skills and maintain treatment effects	Glycemic control (HbA1c) collected at 3 and 12 months Stress levels were assessed by the Diabetes Stress Questionnaire For Youths (DSQ) Self‐efficacy was assessed by the Self‐Efficacy for Diabetes (SED) scale Quality of life was assessed by the Diabetes Quality of Life for Youth Scale (DQOL)	There was little evidence of differences in glycaemic control between groups. However, psychosocial well‐being improved in the intervention group compared to the control group
Sezer, Cavusoglu & Duzova [26], 2021, Turkey	To evaluate the effect of a self‐management program that was developed based on individual and family self‐management theory to aid adolescents with chronic kidney disease in the acquisition of competencies in the management of their disease	Adolescents (12–21 years old) (*N* = 50) with chronic kidney disease (CKD) Child age group: Early and late adolescence	Randomized controlled trial	Informational, and Physical: Self‐management training program Intervention length: 3 group sessions (every 15 days) (60–90‐min per session) Intervention administrator: Researcher The program consisted of three parts. The first part included information on definition, symptoms, diagnosis and treatment of the disease, and the side‐effects and usage of medications; the second part included information on nutrition and its role in kidney disease, as well as other considerations; and the third part included information on how to self‐monitor in CKD (weight control, blood pressure, side‐effects of medications and monitoring of laboratory findings, significance of regular follow‐ups) and its importance	Self‐management Assessment Form Paediatric Quality of Life Inventory‐Adolescent Form and Clinical Parameters (PedsQL) Self‐Efficacy Questionnaire for Children (SES‐C) Multidimensional Scale of Perceived Social Support (MSPSS) Beck Depression Inventory (BDI) Beck Anxiety Inventory (BAI)	A significant and positive change in all items on the self‐management assessment after the training occurred. Changes in blood pressure monitoring, planning health‐improvement activities, planning future oriented activities, and requesting information improved. Significant improvements in blood urea nitrogen levels, quality of life, and anxiety following the training were found for the intervention group compared to the control group
Simeone, Pucciarelli, Perrone, Rea, Gargiulo, Dell'Angelo, Guillari, Comentale, Palma & Vosa [72], 2017, Italy	To evaluate the efficacy of a nursing educational intervention in alleviating the level of parental anxiety in the parents of children who required heart surgery for the first time due to congenital heart disease	Parents (*N* = 60) of children with congenital heart disease. Child age group: Unspecified/Various	Qualitative, Comparative research design	Informational: Educational nursing intervention Intervention length: 1 session pre‐operatively Intervention administrator: Nurses The nurse provided explanations of the medical devices and equipment shown in the photographs. These explanations covered devices pertinent to the child's assistance, such as mechanical ventilator, drainage, cardiac monitoring, and blood‐pressure monitoring devices. The second step of the intervention was the presentation of a simulation of a “real case” from the time the child was admitted to hospital until the time of discharge	The State Trait Anxiety Inventory (STAI) (STAY‐1 examining state anxiety and STAY‐2 examining trait anxiety)	With regard to the anxiety level, the results of STAY‐1 showed that the average anxiety score of the experimental group was lower than that of the control group
Speedwell, Stanton & Nischal [73], 2003, United Kingdom	To investigate the impact that written information had on the stress levels of parents of visually impaired children. To find out who parents thought should provide the information; and at what stage they felt the information should be given	Parents (*N* = 77) of children who are visually impaired. Child age group: Early and middle childhood based on mean age (61.1 months)	Longitudinal, experimental intervention study	Informational: Parent education intervention Intervention length: 6 weeks given to parents to read booklet Intervention administrator: Researcher via booklet Providing information about the implications of visual impairment to parents of children with visual impairment via a booklet	The Perceived Stress Scale (PSS)	The results did not show an effect on levels of parental stress but did find that parents of school age children were more stressed than those of preschool age. Over 80% of participants considered that information was given too late and suggested it should be given soon after diagnosis. Of the controls, 32.6% thought the general practitioner should provide information on education although participants were more likely to expect the hospital to provide it
Svavarsdottir, Sigurdardottir & Tryggvadottir [74], 2014, Iceland	To evaluate the benefits of a two‐session family therapeutic conversation intervention (FAM‐TCIs) for families of children diagnosed with asthma, cancer, or diabetes	Families (*N* = 37) with children (0–18 years old) with chronic illnesses including cancer, asthma, and diabetes. Child age group: Term neonatal, infancy, toddler, early and middle childhood, early adolescence	Quasi‐ experimental, family level intervention study	Psychological, Emotional, and Informational: Two‐session family therapeutic conversation intervention (FAM‐TCIs) Intervention length: 2 therapeutic conversation interviews (45–90 min each) Intervention administrator: Nurses The FAM‐TCI consisted of two interview sections, given by a nurse, where the main focus of the intervention was based on the key elements of the brief family interview framework identified by Wright and Leahey (2013). The therapeutic conversations were offered as an opportunity for them to engage in therapeutic relationships. In the first session, the nurse drew a genogram and ecomap to identify existing family relationships. She then offered informative information regarding the child's health condition and asked therapeutic questions. Parents then described their experiences having an ill child. In the second session, the nurse asked interventive questions. Parents shared and reflected on their experiences, discussed worries and concerns, and expressed how they dealt with the situation	Iceland‐Expressive Family Functioning Questionnaire (ICE‐EFFQ) Iceland‐Family Perceived Support Questionnaire (ICE‐FPSQ) Disease‐specific QOL Questionnaire	Mothers of the children/teenagers perceived significantly higher family support after the FAM‐TCI compared with before; mothers reported significantly higher collaboration and problem‐solving abilities on the expressive family functioning scale after the FAM‐TCI. However, no significant differences were found on the fathers' perceived family support nor on their expressive family functioning after the FAM‐TCI compared with that before the intervention
Swallow, Carolan, Smith, Webb, Knafl, Santacroce, Campbell, Harper‐Jones, Hanif & Hall [75], 2016, United Kingdom	To (a) identify gaps in current online, chronic kidney disease (CKD) specific information and support, and determine the desirable components of the online parent information and support (OPIS) application, (b) develop the OPIS to address these identified needs and implement OPIS and (c) assess feasibility and methods in a small‐scale randomized controlled trial (RCT) of OPIS	Children (*N* = 39) with stage three, four, or five chronic kidney disease and their parents (*N* = 55) Child age group: Unspecified/Various	Mixed‐Methods	Informational, Social, and Psychological: Online parent information and support (OPIS) Intervention length: Mean 23.3 visits per user, lasting between 2 s to 58 min Intervention administrator: Computer‐based application Key OPIS components were defined as clinical support for care‐giving (information on treatment regimens, video‐learning tools, condition‐specific cartoons/puzzles, and a question and answer area), and psychosocial support for care‐giving (social‐networking, case‐studies, or testimonials, managing stress, and enhancing families' healthcare experiences)	Qualitative interviews Suitability Assessment of Materials (SAM) User Interface Satisfaction (USE) questionnaires	Twenty parents accessed OPIS. Responses from the SAM and USE questionnaires were positive, most respondents rating OPIS highly and finding it easy to use. Qualitative finding suggestions include refinement of OPIS components, enabling personalization of OPIS functionalities and proactive endorsements of OPIS by professionals
Swallow, Knafl, Santacroce, Campbell, Hall. Smith & Carolan [76], 2014, United Kingdom	To assess feasibility of a future full‐scale randomized controlled trial (RCT) of OPIS in terms of recruitment and retention, data collection procedures, and psychometric performance of the study measures in the target population, and investigate trends in change in outcome measures in a small‐scale RCT in parents of children with CKD Stages 3–5	Parents (*N* = 55) of children with chronic kidney disease Child age group: Unspecified/Various	Randomized controlled trial	Informational, Social, and Psychological: Online Parent Information Support (OPIS) application and program Intervention length: Mean 23.3 visits per user, lasting between 2 s to 58 min Intervention administrator: Computer‐based application The OPIS program comprises a mix of clinical care‐giving support (information on treatment regimens, video‐learning tools of multidisciplinary teams explaining how to undertake clinical procedures at home, condition‐specific cartoons/puzzles, and a question and answer area) and psychosocial support for caregiving (social networking, testimonials from other parents of children with CKD, and advice on managing stress). This was offered by password‐protected access to OPIS for 20 weeks	Rapid Estimate for Adult Literacy in Medicine (REALM) Family Management Measure (FaMM) Service System Subscale of the Family Empowerment Scale (FES) Dads Active Disease Support Scale (DADS)	Intervention group parents showed a greater improvement in perceived competence to manage their child's condition compared to control group parents. Differences between the groups in the FaMM Scale appeared to agree with a qualitative observation that OPIS helped parents achieve understanding and maintain awareness of the impact of their child's condition
Taiwo, Atilola, Ani & Ola [77], 2020, Lagos	To determine the effect of behavioural activation therapy on depression in adolescents living with Sickle Cell Disease (SCD) attending an out‐patient clinic	Adolescents (12–17 years old) (*N* = 60) with SCD with mild to moderate depression Child age group: Early adolescence	Randomized controlled trial	Informational, Psychological, Physical, and Emotional: Behavioural activation intervention program Interventions length: 5 structured weekly sessions of 45–60 min. Intervention administrator: Researcher The first session focused on psychoeducation on causes, symptoms and treatment of depression. The second session explained the rationale for behavioural activation. In the third session, pleasurable activities were identified, and participants were encouraged to have a list of pleasurable activities to carry out daily. The fourth session focused on relaxation techniques and participants were taught muscle relaxation, deep slow breathing exercises and positive imagery. This session also discussed psychosocial strategies for pain management. The fifth session was a revision of the preceding sessions and techniques	Mini International Neuropsychiatry Inventory Kid (MINI‐kid) Beck Depression Inventory‐II (BDI‐II) Paediatric Quality of Life (PedsQL)	There was a reduction at post intervention in the two groups on both BDI and PedsQL score measures. Only the treatment group showed statistically significant differences in the mean pre‐ and post‐intervention BDI scores. The findings showed that adolescents who received behavioural activation intervention had statistically significant reduction in depressive symptoms compared with the waitlist control group
Tan, Yin, Meng & Guo [78], 2021, China	To examine the effectiveness of sandplay therapy in reducing emotional and behavioural problems in school‐age children with chronic diseases as well as anxiety and depression in their caregivers	Children (6–12 years old) and their caregivers (*N* = 60) with leukaemia or chronic kidney disease Child age group: Middle childhood and early adolescence	Randomized controlled trial	Psychological and Physical: Sandplay therapy Intervention length: 6 sessions, 1–2 times a week for 60–90 min each Intervention administrator: Therapist The therapist guided the child to touch the sand and take five deep breaths to relax. The therapist particularly emphasized that the sandplay creation could be anything and that the child would not be judged. The therapist asked the child to introduce their sandplay creations, communicated with the child, and guided them to appreciate and explore their inner world	Child Behaviour Checklist (CBCL) Eysenck Personality Questionnaire (EPQ) Self‐ Rating Anxiety Scale (SAS) Self‐ Rating Depression Scale (SDS)	The total scores for CBCL, anxiety and depression, withdrawal, and social behavioural problems for children in the intervention group were all significantly lower than the corresponding scores for those in the control group. The EPQ scores for emotional stability and psychosis, SAS and SDS scores in the intervention group were both significantly lower than those in the control group
Thabrew, Stasiak, Hetrick, Donkin, Huss, Highlander, Wong & Merry [79], 2018, United States of America, Netherlands, China, Canada, Australia, England, Sweden, Serbia and Montenegro, South Africa53	To assess the effectiveness and acceptability of psychological therapies in comparison with controls for treating anxiety and depression in children and adolescents with long‐term physical conditions.	28 randomized control trials and 1 cross‐over trial. *N* = 1349 participants aged 4 to 21 years with long‐term physical conditions including chronic pain, abdominal pain, headaches, fibromyalgia, diabetes mellitus, inflammatory bowel disease, asthma, cancer, cardiac disease, epilepsy and human immunodeficiency virus (HIV) infection Child age group: Early and middle childhood, early and late adolescence	Systematic Review	Psychological and Emotional: Various psychological interventions. Intervention length: Unspecified/Various Intervention administrator: Unspecified/Various Interventions included behaviour therapies, cognitive behaviour therapies (CBT), third wave CBTs, psychodynamic therapies, humanistic therapies, integrative therapies, systemic therapies, bibliotherapy, and art therapy	Various measures and tools to measure treatment efficacy, treatment susceptibility, changes in caseness, suicide‐related behaviour, quality of life, psychological well‐being, long‐term physical conditions, adherence to treatment, school attendance and economic benefits	There was inadequate evidence to determine if the interventions impacted anxiety, Quality of Life, physical symptoms, functioning, treatment efficacy, and depression. Psychological interventions designed to reduce anxiety or depression were more effective than psychological therapies designed to improve other symptoms or general coping. There was inadequate, low‐quality evidence to determine if psychological therapies were more effective than controls at improving functioning
Tomaj, Estebsari, Taghavi, Nejad, Dastoorpoor & Ghasemi [80], 2016, Iran	To determine if group play therapy could significantly increase self‐concept among children with thalassaemia major ages 7 to 11 years old in teaching hospitals	Children (7–11 years old) (*N* = 60) with thalassaemia major Child age group: Middle childhood	Randomized controlled trial	Social and Psychological: Group play therapy Intervention length: 8 sessions of group games (45–60 min each) Intervention administrator: Researcher Children in the intervention group were divided into groups of seven to eight. They took part in eight sessions of group games	Piers‐Harris Children's Self‐Concept Scale	For the intervention group, results showed that the mean self‐concept score was significantly higher at the second point in time compared to the baseline, and continued to increase in the second and third time points. For the control group, comparing the first, second, and third time points did not result in any significant change in the mean score
Uhm & Kim [81], 2019, South Korea	To identify the effects of a mother–nurse partnership program based on the core components of information sharing, negotiation and participation in care	Mothers of infants (*N* = 73) who received surgery for congenital heart disease. Child age group: Preterm neonatal, term neonatal and infancy	Quasi‐experimental study	Informational: Mother‐nurse partnership program (MNPP) Intervention length: Individually delivered (30‐min) twice a day in five phases. Intervention administrator: Nurses First phase being orientation, second to fifth phase included information sharing, negotiation, and participation. Primary nurses provided mothers with a tailored factsheet about the status of their infant. Clinical nurse specialists provided mothers with information they wanted to know more about. Activities nurses taught mothers included: touching, hygiene, diaper changing, feeding, and holding	Shortened EMpowerment of PArents in THe Intensive Care 3‐ Questionnaire (EMPATHETIC) Karitane Parenting Confidence Scale Parent–nurse Partnership Scale (PNPPS‐PN) State Anxiety Scale (STAI)	Compared with controls, experimental group mothers reported significantly higher parental satisfaction, parental self‐efficacy, perceived partnership and lower anxiety, upon transfer to the ward. Infant outcomes did not differ between the groups
Westrupp, Northam, Lee, Scratch & Cameron [82], 2015, Australia	To test the efficacy of the Triple P‐Positive Parenting Program in reducing or preventing mental health problems and improving glycemic control in children with type 1 diabetes	Parents (*N* = 76) of children with type 1 diabetes. Child age group: Middle childhood (from mean age of 9.20 years old)	Randomized controlled trial	Psychological and Informational: Triple P‐Positive parenting program Intervention length: 10 individual 1‐h weekly sessions Intervention administrator: Clinical psychologist During the sessions, a didactic style was used to teach parents 17 strategies; 10 promoting children's competence and development, and 7 helping parents manage misbehaviour. Parents received two books and were asked to complete homework tasks	The Parent Rating Scale (PRS) of the BASC‐2 Depression Anxiety Stress Scale (DASS) Parenting Scale (PS) Parenting Sense of Competency Scale (PSOC) Parent Problem Checklist (PPC) Eyberg Child Behaviour Inventory (ECBI) Diabetes Family Conflict Scale Relationship Quality Index (RQI)	Benefits of Triple P were evident at 3 months for parent mental health, parenting skills, and family functioning, but not for child mental health or glycemic control, with little effect at 12 months. Improvements in parent mental health and parenting competency associated with Triple P were sustained to 12 months for children with pre‐existing mental health problems. This study provides some support for the efficacy of Triple P in improving parent and family outcomes, and reducing child internalizing and externalizing behaviour problems primarily in children who have pre‐existing mental health problems
Wu, Li, Huang, Huang, Xiao, Chi, Feng & Yang [83], 2024, China	To evaluate the effects of a nurse‐led cognitive behavioural intervention for parents of children with epilepsy (CWE)	Parents (*N* = 387) of children (0–18 years old) with epilepsy Child age group: Term neonatal, infancy, toddler, early and middle childhood, early adolescence	Longitudinal study	Psychological, Emotional, and Informational: Cognitive and behavioural intervention Intervention length: 1 month duration, delivered two times a week for 30 to 40 min Intervention administrator: Nurses The cognitive intervention was conducted in the first 2 weeks, the behavioural intervention was conducted in the third week, and the feedback and reinforcement were completed in the final week. *Cognitive intervention*: one‐to‐one conversations to develop trusting relationships, explained epilepsy‐related knowledge, ascertained the importance of medication, talked about the parents' negative emotions, and encouraged the parents to seek psychological help. *Behavioural intervention*: medication behaviour training, mastering skills of medication administration. Also, music therapy to relax patients, and education on breathing exercises. *Feedback and reinforcement*: children received rewards for medication adherence. Examination of parents' epilepsy knowledge, and targeted cognitive and psychological interventions based on their cognitive and psychological status	Evaluation of seizure severity, treatment compliance, and satisfaction with care provided by nurses State–Trait Anxiety Inventory (S‐AI) Center for Epidemiologic Studies Depression Scale (CES‐D) Pittsburgh Sleep Quality Index (PSQI) Chinese version of the Public Attitudes Toward Epilepsy Scale (CPATE)	The follow‐up 6 months after discharge showed that the seizure frequency among CWE in the intervention group was significantly less than the controls. Compared with the controls, the intervention group also reported significantly fewer symptoms of anxiety and depression, better sleep quality, and more positive attitudes toward epilepsy, as well as higher nursing satisfaction. The correlation analysis indicated the correlation of CWE's seizure severity was correlated with the compliance, parents' psychological states, and parents' satisfaction with the care provided by nurses
Yoo, De Gagne, Jeong & Jeong [84], 2018, South Korea	To develop a hybrid atopic dermatitis (AD) education program and evaluate its effects on anxiety, caregiving efficacy and caregiving behaviour among mothers of children with AD	Mothers (*N* = 23) of children with atopic dermatitis Child age group: Unspecified/Various	Quasi‐experimental study	Informational: Atomic dermatitis management program Intervention length: 8 weeks of hybrid learning Intervention administrator: Dermatologist, nurse, nurse assistant, and program coordinator An intervention team developed and delivered the hybrid learning program for 8 weeks, comprising both offline and online education components	Anxiety Measurement Tool Parenting Sense of Competence Scale (PSOC) Korean measuring tool to measure parents' at‐home caregiving behaviours	After the intervention, mothers' anxiety reduced, and caregiving efficacy and behaviour improved significantly

**TABLE 3:** Characteristics of included studies.

Characteristics	No. of studies	Studies
Study method		
Qualitative		
Descriptive/framework discussion	2	[38, 59]
Quantitative		
Randomized controlled trial	26	[25, 26, 28, 29, 30, 31, 32, 34, 42, 44, 46, 48, 49, 50, 55, 58, 60, 62, 64, 70, 71, 76, 77, 78, 80, 82]
Pre‐ and post‐design	2	[35, 66]
Clinical trial	1	[39]
Unspecified	1	[37]
Quasi‐experimental	8	[41, 54, 56, 57, 67, 74, 81, 84]
Two‐group experimental	1	[65]
Comparative research design	1	[72]
Longitudinal	2	[73, 83]
Mixed Methods	5	[40, 52, 68, 69, 75]
Review		
Systematic Review	7	[33, 47, 51, 53, 61, 63, 79]
Scoping review	1	[36, 39]
Case report	1	[43]
Country		
Italy	3	[28, 61, 72]
Turkey	3	[26, 29, 42]
Jamaica	1	[30]
Egypt	2	[31, 67]
Iran	10	[32, 34, 36, 46, 50, 53, 54, 55, 57, 80]
United Kingdom	9	[33, 36, 47, 52, 63, 73, 75, 76, 79]
United States	15	[33, 36, 37, 38, 41, 43, 47, 51, 53, 63, 64, 65, 68, 70, 79]
Canada	9	[33, 36, 39, 40, 53, 61, 66, 69, 79]
Australia	6	[25, 35, 36, 51, 79]
Germany	4	[36, 49, 61, 71]
Switzerland	1	[36]
Iceland	2	[36, 74]
Sweden	2	[36, 79]
Chile	1	[36]
China	6	[36, 58, 60, 78, 79, 83]
Denmark	1	[36]
Japan	1	[36]
Malaysia	1	[36]
Mexico	1	[36]
New Zealand	1	[36]
Spain	2	[36, 61]
Thailand	1	[36]
Hong Kong	2	[45, 51]
France	2	[47, 53]
Netherlands	3	[48, 59, 79]
India	1	[56]
Israel	1	[61]
Brazil	1	[61]
Portugal	1	[61]
Columbia	1	[61]
Denmark	1	[61]
South Korea	3	[61, 81, 84]
Vietnam	1	[62]
Lagos	1	[77]
Serbia and Montenegro	1	[79]
South Africa	1	[79]
Participants		
Mothers	11	[28, 30, 32, 34, 40, 46, 54, 64, 65, 81, 84]
Parents and caregivers	24	[29, 35, 37, 38, 41, 45, 51, 52, 53, 58, 59, 60, 62, 66, 67, 68, 71, 72, 73, 75, 76, 78, 82, 83]
Children and adolescents	27	[25, 26, 29, 31, 32, 33, 39, 42, 47, 48, 49, 50, 51, 53, 55, 56, 57, 60, 61, 68, 70, 75, 77, 78, 79, 80]
Families	3	[43, 63, 74]
Health care professionals	2	[39, 69]
Intervention type		
Emotional and/or psychological	47	[25, 28, 29, 31, 32, 33, 34, 35, 36, 38, 39, 40, 41, 43, 44, 45, 47, 48, 49, 50, 51, 52, 53, 55, 56, 57, 58, 59, 61, 62, 63, 64, 65, 66, 67, 68, 69, 71, 74, 75, 76, 77, 78, 79, 80, 82, 83]
Informational	43	[25, 26, 28, 29, 30, 32, 33, 34, 35, 36, 37, 38, 42, 43, 44, 45, 46, 48, 49, 50, 51, 52, 54, 56, 57, 59, 60, 62, 64, 65, 67, 70, 71, 72, 73, 74, 75, 76, 77, 81, 82, 84]
Social	21	[25, 28, 30, 33, 36, 38, 40, 41, 43, 45, 47, 48, 52, 56, 59, 62, 69, 71, 75, 76, 80]
Spiritual	1	[34]
Practical	11	[30, 36, 37, 47, 57, 59, 60, 63, 64, 65, 67]
Physical	10	[26, 29, 31, 33, 37, 55, 57, 66, 77, 78]
Intervention target		
Mothers	9	[28, 30, 32, 34, 46, 64, 65, 81, 84]
Parents and caregivers	22	[29, 35, 36, 37, 38, 41, 44, 45, 54, 58, 66, 67, 71, 72, 73, 75, 76, 83]
Children and adolescents	26	[25, 26, 31, 32, 33, 42, 47, 48, 49, 50, 51, 52, 53, 55, 56, 57, 59, 61, 64, 68, 77, 78, 79, 80, 82]
Families	11	[39, 40, 43, 52, 53, 60, 62, 63, 69, 70, 74]
Intervention administrator		
Psychologist	7	[25, 28, 45, 48, 50, 71, 82]
Occupational therapist	1	[29]
Nurses	10	[30, 34, 37, 39, 60, 72, 74, 81, 83, 84]
Researcher	15	[26, 31, 34, 42, 44, 46, 50, 55, 57, 60, 62, 67, 68, 77, 80]
Genetic counsellor	1	[35]
Social workers	3	[38, 58, 66]
“Psychological, educational and medical staff”	1	[49]
Computer‐based application	3	[40, 75, 76]
Peer leaders/parent mentors	3	[41, 43, 69]
Physicians	3	[60, 66, 84]
Pharmacists	1	[60]
Hospital clowns	1	[61]
Mental health professional	1	[63]
Written information/Audiotapes	3	[64, 65, 73]
Volunteers	2	[69, 70]
Therapist	1	[78]
Nurse assistant & program coordinator	1	[84]
Unspecified/Various	11	[32, 33, 36, 47, 51, 52, 53, 54, 56, 59, 79]
Mode of Intervention Delivery		
Face‐to‐face/In‐person	38	[25, 26, 28, 29, 30, 31, 32, 34, 35, 37, 38, 45, 46, 48, 49, 50, 51, 52, 55, 56, 57, 58, 59, 61, 62, 63, 66, 67, 68, 69, 70, 72, 74, 77, 78, 80, 81, 83]
Mixed (face‐to‐face, remote via telephone or virtual methods, computer‐based, workbook‐based)	15	[33, 39, 42, 43, 44, 47, 53, 54, 64, 65, 71, 79, 82, 84]
Unspecified	1	[36]
Computer‐based	3	[40, 75, 76]
Remote (Zoom)	1	[41]
Booklet	1	[73]
Length of Intervention		
Number of sessions		
1	5	[31, 35, 37, 70, 72]
2–5	19	[25, 26, 30, 32, 34, 38, 39, 43, 45, 46, 55, 57, 58, 62, 64, 65, 71, 74, 77]
6–8	8	[38, 48, 50, 56, 66, 78, 80, 83]
9–10	5	[29, 42, 49, 81, 82]
11–15	1	[41]
16+	3	[67, 75, 76]
Unknown/Various	18	[33, 36, 40, 44, 47, 51, 52, 53, 54, 59, 60, 61, 63, 68, 69, 73, 79, 84]
“Weekly” with no specified period	1	[28]
Length of sessions		
< 1 h	15	[29, 32, 37, 42, 55, 57, 67, 70, 74, 75, 76, 77, 80, 81, 83]
1–2 h	18	[25, 26, 28, 30, 34, 35, 37, 41, 45, 46, 49, 50, 62, 66, 71, 74, 78, 82]
> 2 h	1	[39]
Unknown/Various	27	[31, 33, 36, 38, 40, 43, 44, 47, 48, 51, 52, 53, 54, 56, 58, 59, 60, 61, 63, 64, 65, 68, 69, 72, 73, 79, 84]
Child Age Group		
Preterm neonatal (born before full gestational period)	1	[81]
Term neonatal (birth—27 days)	9	[28, 33, 38, 40, 59, 60, 74, 81, 83]
Infancy (28 days—12 months)	10	[30, 33, 38, 40, 47, 59, 60, 74, 81, 83]
Toddler (13 months—2 years)	17	[33, 34, 38, 39, 40, 44, 46, 47, 51, 60, 64, 65, 70, 71, 74, 83]
Early childhood (2–5 years)	26	[29, 31, 33, 34, 38, 39, 40, 44, 46, 47, 51, 52, 54, 59, 60, 62, 64, 65, 66, 68, 70, 71, 73, 74, 79, 83]
Middle childhood (6–11 years)	36	[29, 31, 32, 33, 38, 39, 40, 44, 45, 46, 47, 49, 50, 51, 52, 53, 54, 55, 56, 57, 59, 60, 62, 64, 65, 66, 68, 70, 71, 73, 74, 78, 79, 80, 82, 83]
Early adolescence (12–18 years)	30	[25, 26, 29, 32, 33, 38, 39, 40, 42, 45, 48, 49, 50, 51, 52, 53, 54, 55, 56, 57, 59, 66, 70, 71, 74, 77, 78, 79, 83]
Late adolescence (19–21 years)	7	[26, 38, 40, 45, 47, 68, 79]
Unspecified/Various	14	[35, 36, 37, 41, 43, 58, 61, 63, 67, 69, 72, 75, 76, 84]
Child Diagnosis		
Congenital	6	[28, 39, 58, 68, 72, 81]
Neurological	10	[29, 37, 39, 45, 46, 47, 52, 54, 60, 79, 83]
Haematological	8	[30, 31, 34, 50, 56, 59, 77, 80]
Nephrological	7	[26, 32, 39, 57, 75, 76, 78]
Various/Unspecified	12	[33, 36, 41, 47, 51, 53, 55, 61, 63, 64, 65, 69]
Cardiac	6	[35, 40, 58, 68, 72, 81]
Genetic	4	[38, 39, 66, 67]
Orthopaedic	1	[39]
Gastrointestinal	6	[39, 43, 48, 66, 68, 79]
Metabolic	7	[25, 39, 66, 71, 74, 79, 82]
Birth defect	1	[39]
Respiratory	5	[42, 49, 70, 74, 79]
Neurodevelopmental	2	[62, 66]
Oncology	5	[66, 68, 74, 78, 79]
Ophthalmologic	1	[73]
Immunological	1	[79]
Dermatological	1	[84]

### 3.2 Emotional and Psychological Interventions

#### 3.2.1 Discussion and Verbalization

Interventions within the emotional and psychological care domains often used a discussion‐type format during the delivery, where children and parents verbally described and discussed their emotions, experiences, feelings, concerns, challenges, worries, and doubts [28, 29, 34, 38, 68, 74]. Other discussion topics included parents' experiences of having a sick child [74], consequences of the disease, stress, anxiety, anger management [34, 39], and effects on family life [52]. Storytelling helped children express their feelings of being different because of their disease [48]. In one computer‐based application intervention, parents and children described what it was like to live with a medical condition in experience journals [40]. Discussions helped foster communication skills between parents and their children [71] and the development of trusting relationships between the nurse interventionist and parents so parents could express their negative emotions [83]. Discussion and verbalization occurred either in a group setting or 1:1 with parent/child and HCP.

#### 3.2.2 Mindfulness

Interventions that utilized mindfulness techniques included education on how to use relaxation skills [31, 48, 53, 77], classical relaxation music [31], music therapy, breathing exercises [83], muscle relaxation, deep breathing, and positive imagery [77]. Mindfulness interventions could be in the form of yoga [66] or sand play with children, where a therapist guided children to build a sand creation and encouraged the children to take 5 deep breaths when they touched the sand [78].

#### 3.2.3 Emotional Care

Some interventions focused on enhancing the emotional health of children and their parents [58]. In one support group intervention, parents engaged in group therapy with the hopes of enhancing their positive emotions [45]. A patient education program aimed to enhance children's acquisition of emotion‐focused coping skills [49]. Some interventions used educational strategies to teach children and parents how to manage their emotions, including information on negative emotions and their control [50, 57, 62], while another aimed to increase parents' knowledge and understanding of common emotions children experience when admitted to the hospital (e.g., sadness, worry, and fear) and how to provide emotional care to them [64, 65].

#### 3.2.4 Cognitive Behavioural Therapy

A variety of interventions utilized the psychological approach of Cognitive Behavioural Therapy (CBT) [41, 53, 79]. For example, one intervention used cognitive techniques to promote positive thinking by identifying and challenging inaccurate thoughts [48], while another multimodal education program used cognitive restructuring [49], and yet another intervention provided information on reasonable thinking [50]. Some interventions aimed to shift parents' attention to resources and solutions, increasing their positive expectancies [58, 71]. One CBT therapy‐based program covered key topics such as coping strategies, goal setting, positive and negative self‐talk, problem solving, and conflict resolution [25].

#### 3.2.5 Behaviour

There were interventions aimed at changing the behaviours of parents and children. For example, one program integrated behavioural procedures into their intervention, including modelling, exposure exercises, and contingency management [48]. The COPE intervention aimed to increase parents' knowledge and understanding of their children's behaviours during hospitalization and instruct them on how to care for them and respond to their needs [44, 54, 64, 65]. A psycho‐educational program provided parents with practical guidelines on how to handle their children's problems and modify their behaviours [67]. Finally, a didactic intervention taught parents how to manage their children's misbehaviour [82].

#### 3.2.6 Stress and Coping

Various interventions aimed to address parents' and children's stress and help them cope. This was accomplished using a variety of approaches, including educating children how to cope with disease‐specific stressors (e.g., stress management training) [49], offering online programs that inform parents and children how to manage stress [75, 85], and parents receiving reinforcement of positive coping skills during a parent support group [56]. During a therapy session, parents were asked questions about their past positive coping strategies to help improve psychosocial challenges in caring for their child [58]. Parents were provided theoretical knowledge on types of positive coping strategies [67] and CBT was used to enhance parents' coping during therapy sessions [25]. One anxiety management intervention encouraged children to read a manual about coping and describe how they used various coping strategies, while parents were encouraged to praise their children for dealing with their worries [68]. Finally, a stress‐point intervention helped families identify stressful issues about hospitalization and develop a plan to help parents handle the issues [39].

#### 3.2.7 Family Dynamics

There were interventions that addressed and strengthened family dynamics. For example, one intervention included group meetings with parents and HCP where parents discussed the effects of the children's disease on family life [52]. Another intervention offered parents strategies on how to create positive relationships with children, solve family conflict, and address challenges of having a child with a chronic illness [71]. In a family therapy intervention delivered by a nurse, families engaged in therapeutic conversations and identified existing family relationships with extended family [74].

#### 3.2.8 Strength‐Based and Empowerment

Some interventions provided parents with coaching on how to identify their children's and their own strengths and empower them to use them [45]. For example, during therapy sessions, parents were asked questions to shift their focus to their strengths and address challenges they experienced caring for their child [58]. In one study for parents with persistent or escalating distress, counselling was offered to empower parents and children on how to manage their circumstances and challenges and improve their health‐related quality of life [59]. Other ways parents and children were empowered included: rewarding children for medication adherence [83], praising families' strengths and successes [39], teaching parents how to promote their children's competence [82], providing individualized family‐centered education [56], and helping parents develop a concrete plan to utilize children's strengths to resolve problems [45].

### 3.3 Informational Interventions

#### 3.3.1 Teaching Disease‐Specific Skills

The majority of interventions that included an informational approach provided parents with education that was disease‐specific. This included interventions that offered parents and children with information of the disease itself [26, 34, 48, 49, 52, 57, 59, 60, 62, 67, 73, 74, 84], treatment [37, 49, 50, 60, 62, 71, 75, 84, 85], triggers [49, 70], self‐care techniques (children's independent management) [33, 49, 84], complications [34, 46, 50, 56, 57], diet [26, 34, 56, 57], activity [34, 50, 57, 60], causes of disease relapse [46], caregiving skills [28, 81], problem solving [46], administration of medications [26, 46, 57, 59, 60, 70, 83, 84], and symptom evaluation and monitoring [26, 37, 46, 57, 60, 70, 84]. Other disease‐specific topics included in informational interventions were: hygiene, vaccination, prevention of infections [56], family management style [60], home management [60, 75, 85], sleep [60], check‐up schedules [70], medical equipment and devices (ventilator, cardiac monitoring, blood pressure device) [72], genetic counselling [35], and a fact sheet about infants' status [81]. In one study, veteran parents provided parents of newly diagnosed children with information on resources for travel, school, and other useful medical education materials [43]. Finally, a video‐feedback intervention taught parents how to engage in occupational therapy with their children [29].

#### 3.3.2 Teaching Parenting Skills

There were interventions geared toward teaching parents parenting‐focused skills [36]. For example, two interventions helped parents promote their children's development [30, 82], one support intervention reinforced positive parenting skills [28], and one parenting program provided parents with strategies to promote children's competence [82]. Another intervention included teaching parents how their children might respond to hospitalization and how they can support their children's needs [44]. Three interventions taught parents how to identify children's problem behaviours and strategies to manage them [67, 77, 82]. Handouts, experiential exercises, videos, and questionnaires were used in one intervention to coach parents on how to identify and utilize their children's character strengths to increase their positive emotions [45]. Finally, one intervention provided parents with a mindful parenting manual [66].

#### 3.3.3 Psychological and Emotional Education

Some interventions educated parents and children on strategies to cope with and manage their mental and emotional health [47]. Specific areas of education included: acceptance [34, 41], social support [34], coping strategies [25, 67, 68], ways to express emotion [67], positive self‐talk [25], relaxation techniques [34, 66, 68, 77, 83], processing feelings of guilt [71] and stress management [49]. In one psychoeducational group intervention, adolescents learned about social competence by engaging in storytelling [48]. In a training program, children and adolescents were taught about emotion control [57], and in one intervention, parents were taught about their children's emotions that they could feel when admitted to hospital and how to care for them [64, 65]. Finally, one psychoeducational intervention provided education on the causes, symptoms, and treatment of depression, along with how to relax using music, deep breathing, pleasurable activities, and positive imagery [77].

### 3.4 Social Interventions

A variety of interventions were delivered in a group setting to create a social community among parents and children where they could support one another [25, 30, 36, 38, 47, 52, 56, 71, 75, 85]. For example, one intervention focused on group play therapy for children [77], and another computer‐based application encouraged children and families to describe their experiences living with a medical condition in writing, with other patients and families able to read their written descriptions [40]. In two studies, veteran parents fostered social connections by leading group discussions, providing social supports [41], and offering mentorship (e.g., affirmational, informational, and emotional support) for parents admitted to hospital [43]. In one intervention, volunteers and peer mentors cuddled, played with children, provided peer support, validated parents, offered guidance, and normalized the situation [86]. In a 3‐day Haemophilia camp, patients and families came together to learn together and provide one another with support [59].

### 3.5 Spiritual Intervention

Few studies included the spiritual care domain. One group‐based training program included spiritual considerations for families [34]. Another program management intervention aimed to help parents and children transition to a more meaningful life [62]. According to Tanyi [87], a spiritual connection can help increase a sense of hope for patients and families. Outcomes of two of the interventions were increased hope for parents [45, 58]. A systematic review found that hospital clowns can be used to foster a hopeful attitude among parents and children [61].

### 3.6 Practical Interventions

A few interventions addressed practical issues that families face when parenting a child with a chronic disease. These included advocacy for healthcare services and resources [56, 62, 67]. One intervention included an online guide where a social worker offered parents answers and tips regarding financial aspects, transportation, employment, housing, and health insurance [59]. A systematic review explored interventions that included a support worker to connect children and parents to needed financial support [47].

### 3.7 Outcomes of the Interventions

A majority (*N* = 49) of the interventional studies reported positive results across outcome measures (see Table 1 for outcome measures and study findings), demonstrating their benefits for both children and parents. The most reported positive outcomes included improvements in the following: health and well‐being (e.g., decreased stress, increased coping, improved children's physical, psychological, and behavioural health, and improved mental health for children and parents), disease management, family functioning, and satisfaction with hospital care. See Table 4 for a complete list of positive outcomes reported in the studies.

**TABLE 4:** Positive outcomes of interventions.

Outcome domain	Outcomes
Health and well‐being	– Decreased social isolation [40] – Increased coping [29, 39, 48, 49, 50, 67, 68, 79] – Decreased stress [29, 30, 31, 41, 45, 53, 61, 64, 65, 66] – Improved emotions and emotional skills [29, 41, 63, 78] – Improved hope [45] – Improved self‐esteem [48, 80] – Improved stress management [49] – Improved children's physical, psychological, and behavioural health [26, 47, 51, 61, 63, 64, 79, 82, 83] – Improved well‐being [25, 47, 61, 67] – Increased perceived control with guilt, shame, depression, anxiety, and stress [35] – Decreased parental and/or children's anxiety, depression, distress, and PTSD [26, 28, 30, 32, 41, 42, 51, 53, 54, 58, 61, 63, 64, 65, 66, 71, 72, 77, 78, 79, 81, 82, 83, 84] – Increased self‐efficacy [26, 42, 46, 70, 81, 84] – Improved quality of life [33, 48, 56, 60, 62, 63] – Decrease fear [55, 61] – Improved adaptive behaviours [34] – Decreased social behaviour problems [78] – Improved sleep quality [83] – Increased understanding of family and children's feelings about the disease [40] – Improved social support [45, 47, 53]
Disease management	– Improved ability to manage disease [26, 29, 37, 46, 64, 70, 75, 84, 85] – Improved compliance [83] – Improved knowledge of the disease [39, 46] – Increased transition readiness [42] – Improved problem‐solving abilities [74] – Positive attitudes toward the disease [83]
Family functioning	– Satisfaction with family functioning [39, 82] – Perceived family support [74] – Decreased caregiver burden [32, 34, 62] – Improved parental ability to provide emotional care [64, 65] – Improved parenting skills [71, 82]
Hospital care	– Reduce emergency room use [33] – Improved emergency care [46] – Higher nursing satisfaction [83] – Improved satisfaction with nursing care [83] – Improved information received [47] – Improved communication with healthcare professionals [39] – Increased confidence and comfort voicing concerns [26, 43, 44] – Satisfaction with the intervention [35, 47, 53, 75, 81, 85]

Not all studies had positive outcomes across all measures. For example, in Asani and colleagues [30] group problem‐solving intervention, mothers did not experience changes to their problem‐solving skills, coping behaviours, or depressive symptoms as anticipated, and Duffy and Vessey [44] COPE intervention did not improve parents' anxiety or depression. In Hoare and Kerly [52] group counselling intervention, parents did not like the group discussion but preferred one‐on‐one counselling. Other psychosocial interventions did not have anticipated outcomes, including lack of positive changes to: glycemic control [25, 82], parental stress [73], family support [74], paediatric quality of life [77], and children's mental health [82]. Thabrew and colleagues [88] found in their systematic review that the use of psychological therapies among children with long‐term physical conditions had inadequate evidence to determine if they were effective. Hughes and colleagues [53] systematic review provided limited support for interventions aimed at mindfulness. A systematic review looking at self‐care interventions demonstrated minimal effects on quality of life and emergency room use [33]. Finally, one information intervention lacked promising outcomes because information was given too late; parents suggested that information be provided soon after diagnosis [73].

### 3.8 Harms, Adverse Events, Risks

Minimal harms and no adverse events were reported. Some parents experienced negative feelings or feelings of overwhelm when exposed to other parents' problems in a group setting [36, 40]. In Swallow and colleagues' [76] online support program, some parents experienced increased family life difficulty. Lastly, in Westrupp and colleagues' [82] parenting program, parents reported lower parenting self‐efficacy after 3 months of receiving the intervention. None of the studies reported potential or actual risks involved with the interventions.

### 3.9 Relevance to European Countries

Six interventional studies were completed in countries within the European Union (EU), including Italy, Germany, and the Netherlands [28, 48, 49, 59, 71, 72], and the reviews included in this scoping review contained studies from a variety of European countries. Psychosocial support interventions within the EU were administered by healthcare professionals (e.g., psychologists and nurses), rather than by volunteers or peers and did not utilize technology‐based approaches. All interventions provided detailed information about the disease to parents, along with comprehensive strategies to offer parents psychological support for their mental health and stress. Therefore, interventions within EI countries demonstrated a tendency toward comprehensive, professionally delivered, structured psychosocial support interventions that integrated multiple aspects of care within their established healthcare systems.

## 4 Discussion

This scoping review included 59 articles and provided a detailed overview of the available evidence on psychosocial supports offered to children with a chronic disease admitted to hospital and their parents. Psychosocial interventions included in this review were multimodal, encompassing a range of care domains to help children and their parents manage their health and well‐being, including: emotional and psychological, informational, social, spiritual, and practical interventions. Most of the studies reported positive outcomes, with no reports of harm.

Most interventions focused on providing emotional and psychological support for children and their parents. Sohamaran and Shorey [89] conducted a systematic review to evaluate the effectiveness of psychological interventions for parents of children with developmental disabilities in decreasing their stress, depression, and anxiety. Psychological interventions were found to decrease parental stress in the short term, but there was inconclusive evidence to demonstrate a reduction in parental depression and anxiety. This same systematic review included a variety of types of psychological interventions including CBT, mindfulness, psychoeducation, Stepping Stones Triple P (SSTP), parent education, and behavioural management training. These types of interventions are similar to those described in this current scoping review. Sohamaran and Shorey [89] found that interventions provided individually were more effective than those provided in a group setting. This is similar to how parents preferred individual therapy over group therapy in Hoare and Kerly [52] study.

In this scoping review, mindfulness‐based interventions were used to provide parents and their children with psychosocial support. Findings from a large systematic review and meta‐analysis demonstrated that mindfulness‐based interventions are effective at improving the mental health of parents of children with intellectual and developmental disabilities, including stress, anxiety, and depression [90]. Mindfulness‐based interventions were also helpful at improving parent–child relationships. Interventions targeting parents only and those lasting 8 weeks or more were most effective.

Some interventions in this scoping review were offered in a group setting where parents could develop a social community with one another, providing and receiving support from other parents. These studies were feasible and cost‐effective because healthcare professionals did not need to take a wage for intervention delivery. Sartore and colleagues [91] conducted a systematic review to assess the effects of peer support interventions on psychological and psychosocial outcomes of parents of children with complex needs. They found no difference between the intervention and control groups. However, qualitative data demonstrated that parents valued peer support and appreciated the emotional support. Wong and Shorey [92] conducted a qualitative systematic review to understand the experiences of parents of neurodivergent children receiving peer support. Parents sought peer support because of the inadequacy of formal support systems, and such support provided parents with emotional, appraisal, information, and instrumental support. Overall, Wong and Shorey [92] found that peer support was a valuable source of social support, enabling parents to provide quality care to their child and enhancing their quality of life.

CBT was a psychosocial care approach used in the included studies of this scoping review. CBT involves a variety of techniques that help individuals identify, challenge, and modify maladaptive thoughts and behaviours [93]. Interventions in this scoping review that utilized CBT involved promoting positive thinking, challenging existing thoughts, educating parents on using cognitive restructuring, and shifting parents' thinking to be more resource and solution‐focused. In a systematic review evaluating the effects of psychological interventions among parents of children with a chronic illness [94], CBT was found to significantly improve children with a chronic illness' symptoms and problem‐solving and significantly improve parental behaviour and mental health immediately after treatment.

In this scoping review, a majority of interventions were delivered in person; however, some interventions were delivered remotely, and one intervention used a web‐based design. Lappalainen and colleagues [95] conducted a randomized controlled trial to determine if their web‐based intervention, called the iACT, which involved three online meetings with a psychologist lasting 13 weeks, along with informational modules and assignments, was effective in reducing burnout, depression, quality of life, psychological flexibility, and mindfulness skills in parents of children with a chronic disease. The iACT intervention resulted in significant improvements in depressive symptoms, psychological flexibility, and mindfulness. Web‐based psychosocial support designs could be helpful for parents in the hospital setting because they lack the flexibility to leave their children's bedside to seek professional psychosocial help.

Although 39 of the 59 included studies were from the United States, United Kingdom, Australia, and Canada, the findings included multiple studies conducted in EU countries (e.g., Sweden, Switzerland, Iceland, Netherlands, and Denmark). These healthcare systems often provide comprehensive social support structures for families managing childhood chronic illness compared to other countries. This enhanced social support likely creates an optimal environment for implementing multimodal psychosocial support interventions since families can focus on therapeutic engagement without additional financial stressors. Several European studies included in this review demonstrate innovative approaches that could be adapted across European healthcare systems. For example, the comprehensive multidisciplinary psychosocial care model for children with haemophilia in the Netherlands [59] and the family therapeutic conversation intervention for families of children with a chronic illness in Iceland [74]. Integration of psychosocial support interventions within universal healthcare frameworks may achieve better outcomes. Future work should examine how psychosocial support interventions interface with increased social support structures found within EU countries.

### 4.1 Future Recommendations

According to de Brito Senna and colleagues [96], spirituality within healthcare is defined as a human characteristic that involves inner feelings and includes individuals' beliefs, values, practices, and experiences that help foster meaning. Spiritual approaches to providing psychosocial care were limited in number. Therefore, it is recommended that future studies include developing, implementing, and evaluating spiritual interventions among hospitalized children with a chronic disease and their parents. Since studies that occurred in the hospital setting were only included, it is recommended to replicate this scoping review for psychosocial interventions that are provided in the community, since many children with a chronic disease are cared for in the community.

There are key considerations from this scoping review that HCP and researchers should take into account when developing and implementing future psychosocial support interventions for children with a chronic disease admitted to hospital and their parents. Most interventions were multimodal; thus, interventions should target two or more psychosocial domains. Also, most interventions had positive outcomes in children's and parents' health and well‐being, disease management, family functioning, and hospital care experiences. Therefore, these outcomes should be considered in the development and implementation of future interventions. There was a variety in preferences between group and individual delivery models; therefore, if possible, children and parents should be offered a choice. To increase parents' ability to participate in the intervention, online or hybrid options should be provided. Finally, since most interventions included a psychological/emotional approach, and it is well known that children with a chronic disease and their parents struggle with their mental health when admitted to the hospital, psychological and emotional approaches should be incorporated.

### 4.2 Limitations

The studies were not appraised using a quality appraisal tool; therefore, study biases were not incorporated into the reporting of the findings. Only studies written in English were included; therefore, some relevant studies published in a language other than English could have been excluded. Grey literature and dissertations were not searched, which may have resulted in pertinent studies being missed. Interventions that took place in the hospital were only included in this review. Many children with a chronic disorder are cared for in the community; thus, valuable interventions and programs could have been excluded.

## 5 Conclusion

This scoping review examined the available evidence on psychosocial supports provided to children with a chronic disease admitted to hospital and their parents. The findings demonstrate that psychosocial interventions in hospital settings are predominantly multimodal in nature, incorporating multiple domains of care, including: emotional and psychological, informational, social, spiritual, and practical. Most supports demonstrated positive outcomes, with no reported adverse effects, highlighting the benefits of offering children and their parents psychosocial interventions in the hospital setting. Paediatric healthcare organizations should prioritize the development of infrastructure that supports the provision of psychosocial interventions for children with a chronic disease and their parents to improve children's and parents' outcomes during hospitalization, potentially reducing the long‐term psychological impacts of chronic illness management.

## Author Contributions

**Una Chang:** investigation, writing – original draft, validation, writing – review and editing, data curation. **K. Alix Hayden:** methodology, data curation, conceptualization, writing – original draft, validation, writing – review and editing. **Kathryn Birnie:** conceptualization, writing – review and editing, validation, methodology. **Lyndsay Jerusha MacKay:** conceptualization, investigation, funding acquisition, writing – original draft, methodology, validation, writing – review and editing, formal analysis, project administration, supervision, data curation.

## Conflicts of Interest

The authors declare no conflicts of interest.

## Supporting information


**Data S1:** apa70492‐sup‐0001‐supinfo.docx.

**Data S2:** apa70492‐sup‐0002‐supinfo.pdf.

## Data Availability

The data that support the findings of this study are available from the corresponding author upon reasonable request.
