# Supplementary material for: Supporting Children With a Chronic Disease and Their Parents When Admitted to Hospital: A Scoping Review of Psychosocial Supports
Source: Acta Paediatr. 2026 Mar 30;115(7):1322–72. doi: 10.1111/apa.70492 (PMC13250971; doi:10.1111/apa.70492)
Supplement: Supplementary file 1 — Data S1: apa70492‐sup‐0001‐supinfo.docx. [file APA-115-1322-s001.docx]

**Supplementary Material II**

**FINAL Searches**

**Psychosocial supports for children and their parents when admitted to hospital: A scoping review**

**Date: May 2 2024 -**

Database(s): **Ovid MEDLINE(R) ALL**1946 to May 01, 2024 
Search Strategy:

| **#** | **Searches** | **Results** |
| --- | --- | --- |
| 1 | Chronic Disease/ or Disabled Children/ | 291633 |
| 2 | exp Anemia/ or Anemia, Sickle Cell/ or arthritis/ or arthritis, juvenile/ or asthma/ or asthma, exercise-induced/ or autism spectrum disorder/ or asperger syndrome/ or autistic disorder/ or brain injuries/ or brain injuries, traumatic/ or brain injury, chronic/ or Brain Damage, Chronic/ or Cerebral Palsy/ or Congenital Abnormalities/ or Cystic Fibrosis/ or Developmental Disabilities/ or diabetes mellitus/ or diabetes mellitus, type 1/ | 839396 |
| 3 | Down Syndrome/ or "Chromosomes, Human, Pair 21"/ or Trisomy/ or exp Epilepsy/ or Heart Diseases/ or exp Heart Defects, Congenital/ or Genetic Diseases, Inborn/ or Hemophilia A/ or Hematologic Diseases/ or hiv/ or hiv-1/ or hiv-2/ or exp HIV Infections/ or Intellectual Disability/ | 871952 |
| 4 | exp Kidney Diseases/ or Meningomyelocele/ or exp Musculoskeletal Diseases/ or exp Spinal Dysraphism/ or Child Development Disorders, Pervasive/ or Celiac Disease/ or inflammatory bowel diseases/ or colitis, ulcerative/ or crohn disease/ or Complex Regional Pain Syndromes/ or exp Chromosome Aberrations/ or Craniofacial Abnormalities/ or Cleft Palate/ or Bronchopulmonary Dysplasia/ or Scoliosis/ or exp Skin Diseases/ or Eczema/ or sleep apnea syndromes/ or sleep apnea, central/ or sleep apnea, obstructive/ or Hydrocephalus/ | 3290876 |
| 5 | ((chronic* or critical or longterm or long-term) adj4 (disease* or disorder* or illness* or condition* or pain or syndrome*)).tw,kf. | 670049 |
| 6 | (life-limiting adj2 (disease* or disorder* or illness* or condition* or disabilit*)).tw,kf. | 2396 |
| 7 | ((special health care or special healthcare) adj3 (need* or condition* or disease* or disorder* or illness*)).tw,kf. | 2117 |
| 8 | Medically at-risk.tw,kf. | 79 |
| 9 | (complex adj4 (chronic condition* or health condition* or medical condition* or care need* or medical need* or healthcare need* or health care need* or health need* or disabilit*)).tw,kf. | 5008 |
| 10 | (medical* adj2 (complex* or fragile*)).tw,kf. | 7734 |
| 11 | (health adj2 (complex* or fragile*)).tw,kf. | 6265 |
| 12 | (Technolog* adj2 dependen*).tw,kf. | 855 |
| 13 | (ventilator adj2 (dependent* or assist*)).tw,kf. | 1861 |
| 14 | (mechanical* adj2 assist* adj2 ventilat*).tw,kf. | 534 |
| 15 | mechanically ventilated.tw,kf. | 14236 |
| 16 | medically fragile technology dependent*.tw,kf. | 4 |
| 17 | polyhandicap*.tw,kf. | 29 |
| 18 | (profound adj4 (intellectual or disabilit*)).tw,kf. | 1276 |
| 19 | ((intellectual* or brain* or cogniti* or mental) adj2 (deficit* or delay* or deviation* or disabil* or disabled or disorder* or dysfunction* or handicap* or impair* or retard*)).tw,kf. | 311076 |
| 20 | (deformit* or disabled or disabilit* or handicap*).tw,kf. | 380202 |
| 21 | ((mentally or intellectually or physically) adj1 challenged).tw,kf. | 257 |
| 22 | (Anemia or anaemia or Thalassemia).tw,kf. | 190721 |
| 23 | ((HbS or hemoglobin or haemoglobin or sickle cell or sickling) adj2 (condition* or disease* or disorder*)).tw,kf. | 22091 |
| 24 | (sickle cell adj2 anemia*).tw,kf. | 7957 |
| 25 | (arthriti* or periarthriti* or peri arthriti* or polyarthriti* or poly arthriti*).tw,kf. | 228850 |
| 26 | asthma*.tw,kf. | 187387 |
| 27 | (autis* or asperger* or kanner* syndrome*).tw,kf. | 72066 |
| 28 | (brain* adj3 (injur* or commotio* or damag* or trauma*)).tw,kf. | 132335 |
| 29 | (tbi or tbis or mtbi or concussion*).tw,kf. | 46848 |
| 30 | (cerebral palsy or (diplegia adj1 spastic) or Little* disease).tw,kf. | 29056 |
| 31 | ((brain* or central*) adj2 palsy).tw,kf. | 368 |
| 32 | ((brain or central* or cerebral*) adj2 (paralys* or paresis or pareses)).tw,kf. | 1117 |
| 33 | ((congenital* or birth) adj2 (abnormal* or anomal* or defect* or deform* or malform*)).tw,kf. | 98333 |
| 34 | cystic fibros*.tw,kf. | 52590 |
| 35 | ((fibrocystic or fibro-cystic) adj3 pancrea*).tw,kf. | 42 |
| 36 | mucoviscidos*.tw,kf. | 1551 |
| 37 | (development* adj2 (delay* or deviat* or disabilit* or disabled or disorder?)).tw,kf. | 57499 |
| 38 | ((autoimmune or brittle or insulin-dependent or juvenile) adj3 diabet*).tw,kf. | 35322 |
| 39 | (("Type 1" or "Type I" or ID) adj DM).tw,kf. | 1586 |
| 40 | (IDDM or T1D).tw,kf. | 18025 |
| 41 | (Down* adj2 syndrome*).tw,kf. | 25980 |
| 42 | (mongolism* or mongoloid*).tw,kf. | 2837 |
| 43 | ("trisomy 21" or "trisomy G1" or "trisomy (G)1" or "trisomy G-1" or "trisomy GM" or "trisomy G" or "21 trisomy" or "G1 trisomy" or "G(1) trisomy" or "G-1 trisomy" or "GM trisomy" or "G trisomy").tw,kf. | 7378 |
| 44 | ((chromosom* adj1 triplicat*) or trisom*).tw,kf. | 22328 |
| 45 | (translocat* adj2 DS).tw,kf. | 12 |
| 46 | (petit mal or grand mal or absence status).tw,kf. | 2631 |
| 47 | (epileps* or epilept* or seizure* or convulsi*).tw,kf. | 255481 |
| 48 | ((Dravet* or Landau-Kleffner* or Lennox Gastaut* or Doose* or Ohtahara* or Sturge-Weber* or West or "West's") adj2 syndrome*).tw,kf. | 6517 |
| 49 | ((sturge* or weber*) adj2 (disease* or syndrome*)).tw,kf. | 4014 |
| 50 | (myoclonic encephalopath* or action myoclonus-renal failure syndrome* or atypical inclusion-body disease* or biotin-responsive encephalopath* or haw river syndrome* or may white syndrome* or myoclonus-nephropathy syndrome* or naito oyanagi disease*).tw,kf. | 328 |
| 51 | SMEI.tw,kf. | 180 |
| 52 | (MERRF or fukuhara disease* or fukuhara disorder* or myoencephalopathy ragged-red fiber disease* or myoencephalopathy ragged-red fibre disease*).tw,kf. | 552 |
| 53 | Lafora.tw,kf. | 700 |
| 54 | ((Unverricht* adj1 Lundborg) or Baltic Myoclonus or Unverricht disease* or Unverricht* syndrome*).tw,kf. | 327 |
| 55 | ((infantile or nodding) adj2 spasm?).tw,kf. | 2950 |
| 56 | hypsarrhythmi*.tw,kf. | 973 |
| 57 | ((cardiac* or cardio* or heart*) adj2 (abnormalit* or anomal* or atypical* or a-typical* or defect* or deficien* or deform* or disorder? or impair* or malform*)).tw,kf. | 84241 |
| 58 | (tetralog* adj2 fallot*).tw,kf. | 11431 |
| 59 | ((cardiac* or cardio* or heart?) adj5 (congenital* or inborn* or hereditar* or inherit*)).tw,kf. | 70833 |
| 60 | ((genetic or hereditary or inherited or inborn) adj2 (condition* or disease* or disorder*)).tw,kf. | 99981 |
| 61 | single gene defect?.tw,kf. | 572 |
| 62 | (haemophili* or hemophili* or (("factor VIII" or "factor 8") adj3 deficien*)).tw,kf. | 28246 |
| 63 | ((blood or haematologic* or hematologic*) adj2 (condition* or disease* or disorder*)).tw,kf. | 38376 |
| 64 | (HIV-1 or HIV1 or HIV-I or HIVI or (HIV adj1 type 1) or (HIV adj1 type I)).tw,kf. | 88551 |
| 65 | (HIV-2 or HIV2 or HIV-II or HIVII or (HIV adj1 type 2) or (HIV adj1 type II)).tw,kf. | 5178 |
| 66 | (immunodeficiency virus* adj2 human*).tw,kf. | 100187 |
| 67 | (immuno-deficiency virus* adj2 human*).tw,kf. | 307 |
| 68 | ((acquired immunodeficiency or acquired immuno-deficiency) adj2 virus*).tw,kf. | 1337 |
| 69 | AIDS virus*.tw,kf. | 1133 |
| 70 | ((kidney* or renal) adj2 (condition* or disease* or disorder*)).tw,kf. | 202509 |
| 71 | (meningomyelocele or meningo-myelocele or myelocele).tw,kf. | 1178 |
| 72 | ((musculoskelet* or musculo-skelet* or muscle* or muscular or skelet* or orthop?edic*) adj2 (abnormalit* or deformit* or disorder* or disease*)).tw,kf. | 48271 |
| 73 | (MSD mor MSDs or MSKD or MSKDs).tw,kf. | 35 |
| 74 | ((spina* or status) adj (bifida* or dysraphi*)).tw,kf. | 10186 |
| 75 | (((cleft or open) adj spine*) or rachischis* or schistorrhach*).tw,kf. | 219 |
| 76 | (bowel* adj3 inflammatory adj3 (condition* or disease* or illness*)).tw,kf. | 69395 |
| 77 | (celiac or coeliac).tw,kf. | 32741 |
| 78 | pain.ti. | 235736 |
| 79 | (chromosomal adj2 (anomal* or abberation* or abnormalit*)).tw,kf. | 16656 |
| 80 | ((cranio facial or craniofacial) adj2 (abnormalit* or anomal*)).tw,kf. | 3795 |
| 81 | ((cleft or lip) adj2 palate).tw,kf. | 22915 |
| 82 | Bronchopulmonary Dysplasia.tw,kf. | 9653 |
| 83 | scoliosis.tw,kf. | 26578 |
| 84 | (skin adj2 (disease* or disorder*)).tw,kf. | 49794 |
| 85 | eczema.tw,kf. | 21212 |
| 86 | (sleep adj2 (apnea or disorder*)).tw,kf. | 68484 |
| 87 | hydrocephal*.tw,kf. | 32440 |
| 88 | or/1-87 | 6734931 |
| 89 | social support/ or community support/ or family support/ or psychosocial support systems/ | 81153 |
| 90 | psychotherapy/ or animal assisted therapy/ or aromatherapy/ or art therapy/ or behavior therapy/ or cognitive behavioral therapy/ or "acceptance and commitment therapy"/ or mindfulness/ or dialectical behavior therapy/ or relaxation therapy/ or meditation/ or imagery, psychotherapy/ or interpersonal psychotherapy/ or mentalization-based therapy/ or music therapy/ or narrative therapy/ or person-centered psychotherapy/ or play therapy/ or psychotherapy, brief/ or psychotherapy, multiple/ or psychotherapy, psychodynamic/ or psychotherapy, rational-emotive/ or socioenvironmental therapy/ | 145316 |
| 91 | mind-body therapies/ or biofeedback, psychology/ or breathing exercises/ or hypnosis/ or laughter therapy/ or meditation/ or mental healing/ or psychodrama/ or relaxation therapy/ or tai ji/ or therapeutic touch/ or yoga/ | 38704 |
| 92 | sensory art therapies/ or art therapy/ or color therapy/ or dance therapy/ or Adaptation, Psychological/ or Coping Skills/ or Psychosocial Intervention/ or self efficacy/ or Emotional Adjustment/ or counseling/ or directive counseling/ or distance counseling/ or "Quality of Life"/px or Stress, Psychological/pc, px or Anxiety/pc, px or Depression/pc, px or Spiritual Therapies/ or spirituality/ or pastoral care/ or financial stress/ or occupational stress/ or Information Seeking Behavior/ | 307816 |
| 93 | (social adj3 (support* or network* or system*)).tw,kf. | 104850 |
| 94 | (support adj3 (group* or system* or emotion* or physical)).tw,kf. | 66868 |
| 95 | ((psychosocial or psycho-social or biopsychosocial) adj3 (support* or intervention* or program* or care or outcome* or therap* or distress or problem* or need* or adjust* or wellbeing or well-being or wellness or burden* or factor*)).tw,kf. | 63766 |
| 96 | ((psycho* or behavior* or behaviour* or education* or group* or cogniti* or family) adj3 therapy).tw,kf. | 88741 |
| 97 | ((cope* or coping) adj3 (skill* or strateg* or intervention* or program* or behavio*)).tw,kf. | 33009 |
| 98 | ((psychologic* or emotion* or stress or distress or anxiety or depress*) adj3 (adjust* or adapt* or reduce or reduction or support* or well-being or wellbeing or wellness or burden* or factor*)).tw,kf. | 207930 |
| 99 | self-efficacy.tw,kf. | 43514 |
| 100 | (mental health adj3 (need* or care or support* or intervention* or program*)).tw,kf. | 43540 |
| 101 | (spiritual* or pastoral or religio*).tw,kf. | 73046 |
| 102 | ((financial or economic or occupation* or work or employ* or job*) adj3 (stress* or toxicity or burden* or insecurit*)).tw,kf. | 57809 |
| 103 | (information adj3 (need* or requirement* or health or request* or seek*)).tw,kf. | 89763 |
| 104 | or/89-103 | 1078465 |
| 105 | patients/ or adolescent, hospitalized/ or child, hospitalized/ or inpatients/ | 63940 |
| 106 | Hospitals/ or Hospitals, Pediatric/ or Hospital Units/ or hospitalization/ or patient admission/ | 286524 |
| 107 | Intensive Care Units, Pediatric/ or Intensive Care Units, Neonatal/ | 28341 |
| 108 | "continuity of patient care"/ or patient discharge/ or Transitional Care/ or Patient Readmission/ | 79280 |
| 109 | ((pediatric* or paediatric*) adj3 (room* or department* or facilit*)).tw,kf. | 18985 |
| 110 | (ward* or wards or unit or units).tw,kf. | 860383 |
| 111 | (PICU* or ICU or ICUS or NICU* or intensive care or critical care or hospital* or inpatient*).tw,kf. | 1935215 |
| 112 | (discharg* or postdischarg* or post-discharg* or readmission or re-admission or readmit* or re-admit*).tw,kf. | 384830 |
| 113 | (transition* adj3 home).tw,kf. | 1277 |
| 114 | (hospital adj3 home).tw,kf. | 9124 |
| 115 | ((transfer* or coordinat* or transitional) adj3 care).tw,kf. | 21891 |
| 116 | (home adj3 (care or healthcare)).tw,kf. | 40238 |
| 117 | or/105-116 | 2819503 |
| 118 | adolescent/ or child/ or child, preschool/ | 3429238 |
| 119 | infant/ or infant, newborn/ | 1270262 |
| 120 | (pediatric* or paediatric* or child*).tw,kf. | 1954529 |
| 121 | (boy or boys or girl* or juvenile* or teen* or tween* or preteen* or pre-teen* or youth* or adolesc* or prepubesc* or pubescen*or pre-pubesc*).tw,kf. | 787791 |
| 122 | (preschool* or pre-school* or school-age* or toddler* or infant* or baby or babies or newborn* or neonate*).tw,kf. | 814777 |
| 123 | or/118-122 | 4871587 |
| 124 | Psychosocial Intervention/ | 1138 |
| 125 | Health Education/ or pilot projects/ or program evaluation/ or Program Development/ | 293991 |
| 126 | (intervention* or program* or training or course* or education or project* or curriculum or model* or framework*).tw,kf. | 7613868 |
| 127 | or/124-126 | 7740739 |
| 128 | 88 and 104 and 117 and 123 and 127 | 7859 |
| 129 | limit 128 to yr="2000 -Current" | 6853 |

Database(s): **Embase**1974 to 2024 May 03 
Search Strategy:

| **#** | **Searches** | **Results** |
| --- | --- | --- |
| 1 | disabled child/ or handicapped child/ or chronic disease/ | 217783 |
| 2 | exp anemia/ or sickle cell anemia/ or arthritis/ or juvenile rheumatoid arthritis/ or systemic juvenile idiopathic arthritis/ or asthma/ or exercise induced asthma/ or autism/ or asperger syndrome/ or brain injury/ or head injury/ or acquired brain injury/ or brain concussion/ or brain damage/ or shaken baby syndrome/ or cerebral palsy/ or congenital disorder/ or cystic fibrosis/ or developmental disorder/ or diabetes mellitus/ or insulin dependent diabetes mellitus/ | 2167749 |
| 3 | Down syndrome/ or chromosome 21/ or trisomy/ or exp epilepsy/ or heart disease/ or congenital heart malformation/ or genetic disorder/ or hemophilia A/ or hematologic disease/ or exp Human immunodeficiency virus/ or exp Human immunodeficiency virus infection/ or intellectual impairment/ | 1481480 |
| 4 | exp kidney disease/ or meningomyelocele/ or exp musculoskeletal disease/ or exp spinal dysraphism/ or "pervasive developmental disorder not otherwise specified"/ or celiac disease/ or inflammatory bowel disease/ or crohn disease/ or ulcerative colitis/ or complex regional pain syndrome/ or exp chromosome aberration/ or craniofacial malformation/ or cleft palate/ or lung dysplasia/ or scoliosis/ or exp skin disease/ or eczema/ or sleep apnea syndromes/ or hydrocephalus/ | 5666609 |
| 5 | ((chronic* or critical or longterm or long-term) adj4 (disease* or disorder* or illness* or condition* or pain or syndrome*)).tw,kf. | 984075 |
| 6 | (life-limiting adj2 (disease* or disorder* or illness* or condition* or disabilit*)).tw,kf. | 3965 |
| 7 | ((special health care or special healthcare) adj3 (need* or condition* or disease* or disorder* or illness*)).tw,kf. | 2482 |
| 8 | Medically at-risk.tw,kf. | 87 |
| 9 | (complex adj4 (chronic condition* or health condition* or medical condition* or care need* or medical need* or healthcare need* or health care need* or health need* or disabilit*)).tw,kf. | 6827 |
| 10 | (medical* adj2 (complex* or fragile*)).tw,kf. | 12458 |
| 11 | (health adj2 (complex* or fragile*)).tw,kf. | 7485 |
| 12 | (Technolog* adj2 dependen*).tw,kf. | 1121 |
| 13 | (ventilator adj2 (dependent* or assist*)).tw,kf. | 2826 |
| 14 | (mechanical* adj2 assist* adj2 ventilat*).tw,kf. | 805 |
| 15 | mechanically ventilated.tw,kf. | 22140 |
| 16 | medically fragile technology dependent*.tw,kf. | 6 |
| 17 | polyhandicap*.tw,kf. | 68 |
| 18 | (profound adj4 (intellectual or disabilit*)).tw,kf. | 1779 |
| 19 | ((intellectual* or brain* or cogniti* or mental) adj2 (deficit* or delay* or deviation* or disabil* or disabled or disorder* or dysfunction* or handicap* or impair* or retard*)).tw,kf. | 428467 |
| 20 | (deformit* or disabled or disabilit* or handicap*).tw,kf. | 514371 |
| 21 | ((mentally or intellectually or physically) adj1 challenged).tw,kf. | 369 |
| 22 | (Anemia or anaemia or Thalassemia).tw,kf. | 281261 |
| 23 | ((HbS or hemoglobin or haemoglobin or sickle cell or sickling) adj2 (condition* or disease* or disorder*)).tw,kf. | 35204 |
| 24 | (sickle cell adj2 anemia*).tw,kf. | 9611 |
| 25 | (arthriti* or periarthriti* or peri arthriti* or polyarthriti* or poly arthriti*).tw,kf. | 332002 |
| 26 | asthma*.tw,kf. | 274860 |
| 27 | (autis* or asperger* or kanner* syndrome*).tw,kf. | 92999 |
| 28 | (brain* adj3 (injur* or commotio* or damag* or trauma*)).tw,kf. | 179491 |
| 29 | (tbi or tbis or mtbi or concussion*).tw,kf. | 72274 |
| 30 | (cerebral palsy or (diplegia adj1 spastic) or Little* disease).tw,kf. | 40897 |
| 31 | ((brain* or central*) adj2 palsy).tw,kf. | 676 |
| 32 | ((brain or central* or cerebral*) adj2 (paralys* or paresis or pareses)).tw,kf. | 1159 |
| 33 | ((congenital* or birth) adj2 (abnormal* or anomal* or defect* or deform* or malform*)).tw,kf. | 125003 |
| 34 | cystic fibros*.tw,kf. | 81928 |
| 35 | ((fibrocystic or fibro-cystic) adj3 pancrea*).tw,kf. | 9 |
| 36 | mucoviscidos*.tw,kf. | 1127 |
| 37 | (development* adj2 (delay* or deviat* or disabilit* or disabled or disorder?)).tw,kf. | 81199 |
| 38 | ((autoimmune or brittle or insulin-dependent or juvenile) adj3 diabet*).tw,kf. | 43631 |
| 39 | (("Type 1" or "Type I" or ID) adj DM).tw,kf. | 3239 |
| 40 | (IDDM or T1D).tw,kf. | 31134 |
| 41 | (Down* adj2 syndrome*).tw,kf. | 33457 |
| 42 | (mongolism* or mongoloid*).tw,kf. | 1318 |
| 43 | ("trisomy 21" or "trisomy G1" or "trisomy (G)1" or "trisomy G-1" or "trisomy GM" or "trisomy G" or "21 trisomy" or "G1 trisomy" or "G(1) trisomy" or "G-1 trisomy" or "GM trisomy" or "G trisomy").tw,kf. | 10766 |
| 44 | ((chromosom* adj1 triplicat*) or trisom*).tw,kf. | 31155 |
| 45 | (translocat* adj2 DS).tw,kf. | 16 |
| 46 | (petit mal or grand mal or absence status).tw,kf. | 3053 |
| 47 | (epileps* or epilept* or seizure* or convulsi*).tw,kf. | 360168 |
| 48 | ((Dravet* or Landau-Kleffner* or Lennox Gastaut* or Doose* or Ohtahara* or Sturge-Weber* or West or "West's") adj2 syndrome*).tw,kf. | 9987 |
| 49 | ((sturge* or weber*) adj2 (disease* or syndrome*)).tw,kf. | 4641 |
| 50 | (myoclonic encephalopath* or action myoclonus-renal failure syndrome* or atypical inclusion-body disease* or biotin-responsive encephalopath* or haw river syndrome* or may white syndrome* or myoclonus-nephropathy syndrome* or naito oyanagi disease*).tw,kf. | 457 |
| 51 | SMEI.tw,kf. | 301 |
| 52 | (MERRF or fukuhara disease* or fukuhara disorder* or myoencephalopathy ragged-red fiber disease* or myoencephalopathy ragged-red fibre disease*).tw,kf. | 764 |
| 53 | Lafora.tw,kf. | 919 |
| 54 | ((Unverricht* adj1 Lundborg) or Baltic Myoclonus or Unverricht disease* or Unverricht* syndrome*).tw,kf. | 433 |
| 55 | ((infantile or nodding) adj2 spasm?).tw,kf. | 4432 |
| 56 | hypsarrhythmi*.tw,kf. | 1373 |
| 57 | ((cardiac* or cardio* or heart*) adj2 (abnormalit* or anomal* or atypical* or a-typical* or defect* or deficien* or deform* or disorder? or impair* or malform*)).tw,kf. | 111754 |
| 58 | (tetralog* adj2 fallot*).tw,kf. | 14936 |
| 59 | ((cardiac* or cardio* or heart?) adj5 (congenital* or inborn* or hereditar* or inherit*)).tw,kf. | 98555 |
| 60 | ((genetic or hereditary or inherited or inborn) adj2 (condition* or disease* or disorder*)).tw,kf. | 145557 |
| 61 | single gene defect?.tw,kf. | 840 |
| 62 | (haemophili* or hemophili* or (("factor VIII" or "factor 8") adj3 deficien*)).tw,kf. | 44850 |
| 63 | ((blood or haematologic* or hematologic*) adj2 (condition* or disease* or disorder*)).tw,kf. | 52603 |
| 64 | (HIV-1 or HIV1 or HIV-I or HIVI or (HIV adj1 type 1) or (HIV adj1 type I)).tw,kf. | 109218 |
| 65 | (HIV-2 or HIV2 or HIV-II or HIVII or (HIV adj1 type 2) or (HIV adj1 type II)).tw,kf. | 6387 |
| 66 | (immunodeficiency virus* adj2 human*).tw,kf. | 112528 |
| 67 | (immuno-deficiency virus* adj2 human*).tw,kf. | 399 |
| 68 | ((acquired immunodeficiency or acquired immuno-deficiency) adj2 virus*).tw,kf. | 1477 |
| 69 | AIDS virus*.tw,kf. | 1162 |
| 70 | ((kidney* or renal) adj2 (condition* or disease* or disorder*)).tw,kf. | 306566 |
| 71 | (meningomyelocele or meningo-myelocele or myelocele).tw,kf. | 1580 |
| 72 | ((musculoskelet* or musculo-skelet* or muscle* or muscular or skelet* or orthop?edic*) adj2 (abnormalit* or deformit* or disorder* or disease*)).tw,kf. | 64517 |
| 73 | (MSD mor MSDs or MSKD or MSKDs).tw,kf. | 56 |
| 74 | ((spina* or status) adj (bifida* or dysraphi*)).tw,kf. | 13102 |
| 75 | (((cleft or open) adj spine*) or rachischis* or schistorrhach*).tw,kf. | 255 |
| 76 | (bowel* adj3 inflammatory adj3 (condition* or disease* or illness*)).tw,kf. | 116934 |
| 77 | (celiac or coeliac).tw,kf. | 48635 |
| 78 | pain.ti. | 307896 |
| 79 | (chromosomal adj2 (anomal* or abberation* or abnormalit*)).tw,kf. | 25186 |
| 80 | ((cranio facial or craniofacial) adj2 (abnormalit* or anomal*)).tw,kf. | 5003 |
| 81 | ((cleft or lip) adj2 palate).tw,kf. | 25415 |
| 82 | Bronchopulmonary Dysplasia.tw,kf. | 13631 |
| 83 | scoliosis.tw,kf. | 34798 |
| 84 | (skin adj2 (disease* or disorder*)).tw,kf. | 64531 |
| 85 | eczema.tw,kf. | 30232 |
| 86 | (sleep adj2 (apnea or disorder*)).tw,kf. | 113895 |
| 87 | hydrocephal*.tw,kf. | 42704 |
| 88 | or/1-87 | 10275786 |
| 89 | social support/ or community support/ or emotional support/ or family support/ | 124226 |
| 90 | psychotherapy/ or art therapy/ or group therapy/ or mindfulness/ or play therapy/ or relaxation training/ or solution-focused therapy/ or animal assisted therapy/ or behavior therapy/ or cognitive behavioral therapy/ or mindfulness-based cognitive therapy/ or mindfulness-based stress reduction/ or problem solving therapy/ or trauma-focused cognitive behavioral therapy/ or "acceptance and commitment therapy"/ or cognitive therapy/ or dialectical behavior therapy/ or meditation/ or mindfulness meditation/ or guided imagery/ or interpersonal psychotherapy/ or mentalization-based treatment/ or music therapy/ or narrative therapy/ or client centered therapy/ or short term psychotherapy/ or psychodynamic psychotherapy/ | 249368 |
| 91 | alternative medicine/ or breathwork/ or laughter therapy/ or hypnosis/ or psychodrama/ or relaxation training/ or Tai Chi/ or yoga/ | 89918 |
| 92 | emotion regulation training/ or emotion-focused therapy/ or psychosocial intervention/ or color therapy/ or psychological adjustment/ or coping behavior/ or self concept/ or counseling/ or directive counseling/ or motivational interviewing/ or parent counseling/ or psychological counseling/ or anxiety/pc, th or mental stress/pc, th or depression/pc, th or spiritual healing/ or religion/ or pastoral care/ or financial stress/ or financial distress/ or job stress/ or information seeking/ | 411782 |
| 93 | (social adj3 (support* or network* or system*)).tw,kf. | 125679 |
| 94 | (support adj3 (group* or system* or emotion* or physical)).tw,kf. | 87673 |
| 95 | ((psychosocial or psycho-social or biopsychosocial) adj3 (support* or intervention* or program* or care or outcome* or therap* or distress or problem* or need* or adjust* or wellbeing or well-being or wellness or burden* or factor*)).tw,kf. | 88726 |
| 96 | ((psycho* or behavior* or behaviour* or education* or group* or cogniti* or family) adj3 therapy).tw,kf. | 129927 |
| 97 | ((cope* or coping) adj3 (skill* or strateg* or intervention* or program* or behavio*)).tw,kf. | 41958 |
| 98 | ((psychologic* or emotion* or stress or distress or anxiety or depress*) adj3 (adjust* or adapt* or reduce or reduction or support* or well-being or wellbeing or wellness or burden* or factor*)).tw,kf. | 261553 |
| 99 | self-efficacy.tw,kf. | 50732 |
| 100 | (mental health adj3 (need* or care or support* or intervention* or program*)).tw,kf. | 51628 |
| 101 | (spiritual* or pastoral or religio*).tw,kf. | 84261 |
| 102 | ((financial or economic or occupation* or work or employ* or job*) adj3 (stress* or toxicity or burden* or insecurit*)).tw,kf. | 81108 |
| 103 | (information adj3 (need* or requirement* or health or request* or seek*)).tw,kf. | 112090 |
| 104 | or/89-103 | 1425493 |
| 105 | hospitalized adolescent/ or hospital patient/ or hospitalized child/ or hospitalized infant/ | 246744 |
| 106 | hospital/ or pediatric hospital/ or hospitalization/ or hospital patient/ | 1143729 |
| 107 | neonatal intensive care unit/ or newborn intensive care/ or pediatric intensive care unit/ | 67795 |
| 108 | hospital discharge/ or hospital to home transition/ or transitional care/ or hospital readmission/ | 288842 |
| 109 | ((pediatric* or paediatric*) adj3 (room* or department* or facilit*)).tw,kf. | 32247 |
| 110 | (ward* or wards or unit or units).tw,kf. | 1174231 |
| 111 | (PICU* or ICU or ICUS or NICU* or intensive care or critical care or hospital* or inpatient*).tw,kf. | 2999725 |
| 112 | (discharg* or postdischarg* or post-discharg* or readmission or re-admission or readmit* or re-admit*).tw,kf. | 629703 |
| 113 | (transition* adj3 home).tw,kf. | 1934 |
| 114 | (hospital adj3 home).tw,kf. | 13425 |
| 115 | ((transfer* or coordinat* or transitional) adj3 care).tw,kf. | 34432 |
| 116 | (home adj3 (care or healthcare)).tw,kf. | 51102 |
| 117 | or/105-116 | 4343395 |
| 118 | adolescent/ | 1835088 |
| 119 | child/ or boy/ or girl/ or preschool child/ or school child/ or toddler/ | 2474752 |
| 120 | infant/ or baby/ or high risk infant/ or newborn/ | 1168283 |
| 121 | (pediatric* or paediatric* or child*).tw,kf. | 2508313 |
| 122 | (boy or boys or girl* or juvenile* or teen* or tween* or preteen* or pre-teen* or youth* or adolesc* or prepubesc* or pubescen*or pre-pubesc*).tw,kf. | 1007968 |
| 123 | (preschool* or pre-school* or school-age* or toddler* or infant* or baby or babies or newborn* or neonate*).tw,kf. | 943994 |
| 124 | or/118-123 | 5086396 |
| 125 | psychosocial intervention/ | 2570 |
| 126 | health education/ or parenting education/ | 114638 |
| 127 | program development/ | 26427 |
| 128 | (intervention* or program* or training or course* or education or project* or curriculum or model* or framework*).tw,kf. | 9788014 |
| 129 | or/125-128 | 9835019 |
| 130 | 88 and 104 and 117 and 124 and 129 | 14015 |
| 131 | limit 130 to (books or chapter or conference abstract or letter or note) | 5463 |
| 132 | 130 not 131 | 8552 |
| 133 | limit 132 to yr="2000 -Current" | 7845 |

Database(s): **APA PsycInfo**1806 to April Week 5 2024 
Search Strategy:

| **#** | **Searches** | **Results** |
| --- | --- | --- |
| 1 | chronic illness/ or chronically ill children/ or "chronicity (disorders)"/ or critical illness/ | 19701 |
| 2 | disabilities/ or multiple disabilities/ | 25735 |
| 3 | anemia/ or "blood and lymphatic disorders"/ or sickle cell disease/ or arthritis/ or asthma/ or autism spectrum disorders/ or neurodevelopmental disorders/ or developmental disabilities/ or brain damage/ or head injuries/ or brain injuries/ or cognitive impairment/ or cerebral palsy/ or congenital disorders/ or cystic fibrosis/ or diabetes/ or diabetes mellitus/ | 180972 |
| 4 | down's syndrome/ or trisomy/ or exp epilepsy/ or heart disorders/ or congenital disorders/ or genetic disorders/ or hemophilia/ or hiv/ | 99779 |
| 5 | kidney diseases/ or spina bifida/ or exp musculoskeletal disorders/ or celiac disease/ or colon disorders/ or colitis/ or cleft palate/ or skin disorders/ or eczema/ or sleep apnea/ or hydrocephalus/ | 35609 |
| 6 | ((chronic* or critical or longterm or long-term) adj4 (disease* or disorder* or illness* or condition* or pain or syndrome*)).ti,ab,id. | 95648 |
| 7 | (life-limiting adj2 (disease* or disorder* or illness* or condition* or disabilit*)).ti,ab,id. | 873 |
| 8 | ((special health care or special healthcare) adj3 (need* or condition* or disease* or disorder* or illness*)).ti,ab,id. | 859 |
| 9 | Medically at-risk.ti,ab,id. | 55 |
| 10 | (complex adj4 (chronic condition* or health condition* or medical condition* or care need* or medical need* or healthcare need* or health care need* or health need* or disabilit*)).ti,ab,id. | 2041 |
| 11 | (medical* adj2 (complex* or fragile*)).ti,ab,id. | 1543 |
| 12 | (health adj2 (complex* or fragile*)).ti,ab,id. | 2226 |
| 13 | (Technolog* adj2 dependen*).ti,ab,id. | 312 |
| 14 | (ventilator adj2 (dependent* or assist*)).ti,ab,id. | 82 |
| 15 | (mechanical* adj2 assist* adj2 ventilat*).ti,ab,id. | 4 |
| 16 | mechanically ventilated.ti,ab,id. | 283 |
| 17 | medically fragile technology dependent*.ti,ab,id. | 2 |
| 18 | polyhandicap*.ti,ab,id. | 26 |
| 19 | (profound adj4 (intellectual or disabilit*)).ti,ab,id. | 1377 |
| 20 | ((intellectual* or brain* or cogniti* or mental) adj2 (deficit* or delay* or deviation* or disabil* or disabled or disorder* or dysfunction* or handicap* or impair* or retard*)).ti,ab,id. | 219242 |
| 21 | (deformit* or disabled or disabilit* or handicap*).ti,ab,id. | 178480 |
| 22 | ((mentally or intellectually or physically) adj1 challenged).ti,ab,id. | 178 |
| 23 | (Anemia or anaemia or Thalassemia).ti,ab,id. | 2586 |
| 24 | ((HbS or hemoglobin or haemoglobin or sickle cell or sickling) adj2 (condition* or disease* or disorder*)).ti,ab,id. | 1498 |
| 25 | (sickle cell adj2 anemia*).ti,ab,id. | 290 |
| 26 | (arthriti* or periarthriti* or peri arthriti* or polyarthriti* or poly arthriti*).ti,ab,id. | 6225 |
| 27 | asthma*.ti,ab,id. | 8841 |
| 28 | (autis* or asperger* or kanner* syndrome*).ti,ab,id. | 69866 |
| 29 | (brain* adj3 (injur* or commotio* or damag* or trauma*)).ti,ab,id. | 49408 |
| 30 | (tbi or tbis or mtbi or concussion*).ti,ab,id. | 18531 |
| 31 | (cerebral palsy or (diplegia adj1 spastic) or Little* disease).ti,ab,id. | 8764 |
| 32 | ((brain* or central*) adj2 palsy).ti,ab,id. | 64 |
| 33 | ((brain or central* or cerebral*) adj2 (paralys* or paresis or pareses)).ti,ab,id. | 157 |
| 34 | ((congenital* or birth) adj2 (abnormal* or anomal* or defect* or deform* or malform*)).ti,ab,id. | 2985 |
| 35 | cystic fibros*.ti,ab,id. | 1357 |
| 36 | ((fibrocystic or fibro-cystic) adj3 pancrea*).ti,ab,id. | 0 |
| 37 | mucoviscidos*.ti,ab,id. | 4 |
| 38 | (development* adj2 (delay* or deviat* or disabilit* or disabled or disorder?)).ti,ab,id. | 34130 |
| 39 | ((autoimmune or brittle or insulin-dependent or juvenile) adj3 diabet*).ti,ab,id. | 1259 |
| 40 | (("Type 1" or "Type I" or ID) adj DM).ti,ab,id. | 59 |
| 41 | (IDDM or T1D).ti,ab,id. | 781 |
| 42 | (Down* adj2 syndrome*).ti,ab,id. | 8563 |
| 43 | (mongolism* or mongoloid*).ti,ab,id. | 679 |
| 44 | ("trisomy 21" or "trisomy G1" or "trisomy (G)1" or "trisomy G-1" or "trisomy GM" or "trisomy G" or "21 trisomy" or "G1 trisomy" or "G(1) trisomy" or "G-1 trisomy" or "GM trisomy" or "G trisomy").ti,ab,id. | 533 |
| 45 | ((chromosom* adj1 triplicat*) or trisom*).ti,ab,id. | 991 |
| 46 | (translocat* adj2 DS).ti,ab,id. | 1 |
| 47 | (petit mal or grand mal or absence status).ti,ab,id. | 728 |
| 48 | (epileps* or epilept* or seizure* or convulsi*).ti,ab,id. | 61957 |
| 49 | ((Dravet* or Landau-Kleffner* or Lennox Gastaut* or Doose* or Ohtahara* or Sturge-Weber* or West or "West's") adj2 syndrome*).ti,ab,id. | 1324 |
| 50 | ((sturge* or weber*) adj2 (disease* or syndrome*)).ti,ab,id. | 148 |
| 51 | (myoclonic encephalopath* or action myoclonus-renal failure syndrome* or atypical inclusion-body disease* or biotin-responsive encephalopath* or haw river syndrome* or may white syndrome* or myoclonus-nephropathy syndrome* or naito oyanagi disease*).ti,ab,id. | 40 |
| 52 | SMEI.ti,ab,id. | 39 |
| 53 | (MERRF or fukuhara disease* or fukuhara disorder* or myoencephalopathy ragged-red fiber disease* or myoencephalopathy ragged-red fibre disease*).ti,ab,id. | 47 |
| 54 | Lafora.ti,ab,id. | 98 |
| 55 | ((Unverricht* adj1 Lundborg) or Baltic Myoclonus or Unverricht disease* or Unverricht* syndrome*).ti,ab,id. | 45 |
| 56 | ((infantile or nodding) adj2 spasm?).ti,ab,id. | 508 |
| 57 | hypsarrhythmi*.ti,ab,id. | 138 |
| 58 | ((cardiac* or cardio* or heart*) adj2 (abnormalit* or anomal* or atypical* or a-typical* or defect* or deficien* or deform* or disorder? or impair* or malform*)).ti,ab,id. | 3416 |
| 59 | (tetralog* adj2 fallot*).ti,ab,id. | 57 |
| 60 | ((cardiac* or cardio* or heart?) adj5 (congenital* or inborn* or hereditar* or inherit*)).ti,ab,id. | 1306 |
| 61 | ((genetic or hereditary or inherited or inborn) adj2 (condition* or disease* or disorder*)).ti,ab,id. | 7775 |
| 62 | single gene defect?.ti,ab,id. | 18 |
| 63 | (haemophili* or hemophili* or (("factor VIII" or "factor 8") adj3 deficien*)).ti,ab,id. | 582 |
| 64 | ((blood or haematologic* or hematologic*) adj2 (condition* or disease* or disorder*)).ti,ab,id. | 1216 |
| 65 | (HIV-1 or HIV1 or HIV-I or HIVI or (HIV adj1 type 1) or (HIV adj1 type I)).ti,ab,id. | 2359 |
| 66 | (HIV-2 or HIV2 or HIV-II or HIVII or (HIV adj1 type 2) or (HIV adj1 type II)).ti,ab,id. | 117 |
| 67 | (immunodeficiency virus* adj2 human*).ti,ab,id. | 6987 |
| 68 | (immuno-deficiency virus* adj2 human*).ti,ab,id. | 16 |
| 69 | ((acquired immunodeficiency or acquired immuno-deficiency) adj2 virus*).ti,ab,id. | 178 |
| 70 | AIDS virus*.ti,ab,id. | 155 |
| 71 | ((kidney* or renal) adj2 (condition* or disease* or disorder*)).ti,ab,id. | 3689 |
| 72 | (meningomyelocele or meningo-myelocele or myelocele).ti,ab,id. | 77 |
| 73 | ((musculoskelet* or musculo-skelet* or muscle* or muscular or skelet* or orthop?edic*) adj2 (abnormalit* or deformit* or disorder* or disease*)).ti,ab,id. | 3655 |
| 74 | (MSD mor MSDs or MSKD or MSKDs).ti,ab,id. | 1 |
| 75 | ((spina* or status) adj (bifida* or dysraphi*)).ti,ab,id. | 1167 |
| 76 | (((cleft or open) adj spine*) or rachischis* or schistorrhach*).ti,ab,id. | 5 |
| 77 | (bowel* adj3 inflammatory adj3 (condition* or disease* or illness*)).ti,ab,id. | 1260 |
| 78 | (celiac or coeliac).ti,ab,id. | 631 |
| 79 | pain.ti. | 48619 |
| 80 | (chromosomal adj2 (anomal* or abberation* or abnormalit*)).ti,ab,id. | 485 |
| 81 | ((cranio facial or craniofacial) adj2 (abnormalit* or anomal*)).ti,ab,id. | 185 |
| 82 | ((cleft or lip) adj2 palate).ti,ab,id. | 818 |
| 83 | Bronchopulmonary Dysplasia.ti,ab,id. | 184 |
| 84 | scoliosis.ti,ab,id. | 484 |
| 85 | (skin adj2 (disease* or disorder*)).ti,ab,id. | 1179 |
| 86 | eczema.ti,ab,id. | 466 |
| 87 | (sleep adj2 (apnea or disorder*)).ti,ab,id. | 15546 |
| 88 | hydrocephal*.ti,ab,id. | 2216 |
| 89 | or/1-88 | 773449 |
| 90 | social support/ or emotional support/ or family relations/ or social connectedness/ or support groups/ | 89580 |
| 91 | psychosocial factors/ or psychosocial assessment/ or social influences/ | 55422 |
| 92 | exp psychotherapy/ or animal assisted therapy/ or aromatherapy/ or art therapy/ or creative arts therapy/ or movement therapy/ or exp behavior therapy/ or cognitive behavior therapy/ or "acceptance and commitment therapy"/ or cognitive therapy/ or mindfulness-based cognitive therapy/ or "Acceptance and Commitment Therapy"/ or mindfulness/ or mindfulness-based cognitive therapy/ or mindfulness-based interventions/ or mindfulness-based stress reduction/ or dialectical behavior therapy/ or relaxation therapy/ or mind body therapy/ or meditation/ or progressive relaxation therapy/ or guided imagery/ or hypnotherapy/ or interpersonal psychotherapy/ or brief psychotherapy/ or Milieu Therapy/ or psychodrama/ or yoga/ or dance therapy/ | 334277 |
| 93 | emotional adjustment/ or adjustment/ or coping behavior/ or psychosocial interventions/ or psychoeducation/ or psychosocial rehabilitation/ or self-efficacy/ or exp counseling/ or "stress and coping measures"/ or spirituality/ or spiritual well being/ or "religion and spirituality measures"/ or religiosity/ or spiritual care/ or spiritually oriented therapy/ or Pastoral Counseling/ or financial strain/ or job loss/ or occupational stress/ or information seeking/ | 271130 |
| 94 | (social adj3 (support* or network* or system*)).ti,ab,id. | 120971 |
| 95 | (support adj3 (group* or system* or emotion* or physical)).ti,ab,id. | 46547 |
| 96 | ((psychosocial or psycho-social or biopsychosocial) adj3 (support* or intervention* or program* or care or outcome* or therap* or distress or problem* or need* or adjust* or wellbeing or well-being or wellness or burden* or factor*)).ti,ab,id. | 49671 |
| 97 | ((psycho* or behavior* or behaviour* or education* or group* or cogniti* or family) adj3 therapy).ti,ab,id. | 105020 |
| 98 | ((cope* or coping) adj3 (skill* or strateg* or intervention* or program* or behavio*)).ti,ab,id. | 45449 |
| 99 | ((psychologic* or emotion* or stress or distress or anxiety or depress*) adj3 (adjust* or adapt* or reduce or reduction or support* or well-being or wellbeing or wellness or burden* or factor*)).ti,ab,id. | 164183 |
| 100 | self-efficacy.ti,ab,id. | 54283 |
| 101 | (mental health adj3 (need* or care or support* or intervention* or program*)).ti,ab,id. | 48338 |
| 102 | (spiritual* or pastoral or religio*).ti,ab,id. | 117319 |
| 103 | ((financial or economic or occupation* or work or employ* or job*) adj3 (stress* or toxicity or burden* or insecurit*)).ti,ab,id. | 28046 |
| 104 | (information adj3 (need* or requirement* or health or request* or seek*)).ti,ab,id. | 31539 |
| 105 | or/90-104 | 1050779 |
| 106 | hospitalized patients/ or patients/ or hospitalization/ or neonatal intensive care/ or hospitals/ or hospital discharge/ or client transfer/ or hospital admission/ | 75205 |
| 107 | ((pediatric* or paediatric*) adj3 (room* or department* or facilit*)).ti,ab,id. | 1285 |
| 108 | (ward* or wards or unit or units).ti,ab,id. | 116470 |
| 109 | (PICU* or ICU or ICUS or NICU* or intensive care or critical care or hospital* or inpatient*).ti,ab,id. | 231049 |
| 110 | (discharg* or postdischarg* or post-discharg* or readmission or re-admission or readmit* or re-admit*).ti,ab,id. | 45199 |
| 111 | (transition* adj3 home).ti,ab,id. | 783 |
| 112 | (hospital adj3 home).ti,ab,id. | 1896 |
| 113 | ((transfer* or coordinat* or transitional) adj3 care).ti,ab,id. | 5475 |
| 114 | (home adj3 (care or healthcare)).ti,ab,id. | 13665 |
| 115 | or/106-114 | 368669 |
| 116 | pediatrics/ | 33132 |
| 117 | elementary school students/ or intermediate school students/ or primary school students/ or middle school students/ or high school students/ | 92161 |
| 118 | (adolescence 13 17 yrs or childhood birth 12 yrs or infancy 2 23 mo or neonatal birth 1 mo or preschool age 2 5 yrs or school age 6 12 yrs).ag. | 912195 |
| 119 | (pediatric* or paediatric* or child*).ti,ab,id. | 828764 |
| 120 | (boy or boys or girl* or juvenile* or teen* or tween* or preteen* or pre-teen* or youth* or adolesc* or prepubesc* or pubescen*or pre-pubesc*).ti,ab,id. | 478373 |
| 121 | (preschool* or pre-school* or school-age* or toddler* or infant* or baby or babies or newborn* or neonate*).ti,ab,id. | 190612 |
| 122 | or/116-121 | 1389673 |
| 123 | psychosocial interventions/ or intervention/ or health education/ or mental health education/ or program evaluation/ or evaluation/ or educational program evaluation/ or mental health program evaluation/ or program development/ or educational program planning/ or hospital programs/ | 159536 |
| 124 | (intervention* or program* or training or course* or education or project* or curriculum or model* or framework*).ti,ab,id. | 2320179 |
| 125 | or/123-124 | 2334869 |
| 126 | 89 and 105 and 115 and 122 and 125 | 2936 |
| 127 | limit 126 to (bibliography or chapter or obituary or poetry or review-book or review-media or review-software & other) | 279 |
| 128 | 126 not 127 | 2657 |

Database(s): **JBI EBP Database**Current to May 01, 2024 
Search Strategy:

| **#** | **Searches** | **Results** |
| --- | --- | --- |
| 1 | ((chronic* or critical or longterm or long-term) adj4 (disease* or disorder* or illness* or condition* or pain or syndrome*)).ti,ab,kw. | 392 |
| 2 | (life-limiting adj2 (disease* or disorder* or illness* or condition* or disabilit*)).ti,ab,kw. | 6 |
| 3 | ((special health care or special healthcare) adj3 (need* or condition* or disease* or disorder* or illness*)).ti,ab,kw. | 2 |
| 4 | Medically at-risk.ti,ab,kw. | 0 |
| 5 | (complex adj4 (chronic condition* or health condition* or medical condition* or care need* or medical need* or healthcare need* or health care need* or health need* or disabilit*)).ti,ab,kw. | 11 |
| 6 | (medical* adj2 (complex* or fragile*)).ti,ab,kw. | 6 |
| 7 | (health adj2 (complex* or fragile*)).ti,ab,kw. | 12 |
| 8 | (Technolog* adj2 dependen*).ti,ab,kw. | 4 |
| 9 | (ventilator adj2 (dependent* or assist*)).ti,ab,kw. | 4 |
| 10 | (mechanical* adj2 assist* adj2 ventilat*).ti,ab,kw. | 2 |
| 11 | mechanically ventilated.ti,ab,kw. | 15 |
| 12 | medically fragile technology dependent*.ti,ab,kw. | 0 |
| 13 | polyhandicap*.ti,ab,kw. | 0 |
| 14 | (profound adj4 (intellectual or disabilit*)).ti,ab,kw. | 1 |
| 15 | ((intellectual* or brain* or cogniti* or mental) adj2 (deficit* or delay* or deviation* or disabil* or disabled or disorder* or dysfunction* or handicap* or impair* or retard*)).ti,ab,kw. | 119 |
| 16 | (deformit* or disabled or disabilit* or handicap*).ti,ab,kw. | 181 |
| 17 | ((mentally or intellectually or physically) adj1 challenged).ti,ab,kw. | 0 |
| 18 | (Anemia or anaemia or Thalassemia).ti,ab,kw. | 22 |
| 19 | ((HbS or hemoglobin or haemoglobin or sickle cell or sickling) adj2 (condition* or disease* or disorder*)).ti,ab,kw. | 11 |
| 20 | (sickle cell adj2 anemia*).ti,ab,kw. | 2 |
| 21 | (arthriti* or periarthriti* or peri arthriti* or polyarthriti* or poly arthriti*).ti,ab,kw. | 70 |
| 22 | asthma*.ti,ab,kw. | 71 |
| 23 | (autis* or asperger* or kanner* syndrome*).ti,ab,kw. | 25 |
| 24 | (brain* adj3 (injur* or commotio* or damag* or trauma*)).ti,ab,kw. | 57 |
| 25 | (tbi or tbis or mtbi or concussion*).ti,ab,kw. | 14 |
| 26 | (cerebral palsy or (diplegia adj1 spastic) or Little* disease).ti,ab,kw. | 25 |
| 27 | ((brain* or central*) adj2 palsy).ti,ab,kw. | 0 |
| 28 | ((brain or central* or cerebral*) adj2 (paralys* or paresis or pareses)).ti,ab,kw. | 0 |
| 29 | ((congenital* or birth) adj2 (abnormal* or anomal* or defect* or deform* or malform*)).ti,ab,kw. | 13 |
| 30 | cystic fibros*.ti,ab,kw. | 14 |
| 31 | ((fibrocystic or fibro-cystic) adj3 pancrea*).ti,ab,kw. | 0 |
| 32 | mucoviscidos*.ti,ab,kw. | 0 |
| 33 | (development* adj2 (delay* or deviat* or disabilit* or disabled or disorder?)).ti,ab,kw. | 15 |
| 34 | ((autoimmune or brittle or insulin-dependent or juvenile) adj3 diabet*).ti,ab,kw. | 7 |
| 35 | (("Type 1" or "Type I" or ID) adj DM).ti,ab,kw. | 0 |
| 36 | (IDDM or T1D).ti,ab,kw. | 8 |
| 37 | (Down* adj2 syndrome*).ti,ab,kw. | 3 |
| 38 | (mongolism* or mongoloid*).ti,ab,kw. | 0 |
| 39 | ("trisomy 21" or "trisomy G1" or "trisomy (G)1" or "trisomy G-1" or "trisomy GM" or "trisomy G" or "21 trisomy" or "G1 trisomy" or "G(1) trisomy" or "G-1 trisomy" or "GM trisomy" or "G trisomy").ti,ab,kw. | 1 |
| 40 | ((chromosom* adj1 triplicat*) or trisom*).ti,ab,kw. | 1 |
| 41 | (translocat* adj2 DS).ti,ab,kw. | 0 |
| 42 | (petit mal or grand mal or absence status).ti,ab,kw. | 0 |
| 43 | (epileps* or epilept* or seizure* or convulsi*).ti,ab,kw. | 34 |
| 44 | ((Dravet* or Landau-Kleffner* or Lennox Gastaut* or Doose* or Ohtahara* or Sturge-Weber* or West or "West's") adj2 syndrome*).ti,ab,kw. | 0 |
| 45 | ((sturge* or weber*) adj2 (disease* or syndrome*)).ti,ab,kw. | 0 |
| 46 | (myoclonic encephalopath* or action myoclonus-renal failure syndrome* or atypical inclusion-body disease* or biotin-responsive encephalopath* or haw river syndrome* or may white syndrome* or myoclonus-nephropathy syndrome* or naito oyanagi disease*).ti,ab,kw. | 0 |
| 47 | SMEI.ti,ab,kw. | 0 |
| 48 | (MERRF or fukuhara disease* or fukuhara disorder* or myoencephalopathy ragged-red fiber disease* or myoencephalopathy ragged-red fibre disease*).ti,ab,kw. | 0 |
| 49 | Lafora.ti,ab,kw. | 0 |
| 50 | ((Unverricht* adj1 Lundborg) or Baltic Myoclonus or Unverricht disease* or Unverricht* syndrome*).ti,ab,kw. | 0 |
| 51 | ((infantile or nodding) adj2 spasm?).ti,ab,kw. | 0 |
| 52 | hypsarrhythmi*.ti,ab,kw. | 0 |
| 53 | ((cardiac* or cardio* or heart*) adj2 (abnormalit* or anomal* or atypical* or a-typical* or defect* or deficien* or deform* or disorder? or impair* or malform*)).ti,ab,kw. | 13 |
| 54 | (tetralog* adj2 fallot*).ti,ab,kw. | 1 |
| 55 | ((cardiac* or cardio* or heart?) adj5 (congenital* or inborn* or hereditar* or inherit*)).ti,ab,kw. | 10 |
| 56 | ((genetic or hereditary or inherited or inborn) adj2 (condition* or disease* or disorder*)).ti,ab,kw. | 7 |
| 57 | single gene defect?.ti,ab,kw. | 0 |
| 58 | (haemophili* or hemophili* or (("factor VIII" or "factor 8") adj3 deficien*)).ti,ab,kw. | 2 |
| 59 | ((blood or haematologic* or hematologic*) adj2 (condition* or disease* or disorder*)).ti,ab,kw. | 3 |
| 60 | (HIV-1 or HIV1 or HIV-I or HIVI or (HIV adj1 type 1) or (HIV adj1 type I)).ti,ab,kw. | 1 |
| 61 | (HIV-2 or HIV2 or HIV-II or HIVII or (HIV adj1 type 2) or (HIV adj1 type II)).ti,ab,kw. | 2 |
| 62 | (immunodeficiency virus* adj2 human*).ti,ab,kw. | 23 |
| 63 | (immuno-deficiency virus* adj2 human*).ti,ab,kw. | 2 |
| 64 | ((acquired immunodeficiency or acquired immuno-deficiency) adj2 virus*).ti,ab,kw. | 2 |
| 65 | AIDS virus*.ti,ab,kw. | 1 |
| 66 | ((kidney* or renal) adj2 (condition* or disease* or disorder*)).ti,ab,kw. | 52 |
| 67 | (meningomyelocele or meningo-myelocele or myelocele).ti,ab,kw. | 1 |
| 68 | ((musculoskelet* or musculo-skelet* or muscle* or muscular or skelet* or orthop?edic*) adj2 (abnormalit* or deformit* or disorder* or disease*)).ti,ab,kw. | 18 |
| 69 | (MSD mor MSDs or MSKD or MSKDs).ti,ab,kw. | 0 |
| 70 | ((spina* or status) adj (bifida* or dysraphi*)).ti,ab,kw. | 2 |
| 71 | (((cleft or open) adj spine*) or rachischis* or schistorrhach*).ti,ab,kw. | 0 |
| 72 | (bowel* adj3 inflammatory adj3 (condition* or disease* or illness*)).ti,ab,kw. | 10 |
| 73 | (celiac or coeliac).ti,ab,kw. | 1 |
| 74 | pain.ti. | 325 |
| 75 | (chromosomal adj2 (anomal* or abberation* or abnormalit*)).ti,ab,kw. | 1 |
| 76 | ((cranio facial or craniofacial) adj2 (abnormalit* or anomal*)).ti,ab,kw. | 1 |
| 77 | ((cleft or lip) adj2 palate).ti,ab,kw. | 6 |
| 78 | Bronchopulmonary Dysplasia.ti,ab,kw. | 2 |
| 79 | scoliosis.ti,ab,kw. | 7 |
| 80 | (skin adj2 (disease* or disorder*)).ti,ab,kw. | 4 |
| 81 | eczema.ti,ab,kw. | 13 |
| 82 | (sleep adj2 (apnea or disorder*)).ti,ab,kw. | 30 |
| 83 | hydrocephal*.ti,ab,kw. | 1 |
| 84 | or/1-83 | 1245 |
| 85 | (social adj3 (support* or network* or system*)).ti,ab,kw. | 72 |
| 86 | (support adj3 (group* or system* or emotion* or physical)).ti,ab,kw. | 72 |
| 87 | ((psychosocial or psycho-social or biopsychosocial) adj3 (support* or intervention* or program* or care or outcome* or therap* or distress or problem* or need* or adjust* or wellbeing or well-being or wellness or burden* or factor*)).ti,ab,kw. | 101 |
| 88 | ((psycho* or behavior* or behaviour* or education* or group* or cogniti* or family) adj3 therapy).ti,ab,kw. | 99 |
| 89 | ((cope* or coping) adj3 (skill* or strateg* or intervention* or program* or behavio*)).ti,ab,kw. | 45 |
| 90 | ((psychologic* or emotion* or stress or distress or anxiety or depress*) adj3 (adjust* or adapt* or reduce or reduction or support* or well-being or wellbeing or wellness or burden* or factor*)).ti,ab,kw. | 200 |
| 91 | self-efficacy.ti,ab,kw. | 64 |
| 92 | (mental health adj3 (need* or care or support* or intervention* or program*)).ti,ab,kw. | 46 |
| 93 | (spiritual* or pastoral or religio*).ti,ab,kw. | 77 |
| 94 | ((financial or economic or occupation* or work or employ* or job*) adj3 (stress* or toxicity or burden* or insecurit*)).ti,ab,kw. | 50 |
| 95 | (information adj3 (need* or requirement* or health or request* or seek*)).ti,ab,kw. | 59 |
| 96 | or/85-95 | 613 |
| 97 | ((pediatric* or paediatric*) adj3 (room* or department* or facilit*)).ti,ab,kw. | 6 |
| 98 | (ward* or wards or unit or units).ti,ab,kw. | 287 |
| 99 | (PICU* or ICU or ICUS or NICU* or intensive care or critical care or hospital* or inpatient*).ti,ab,kw. | 875 |
| 100 | (discharg* or postdischarg* or post-discharg* or readmission or re-admission or readmit* or re-admit*).ti,ab,kw. | 206 |
| 101 | (transition* adj3 home).ti,ab,kw. | 5 |
| 102 | (hospital adj3 home).ti,ab,kw. | 27 |
| 103 | ((transfer* or coordinat* or transitional) adj3 care).ti,ab,kw. | 51 |
| 104 | (home adj3 (care or healthcare)).ti,ab,kw. | 73 |
| 105 | or/97-104 | 1059 |
| 106 | (pediatric* or paediatric* or child*).ti,ab,kw. | 792 |
| 107 | (boy or boys or girl* or juvenile* or teen* or tween* or preteen* or pre-teen* or youth* or adolesc* or prepubesc* or pubescen*or pre-pubesc*).ti,ab,kw. | 253 |
| 108 | (preschool* or pre-school* or school-age* or toddler* or infant* or baby or babies or newborn* or neonate*).ti,ab,kw. | 311 |
| 109 | or/106-108 | 1074 |
| 110 | (intervention* or program* or training or course* or education or project* or curriculum or model* or framework*).ti,ab,kw. | 2107 |
| 111 | 84 and 96 and 105 and 109 and 110 | 29 |
| 112 | limit 111 to yr="2000 -Current" | 29 |

**Database:**
EBM Reviews - Cochrane Central Register of Controlled Trials <April 2024>

| **#** | **Query** | **Results from 7 May 2024** |
| --- | --- | --- |
| 1 | Chronic Disease/ or Disabled Children/ | 16,591 |
| 2 | exp Anemia/ or Anemia, Sickle Cell/ or arthritis/ or arthritis, juvenile/ or asthma/ or asthma, exercise-induced/ or autism spectrum disorder/ or asperger syndrome/ or autistic disorder/ or brain injuries/ or brain injuries, traumatic/ or brain injury, chronic/ or Brain Damage, Chronic/ or Cerebral Palsy/ or Congenital Abnormalities/ or Cystic Fibrosis/ or Developmental Disabilities/ or diabetes mellitus/ or diabetes mellitus, type 1/ | 54,905 |
| 3 | Down Syndrome/ or "Chromosomes, Human, Pair 21"/ or Trisomy/ or exp Epilepsy/ or Heart Diseases/ or exp Heart Defects, Congenital/ or Genetic Diseases, Inborn/ or Hemophilia A/ or Hematologic Diseases/ or hiv/ or hiv-1/ or hiv-2/ or exp HIV Infections/ or Intellectual Disability/ | 29,997 |
| 4 | exp Kidney Diseases/ or Meningomyelocele/ or exp Musculoskeletal Diseases/ or exp Spinal Dysraphism/ or Child Development Disorders, Pervasive/ or Celiac Disease/ or inflammatory bowel diseases/ or colitis, ulcerative/ or crohn disease/ or Complex Regional Pain Syndromes/ or exp Chromosome Aberrations/ or Craniofacial Abnormalities/ or Cleft Palate/ or Bronchopulmonary Dysplasia/ or Scoliosis/ or exp Skin Diseases/ or Eczema/ or sleep apnea syndromes/ or sleep apnea, central/ or sleep apnea, obstructive/ or Hydrocephalus/ | 145,383 |
| 5 | ((chronic* or critical or longterm or long-term) adj4 (disease* or disorder* or illness* or condition* or pain or syndrome*)).ti,ab,kw. | 98,637 |
| 6 | (life-limiting adj2 (disease* or disorder* or illness* or condition* or disabilit*)).ti,ab,kw. | 200 |
| 7 | ((special health care or special healthcare) adj3 (need* or condition* or disease* or disorder* or illness*)).ti,ab,kw. | 73 |
| 8 | Medically at-risk.ti,ab,kw. | 9 |
| 9 | (complex adj4 (chronic condition* or health condition* or medical condition* or care need* or medical need* or healthcare need* or health care need* or health need* or disabilit*)).ti,ab,kw. | 362 |
| 10 | (medical* adj2 (complex* or fragile*)).ti,ab,kw. | 581 |
| 11 | (health adj2 (complex* or fragile*)).ti,ab,kw. | 388 |
| 12 | (Technolog* adj2 dependen*).ti,ab,kw. | 54 |
| 13 | (ventilator adj2 (dependent* or assist*)).ti,ab,kw. | 351 |
| 14 | (mechanical* adj2 assist* adj2 ventilat*).ti,ab,kw. | 85 |
| 15 | mechanically ventilated.ti,ab,kw. | 3,381 |
| 16 | medically fragile technology dependent*.ti,ab,kw. | 0 |
| 17 | polyhandicap*.ti,ab,kw. | 4 |
| 18 | (profound adj4 (intellectual or disabilit*)).ti,ab,kw. | 74 |
| 19 | ((intellectual* or brain* or cogniti* or mental) adj2 (deficit* or delay* or deviation* or disabil* or disabled or disorder* or dysfunction* or handicap* or impair* or retard*)).ti,ab,kw. | 35,459 |
| 20 | (deformit* or disabled or disabilit* or handicap*).ti,ab,kw. | 54,169 |
| 21 | ((mentally or intellectually or physically) adj1 challenged).ti,ab,kw. | 23 |
| 22 | (Anemia or anaemia or Thalassemia).ti,ab,kw. | 23,165 |
| 23 | ((HbS or hemoglobin or haemoglobin or sickle cell or sickling) adj2 (condition* or disease* or disorder*)).ti,ab,kw. | 1,808 |
| 24 | (sickle cell adj2 anemia*).ti,ab,kw. | 1,168 |
| 25 | (arthriti* or periarthriti* or peri arthriti* or polyarthriti* or poly arthriti*).ti,ab,kw. | 27,248 |
| 26 | asthma*.ti,ab,kw. | 37,038 |
| 27 | (autis* or asperger* or kanner* syndrome*).ti,ab,kw. | 5,406 |
| 28 | (brain* adj3 (injur* or commotio* or damag* or trauma*)).ti,ab,kw. | 9,601 |
| 29 | (tbi or tbis or mtbi or concussion*).ti,ab,kw. | 4,242 |
| 30 | (cerebral palsy or (diplegia adj1 spastic) or Little* disease).ti,ab,kw. | 4,736 |
| 31 | ((brain* or central*) adj2 palsy).ti,ab,kw. | 43 |
| 32 | ((brain or central* or cerebral*) adj2 (paralys* or paresis or pareses)).ti,ab,kw. | 75 |
| 33 | ((congenital* or birth) adj2 (abnormal* or anomal* or defect* or deform* or malform*)).ti,ab,kw. | 4,108 |
| 34 | cystic fibros*.ti,ab,kw. | 6,092 |
| 35 | ((fibrocystic or fibro-cystic) adj3 pancrea*).ti,ab,kw. | 0 |
| 36 | mucoviscidos*.ti,ab,kw. | 48 |
| 37 | (development* adj2 (delay* or deviat* or disabilit* or disabled or disorder?)).ti,ab,kw. | 3,036 |
| 38 | ((autoimmune or brittle or insulin-dependent or juvenile) adj3 diabet*).ti,ab,kw. | 26,399 |
| 39 | (("Type 1" or "Type I" or ID) adj DM).ti,ab,kw. | 168 |
| 40 | (IDDM or T1D).ti,ab,kw. | 2,990 |
| 41 | (Down* adj2 syndrome*).ti,ab,kw. | 981 |
| 42 | (mongolism* or mongoloid*).ti,ab,kw. | 8 |
| 43 | ("trisomy 21" or "trisomy G1" or "trisomy (G)1" or "trisomy G-1" or "trisomy GM" or "trisomy G" or "21 trisomy" or "G1 trisomy" or "G(1) trisomy" or "G-1 trisomy" or "GM trisomy" or "G trisomy").ti,ab,kw. | 121 |
| 44 | ((chromosom* adj1 triplicat*) or trisom*).ti,ab,kw. | 294 |
| 45 | (translocat* adj2 DS).ti,ab,kw. | 0 |
| 46 | (petit mal or grand mal or absence status).ti,ab,kw. | 86 |
| 47 | (epileps* or epilept* or seizure* or convulsi*).ti,ab,kw. | 14,916 |
| 48 | ((Dravet* or Landau-Kleffner* or Lennox Gastaut* or Doose* or Ohtahara* or Sturge-Weber* or West or "West's") adj2 syndrome*).ti,ab,kw. | 528 |
| 49 | ((sturge* or weber*) adj2 (disease* or syndrome*)).ti,ab,kw. | 68 |
| 50 | (myoclonic encephalopath* or action myoclonus-renal failure syndrome* or atypical inclusion-body disease* or biotin-responsive encephalopath* or haw river syndrome* or may white syndrome* or myoclonus-nephropathy syndrome* or naito oyanagi disease*).ti,ab,kw. | 2 |
| 51 | SMEI.ti,ab,kw. | 9 |
| 52 | (MERRF or fukuhara disease* or fukuhara disorder* or myoencephalopathy ragged-red fiber disease* or myoencephalopathy ragged-red fibre disease*).ti,ab,kw. | 3 |
| 53 | Lafora.ti,ab,kw. | 4 |
| 54 | ((Unverricht* adj1 Lundborg) or Baltic Myoclonus or Unverricht disease* or Unverricht* syndrome*).ti,ab,kw. | 11 |
| 55 | ((infantile or nodding) adj2 spasm?).ti,ab,kw. | 277 |
| 56 | hypsarrhythmi*.ti,ab,kw. | 75 |
| 57 | ((cardiac* or cardio* or heart*) adj2 (abnormalit* or anomal* or atypical* or a-typical* or defect* or deficien* or deform* or disorder? or impair* or malform*)).ti,ab,kw. | 4,565 |
| 58 | (tetralog* adj2 fallot*).ti,ab,kw. | 305 |
| 59 | ((cardiac* or cardio* or heart?) adj5 (congenital* or inborn* or hereditar* or inherit*)).ti,ab,kw. | 2,911 |
| 60 | ((genetic or hereditary or inherited or inborn) adj2 (condition* or disease* or disorder*)).ti,ab,kw. | 2,880 |
| 61 | single gene defect?.ti,ab,kw. | 2 |
| 62 | (haemophili* or hemophili* or (("factor VIII" or "factor 8") adj3 deficien*)).ti,ab,kw. | 1,839 |
| 63 | ((blood or haematologic* or hematologic*) adj2 (condition* or disease* or disorder*)).ti,ab,kw. | 8,069 |
| 64 | (HIV-1 or HIV1 or HIV-I or HIVI or (HIV adj1 type 1) or (HIV adj1 type I)).ti,ab,kw. | 6,519 |
| 65 | (HIV-2 or HIV2 or HIV-II or HIVII or (HIV adj1 type 2) or (HIV adj1 type II)).ti,ab,kw. | 102 |
| 66 | (immunodeficiency virus* adj2 human*).ti,ab,kw. | 13,397 |
| 67 | (immuno-deficiency virus* adj2 human*).ti,ab,kw. | 20 |
| 68 | ((acquired immunodeficiency or acquired immuno-deficiency) adj2 virus*).ti,ab,kw. | 50 |
| 69 | AIDS virus*.ti,ab,kw. | 10 |
| 70 | ((kidney* or renal) adj2 (condition* or disease* or disorder*)).ti,ab,kw. | 25,265 |
| 71 | (meningomyelocele or meningo-myelocele or myelocele).ti,ab,kw. | 90 |
| 72 | ((musculoskelet* or musculo-skelet* or muscle* or muscular or skelet* or orthop?edic*) adj2 (abnormalit* or deformit* or disorder* or disease*)).ti,ab,kw. | 8,606 |
| 73 | (MSD mor MSDs or MSKD or MSKDs).ti,ab,kw. | 12 |
| 74 | ((spina* or status) adj (bifida* or dysraphi*)).ti,ab,kw. | 240 |
| 75 | (((cleft or open) adj spine*) or rachischis* or schistorrhach*).ti,ab,kw. | 13 |
| 76 | (bowel* adj3 inflammatory adj3 (condition* or disease* or illness*)).ti,ab,kw. | 4,090 |
| 77 | (celiac or coeliac).ti,ab,kw. | 1,494 |
| 78 | pain.ti. | 75,941 |
| 79 | (chromosomal adj2 (anomal* or abberation* or abnormalit*)).ti,ab,kw. | 378 |
| 80 | ((cranio facial or craniofacial) adj2 (abnormalit* or anomal*)).ti,ab,kw. | 88 |
| 81 | ((cleft or lip) adj2 palate).ti,ab,kw. | 974 |
| 82 | Bronchopulmonary Dysplasia.ti,ab,kw. | 1,614 |
| 83 | scoliosis.ti,ab,kw. | 1,675 |
| 84 | (skin adj2 (disease* or disorder*)).ti,ab,kw. | 5,329 |
| 85 | eczema.ti,ab,kw. | 4,169 |
| 86 | (sleep adj2 (apnea or disorder*)).ti,ab,kw. | 14,195 |
| 87 | hydrocephal*.ti,ab,kw. | 795 |
| 88 | or/1-87 | 569,612 |
| 89 | social support/ or community support/ or family support/ or psychosocial support systems/ | 4,322 |
| 90 | psychotherapy/ or animal assisted therapy/ or aromatherapy/ or art therapy/ or behavior therapy/ or cognitive behavioral therapy/ or "acceptance and commitment therapy"/ or mindfulness/ or dialectical behavior therapy/ or relaxation therapy/ or meditation/ or imagery, psychotherapy/ or interpersonal psychotherapy/ or mentalization-based therapy/ or music therapy/ or narrative therapy/ or person-centered psychotherapy/ or play therapy/ or psychotherapy, brief/ or psychotherapy, multiple/ or psychotherapy, psychodynamic/ or psychotherapy, rational-emotive/ or socioenvironmental therapy/ | 27,424 |
| 91 | mind-body therapies/ or biofeedback, psychology/ or breathing exercises/ or hypnosis/ or laughter therapy/ or mental healing/ or psychodrama/ or relaxation therapy/ or tai ji/ or therapeutic touch/ or yoga/ | 6,616 |
| 92 | sensory art therapies/ or color therapy/ or dance therapy/ or Adaptation, Psychological/ or Coping Skills/ or Psychosocial Intervention/ or self efficacy/ or Emotional Adjustment/ or counseling/ or directive counseling/ or distance counseling/ or "Quality of Life"/px or Stress, Psychological/pc, px or Anxiety/pc, px or Depression/pc, px or Spiritual Therapies/ or spirituality/ or pastoral care/ or financial stress/ or occupational stress/ or Information Seeking Behavior/ | 16,128 |
| 93 | (social adj3 (support* or network* or system*)).ti,ab,kw. | 12,198 |
| 94 | (support adj3 (group* or system* or emotion* or physical)).ti,ab,kw. | 10,008 |
| 95 | ((psychosocial or psycho-social or biopsychosocial) adj3 (support* or intervention* or program* or care or outcome* or therap* or distress or problem* or need* or adjust* or wellbeing or well-being or wellness or burden* or factor*)).ti,ab,kw. | 12,947 |
| 96 | ((psycho* or behavior* or behaviour* or education* or group* or cogniti* or family) adj3 therapy).ti,ab,kw. | 61,331 |
| 97 | ((cope* or coping) adj3 (skill* or strateg* or intervention* or program* or behavio*)).ti,ab,kw. | 6,862 |
| 98 | ((psychologic* or emotion* or stress or distress or anxiety or depress*) adj3 (adjust* or adapt* or reduce or reduction or support* or well-being or wellbeing or wellness or burden* or factor*)).ti,ab,kw. | 35,516 |
| 99 | self-efficacy.ti,ab,kw. | 18,633 |
| 100 | (mental health adj3 (need* or care or support* or intervention* or program*)).ti,ab,kw. | 6,165 |
| 101 | (spiritual* or pastoral or religio*).ti,ab,kw. | 4,457 |
| 102 | ((financial or economic or occupation* or work or employ* or job*) adj3 (stress* or toxicity or burden* or insecurit*)).ti,ab,kw. | 4,613 |
| 103 | (information adj3 (need* or requirement* or health or request* or seek*)).ti,ab,kw. | 6,693 |
| 104 | or/89-103 | 167,637 |
| 105 | patients/ or adolescent, hospitalized/ or child, hospitalized/ or inpatients/ | 2,529 |
| 106 | Hospitals/ or Hospitals, Pediatric/ or Hospital Units/ or hospitalization/ or patient admission/ | 10,290 |
| 107 | Intensive Care Units, Pediatric/ or Intensive Care Units, Neonatal/ | 1,686 |
| 108 | "continuity of patient care"/ or patient discharge/ or Transitional Care/ or Patient Readmission/ | 4,504 |
| 109 | ((pediatric* or paediatric*) adj3 (room* or department* or facilit*)).ti,ab,kw. | 2,226 |
| 110 | (ward* or wards or unit or units).ti,ab,kw. | 117,530 |
| 111 | (PICU* or ICU or ICUS or NICU* or intensive care or critical care or hospital* or inpatient*).ti,ab,kw. | 267,886 |
| 112 | (discharg* or postdischarg* or post-discharg* or readmission or re-admission or readmit* or re-admit*).ti,ab,kw. | 55,790 |
| 113 | (transition* adj3 home).ti,ab,kw. | 244 |
| 114 | (hospital adj3 home).ti,ab,kw. | 2,357 |
| 115 | ((transfer* or coordinat* or transitional) adj3 care).ti,ab,kw. | 2,837 |
| 116 | (home adj3 (care or healthcare)).ti,ab,kw. | 6,269 |
| 117 | or/105-116 | 355,099 |
| 118 | adolescent/ or child/ or child, preschool/ | 178,376 |
| 119 | infant/ or infant, newborn/ | 44,916 |
| 120 | (pediatric* or paediatric* or child*).ti,ab,kw. | 190,457 |
| 121 | (boy or boys or girl* or juvenile* or teen* or tween* or preteen* or pre-teen* or youth* or adolesc* or prepubesc* or pubescen*or pre-pubesc*).ti,ab,kw. | 77,578 |
| 122 | (preschool* or pre-school* or school-age* or toddler* or infant* or baby or babies or newborn* or neonate*).ti,ab,kw. | 83,368 |
| 123 | or/118-122 | 367,830 |
| 124 | Psychosocial Intervention/ | 258 |
| 125 | Health Education/ or pilot projects/ or program evaluation/ or Program Development/ | 41,625 |
| 126 | (intervention* or program* or training or course* or education or project* or curriculum or model* or framework*).ti,ab,kw. | 873,184 |
| 127 | or/124-126 | 888,126 |
| 128 | 88 and 104 and 117 and 123 and 127 | 2,504 |
| 129 | limit 128 to yr="2000 -Current" | 2,419 |

**Database:**
EBM Reviews - Cochrane Database of Systematic Reviews <2005 to May 1, 2024>

| **#** | **Query** | **Results from 7 May 2024** |
| --- | --- | --- |
| 1 | ((chronic* or critical or longterm or long-term) adj4 (disease* or disorder* or illness* or condition* or pain or syndrome*)).ti,ab,kw. | 1,396 |
| 2 | (life-limiting adj2 (disease* or disorder* or illness* or condition* or disabilit*)).ti,ab,kw. | 40 |
| 3 | ((special health care or special healthcare) adj3 (need* or condition* or disease* or disorder* or illness*)).ti,ab,kw. | 0 |
| 4 | Medically at-risk.ti,ab,kw. | 0 |
| 5 | (complex adj4 (chronic condition* or health condition* or medical condition* or care need* or medical need* or healthcare need* or health care need* or health need* or disabilit*)).ti,ab,kw. | 6 |
| 6 | (medical* adj2 (complex* or fragile*)).ti,ab,kw. | 2 |
| 7 | (health adj2 (complex* or fragile*)).ti,ab,kw. | 3 |
| 8 | (Technolog* adj2 dependen*).ti,ab,kw. | 1 |
| 9 | (ventilator adj2 (dependent* or assist*)).ti,ab,kw. | 3 |
| 10 | (mechanical* adj2 assist* adj2 ventilat*).ti,ab,kw. | 2 |
| 11 | mechanically ventilated.ti,ab,kw. | 49 |
| 12 | medically fragile technology dependent*.ti,ab,kw. | 0 |
| 13 | polyhandicap*.ti,ab,kw. | 0 |
| 14 | (profound adj4 (intellectual or disabilit*)).ti,ab,kw. | 0 |
| 15 | ((intellectual* or brain* or cogniti* or mental) adj2 (deficit* or delay* or deviation* or disabil* or disabled or disorder* or dysfunction* or handicap* or impair* or retard*)).ti,ab,kw. | 515 |
| 16 | (deformit* or disabled or disabilit* or handicap*).ti,ab,kw. | 719 |
| 17 | ((mentally or intellectually or physically) adj1 challenged).ti,ab,kw. | 0 |
| 18 | (Anemia or anaemia or Thalassemia).ti,ab,kw. | 234 |
| 19 | ((HbS or hemoglobin or haemoglobin or sickle cell or sickling) adj2 (condition* or disease* or disorder*)).ti,ab,kw. | 68 |
| 20 | (sickle cell adj2 anemia*).ti,ab,kw. | 51 |
| 21 | (arthriti* or periarthriti* or peri arthriti* or polyarthriti* or poly arthriti*).ti,ab,kw. | 189 |
| 22 | asthma*.ti,ab,kw. | 320 |
| 23 | (autis* or asperger* or kanner* syndrome*).ti,ab,kw. | 42 |
| 24 | (brain* adj3 (injur* or commotio* or damag* or trauma*)).ti,ab,kw. | 101 |
| 25 | (tbi or tbis or mtbi or concussion*).ti,ab,kw. | 30 |
| 26 | (cerebral palsy or (diplegia adj1 spastic) or Little* disease).ti,ab,kw. | 73 |
| 27 | ((brain* or central*) adj2 palsy).ti,ab,kw. | 1 |
| 28 | ((brain or central* or cerebral*) adj2 (paralys* or paresis or pareses)).ti,ab,kw. | 0 |
| 29 | ((congenital* or birth) adj2 (abnormal* or anomal* or defect* or deform* or malform*)).ti,ab,kw. | 71 |
| 30 | cystic fibros*.ti,ab,kw. | 234 |
| 31 | ((fibrocystic or fibro-cystic) adj3 pancrea*).ti,ab,kw. | 0 |
| 32 | mucoviscidos*.ti,ab,kw. | 0 |
| 33 | (development* adj2 (delay* or deviat* or disabilit* or disabled or disorder?)).ti,ab,kw. | 83 |
| 34 | ((autoimmune or brittle or insulin-dependent or juvenile) adj3 diabet*).ti,ab,kw. | 12 |
| 35 | (("Type 1" or "Type I" or ID) adj DM).ti,ab,kw. | 0 |
| 36 | (IDDM or T1D).ti,ab,kw. | 2 |
| 37 | (Down* adj2 syndrome*).ti,ab,kw. | 25 |
| 38 | (mongolism* or mongoloid*).ti,ab,kw. | 0 |
| 39 | ("trisomy 21" or "trisomy G1" or "trisomy (G)1" or "trisomy G-1" or "trisomy GM" or "trisomy G" or "21 trisomy" or "G1 trisomy" or "G(1) trisomy" or "G-1 trisomy" or "GM trisomy" or "G trisomy").ti,ab,kw. | 2 |
| 40 | ((chromosom* adj1 triplicat*) or trisom*).ti,ab,kw. | 2 |
| 41 | (translocat* adj2 DS).ti,ab,kw. | 0 |
| 42 | (petit mal or grand mal or absence status).ti,ab,kw. | 0 |
| 43 | (epileps* or epilept* or seizure* or convulsi*).ti,ab,kw. | 257 |
| 44 | ((Dravet* or Landau-Kleffner* or Lennox Gastaut* or Doose* or Ohtahara* or Sturge-Weber* or West or "West's") adj2 syndrome*).ti,ab,kw. | 8 |
| 45 | ((sturge* or weber*) adj2 (disease* or syndrome*)).ti,ab,kw. | 0 |
| 46 | (myoclonic encephalopath* or action myoclonus-renal failure syndrome* or atypical inclusion-body disease* or biotin-responsive encephalopath* or haw river syndrome* or may white syndrome* or myoclonus-nephropathy syndrome* or naito oyanagi disease*).ti,ab,kw. | 0 |
| 47 | SMEI.ti,ab,kw. | 1 |
| 48 | (MERRF or fukuhara disease* or fukuhara disorder* or myoencephalopathy ragged-red fiber disease* or myoencephalopathy ragged-red fibre disease*).ti,ab,kw. | 0 |
| 49 | Lafora.ti,ab,kw. | 0 |
| 50 | ((Unverricht* adj1 Lundborg) or Baltic Myoclonus or Unverricht disease* or Unverricht* syndrome*).ti,ab,kw. | 0 |
| 51 | ((infantile or nodding) adj2 spasm?).ti,ab,kw. | 2 |
| 52 | hypsarrhythmi*.ti,ab,kw. | 1 |
| 53 | ((cardiac* or cardio* or heart*) adj2 (abnormalit* or anomal* or atypical* or a-typical* or defect* or deficien* or deform* or disorder? or impair* or malform*)).ti,ab,kw. | 55 |
| 54 | (tetralog* adj2 fallot*).ti,ab,kw. | 0 |
| 55 | ((cardiac* or cardio* or heart?) adj5 (congenital* or inborn* or hereditar* or inherit*)).ti,ab,kw. | 32 |
| 56 | ((genetic or hereditary or inherited or inborn) adj2 (condition* or disease* or disorder*)).ti,ab,kw. | 263 |
| 57 | single gene defect?.ti,ab,kw. | 1 |
| 58 | (haemophili* or hemophili* or (("factor VIII" or "factor 8") adj3 deficien*)).ti,ab,kw. | 27 |
| 59 | ((blood or haematologic* or hematologic*) adj2 (condition* or disease* or disorder*)).ti,ab,kw. | 294 |
| 60 | (HIV-1 or HIV1 or HIV-I or HIVI or (HIV adj1 type 1) or (HIV adj1 type I)).ti,ab,kw. | 33 |
| 61 | (HIV-2 or HIV2 or HIV-II or HIVII or (HIV adj1 type 2) or (HIV adj1 type II)).ti,ab,kw. | 5 |
| 62 | (immunodeficiency virus* adj2 human*).ti,ab,kw. | 36 |
| 63 | (immuno-deficiency virus* adj2 human*).ti,ab,kw. | 1 |
| 64 | ((acquired immunodeficiency or acquired immuno-deficiency) adj2 virus*).ti,ab,kw. | 3 |
| 65 | AIDS virus*.ti,ab,kw. | 0 |
| 66 | ((kidney* or renal) adj2 (condition* or disease* or disorder*)).ti,ab,kw. | 365 |
| 67 | (meningomyelocele or meningo-myelocele or myelocele).ti,ab,kw. | 1 |
| 68 | ((musculoskelet* or musculo-skelet* or muscle* or muscular or skelet* or orthop?edic*) adj2 (abnormalit* or deformit* or disorder* or disease*)).ti,ab,kw. | 75 |
| 69 | (MSD mor MSDs or MSKD or MSKDs).ti,ab,kw. | 0 |
| 70 | ((spina* or status) adj (bifida* or dysraphi*)).ti,ab,kw. | 3 |
| 71 | (((cleft or open) adj spine*) or rachischis* or schistorrhach*).ti,ab,kw. | 0 |
| 72 | (bowel* adj3 inflammatory adj3 (condition* or disease* or illness*)).ti,ab,kw. | 117 |
| 73 | (celiac or coeliac).ti,ab,kw. | 2 |
| 74 | pain.ti. | 559 |
| 75 | (chromosomal adj2 (anomal* or abberation* or abnormalit*)).ti,ab,kw. | 4 |
| 76 | ((cranio facial or craniofacial) adj2 (abnormalit* or anomal*)).ti,ab,kw. | 45 |
| 77 | ((cleft or lip) adj2 palate).ti,ab,kw. | 11 |
| 78 | Bronchopulmonary Dysplasia.ti,ab,kw. | 83 |
| 79 | scoliosis.ti,ab,kw. | 10 |
| 80 | (skin adj2 (disease* or disorder*)).ti,ab,kw. | 256 |
| 81 | eczema.ti,ab,kw. | 57 |
| 82 | (sleep adj2 (apnea or disorder*)).ti,ab,kw. | 73 |
| 83 | hydrocephal*.ti,ab,kw. | 25 |
| 84 | or/1-83 | 4,623 |
| 85 | (social adj3 (support* or network* or system*)).ti,ab,kw. | 87 |
| 86 | (support adj3 (group* or system* or emotion* or physical)).ti,ab,kw. | 66 |
| 87 | ((psychosocial or psycho-social or biopsychosocial) adj3 (support* or intervention* or program* or care or outcome* or therap* or distress or problem* or need* or adjust* or wellbeing or well-being or wellness or burden* or factor*)).ti,ab,kw. | 445 |
| 88 | ((psycho* or behavior* or behaviour* or education* or group* or cogniti* or family) adj3 therapy).ti,ab,kw. | 616 |
| 89 | ((cope* or coping) adj3 (skill* or strateg* or intervention* or program* or behavio*)).ti,ab,kw. | 24 |
| 90 | ((psychologic* or emotion* or stress or distress or anxiety or depress*) adj3 (adjust* or adapt* or reduce or reduction or support* or well-being or wellbeing or wellness or burden* or factor*)).ti,ab,kw. | 264 |
| 91 | self-efficacy.ti,ab,kw. | 61 |
| 92 | (mental health adj3 (need* or care or support* or intervention* or program*)).ti,ab,kw. | 34 |
| 93 | (spiritual* or pastoral or religio*).ti,ab,kw. | 27 |
| 94 | ((financial or economic or occupation* or work or employ* or job*) adj3 (stress* or toxicity or burden* or insecurit*)).ti,ab,kw. | 43 |
| 95 | (information adj3 (need* or requirement* or health or request* or seek*)).ti,ab,kw. | 215 |
| 96 | or/85-95 | 1,376 |
| 97 | ((pediatric* or paediatric*) adj3 (room* or department* or facilit*)).ti,ab,kw. | 5 |
| 98 | (ward* or wards or unit or units).ti,ab,kw. | 702 |
| 99 | (PICU* or ICU or ICUS or NICU* or intensive care or critical care or hospital* or inpatient*).ti,ab,kw. | 2,094 |
| 100 | (discharg* or postdischarg* or post-discharg* or readmission or re-admission or readmit* or re-admit*).ti,ab,kw. | 423 |
| 101 | (transition* adj3 home).ti,ab,kw. | 0 |
| 102 | (hospital adj3 home).ti,ab,kw. | 46 |
| 103 | ((transfer* or coordinat* or transitional) adj3 care).ti,ab,kw. | 34 |
| 104 | (home adj3 (care or healthcare)).ti,ab,kw. | 95 |
| 105 | or/97-104 | 2,482 |
| 106 | (pediatric* or paediatric* or child*).ti,ab,kw. | 3,927 |
| 107 | (boy or boys or girl* or juvenile* or teen* or tween* or preteen* or pre-teen* or youth* or adolesc* or prepubesc* or pubescen*or pre-pubesc*).ti,ab,kw. | 948 |
| 108 | (preschool* or pre-school* or school-age* or toddler* or infant* or baby or babies or newborn* or neonate*).ti,ab,kw. | 1,681 |
| 109 | or/106-108 | 4,229 |
| 110 | (intervention* or program* or training or course* or education or project* or curriculum or model* or framework*).ti,ab,kw. | 7,707 |
| 111 | 84 and 96 and 105 and 109 and 110 | 55 |
| 112 | limit 111 to yr="2000 -Current" | 38 |

CINAHL PLUS WITH FULL TEXT (Ebsco)

May 13, 2024

Search modes - Find all my search terms

| **#** | **Query** | **Results** |
| --- | --- | --- |
| S1 | (MH "Chronic Disease") OR (MH "Critical Illness") OR (MH "Children with Disabilities") | 102,350 |
| S2 | (MH "Anemia+") OR (MH "Anemia, Sickle Cell") OR (MH "Arthritis") OR (MH "Arthritis, Juvenile Rheumatoid") OR (MH "Asthma") OR (MH "Asthma, Exercise-Induced") OR (MH "Asperger Syndrome") OR (MH "Autistic Disorder") OR (MH "Brain Injuries") OR (MH "Brain Damage, Chronic") OR (MH "Cerebral Palsy") OR (MH "Abnormalities") OR (MH "Cystic Fibrosis") OR (MH "Developmental Disabilities") OR (MH "Diabetes Mellitus") OR (MH "Diabetes Mellitus, Type 1") | 280,806 |
| S3 | (MH "Down Syndrome") OR (MH "Epilepsy+") OR (MH "Heart Diseases") OR (MH "Heart Defects, Congenital") OR (MH "Hereditary Diseases") OR (MH "Hemophilia") OR (MH "Hematologic Diseases") OR (MH "Human Immunodeficiency Virus") OR (MH "HIV-1") OR (MH "HIV Infections") OR (MH "Intellectual Disability") | 178,054 |
| S4 | (MH "Kidney Diseases+") OR (MH "Spina Bifida") OR (MH "Musculoskeletal Diseases+") OR (MH "Child Development Disorders, Pervasive") OR (MH "Celiac Disease") OR (MH "Inflammatory Bowel Diseases") OR (MH "Colitis, Ulcerative") OR (MH "Crohn Disease") OR (MH "Complex Regional Pain Syndromes") OR (MH "Chromosome Aberrations+") OR (MH "Craniofacial Abnormalities") OR (MH "Cleft Palate") OR (MH "Bronchopulmonary Dysplasia") OR (MH "Scoliosis") OR (MH "Scoliosis, Idiopathic, Adolescent") OR (MH "Skin Diseases+") OR (MH "Eczema") OR (MH "Sleep Apnea Syndromes") OR (MH "Sleep Apnea, Central") OR (MH "Sleep Apnea, Obstructive") OR (MH "Hydrocephalus") | 602,297 |
| S5 | TI ( ((chronic* or critical or longterm or "long-term") N4 (disease* or disorder* or illness* or condition* or pain or syndrome*)) ) OR AB ( ((chronic* or critical or longterm or "long-term") N4 (disease* or disorder* or illness* or condition* or pain or syndrome*)) ) | 202,137 |
| S6 | TI ( (“life-limiting” N2 (disease* or disorder* or illness* or condition* or disabilit*)) ) OR AB ( (“life-limiting” N2 (disease* or disorder* or illness* or condition* or disabilit*)) ) | 1,720 |
| S7 | TI ( ((“special health care” or “special healthcare”) N3 (need* or condition* or disease* or disorder* or illness*)) ) OR AB ( ((“special health care” or “special healthcare”) N3 (need* or condition* or disease* or disorder* or illness*)) ) | 1,675 |
| S8 | TI “Medically at-risk" OR AB “Medically at-risk" | 127 |
| S9 | TI ( (complex N4 (“chronic condition*” or “health condition*” or “medical condition*” or” care need*” or “medical need*” or “healthcare need*” or “health care need*” or “health need*” or disabilit*)) ) OR AB ( (complex N4 (“chronic condition*” or “health condition*” or “medical condition*” or” care need*” or “medical need*” or “healthcare need*” or “health care need*” or “health need*” or disabilit*)) ) | 3,704 |
| S10 | TI ( (medical* N2 (complex* or fragile*)) ) OR AB ( (medical* N2 (complex* or fragile*)) ) | 3,969 |
| S11 | TI ( (health N2 (complex* or fragile*)) ) OR AB ( (health N2 (complex* or fragile*)) ) | 5,414 |
| S12 | TI (Technolog* N2 dependen*) OR AB (Technolog* N2 dependen*) | 577 |
| S13 | TI ( (ventilator N2 (dependent* or assist*)) ) OR AB ( (ventilator N2 (dependent* or assist*)) ) | 909 |
| S14 | TI (mechanical* N2 assist* N2 ventilat*) OR AB (mechanical* N2 assist* N2 ventilat*) | 182 |
| S15 | TI “mechanically ventilated” OR AB “mechanically ventilated” | 5,498 |
| S16 | TI “medically fragile technology dependent*” OR AB “medically fragile technology dependent*” | 4 |
| S17 | TI polyhandicap* OR AB polyhandicap* | 7 |
| S18 | TI ( (profound N4 (intellectual or disabilit*)) ) OR AB ( (profound N4 (intellectual or disabilit*)) ) | 929 |
| S19 | TI ( ((intellectual* or brain* or cogniti* or mental) N2 (deficit* or delay* or deviation* or disabil* or disabled or disorder* or dysfunction* or handicap* or impair* or retard*)) ) OR AB ( ((intellectual* or brain* or cogniti* or mental) N2 (deficit* or delay* or deviation* or disabil* or disabled or disorder* or dysfunction* or handicap* or impair* or retard*)) ) | 99,868 |
| S20 | TI ( (deformit* or disabled or disabilit* or handicap*) ) OR AB ( (deformit* or disabled or disabilit* or handicap*) ) | 160,545 |
| S21 | TI ( ((mentally or intellectually or physically) N1 challenged) ) OR AB ( ((mentally or intellectually or physically) N1 challenged) ) | 131 |
| S22 | TI ( (Anemia or anaemia or Thalassemia) ) OR AB ( (Anemia or anaemia or Thalassemia) ) | 30,181 |
| S23 | TI ( ((HbS or hemoglobin or haemoglobin or “sickle cell” or sickling) N2 (condition* or disease* or disorder*)) ) OR AB ( ((HbS or hemoglobin or haemoglobin or “sickle cell” or sickling) N2 (condition* or disease* or disorder*)) ) | 5,681 |
| S24 | TI (“sickle cell” N2 anemia*) OR AB (“sickle cell” N2 anemia*) | 1,057 |
| S25 | TI ( (arthriti* or periarthriti* or “peri arthriti*” or polyarthriti* or “poly arthriti*”) ) OR AB ( (arthriti* or periarthriti* or “peri arthriti*” or polyarthriti* or “poly arthriti*”) ) | 52,831 |
| S26 | TI asthma* OR AB asthma* | 42,036 |
| S27 | TI ( (autis* or asperger* or kanner* syndrome*) ) OR AB ( (autis* or asperger* or kanner* syndrome*) ) | 33,218 |
| S28 | TI ( (brain* N3 (injur* or commotio* or damag* or trauma*)) ) OR AB ( (brain* N3 (injur* or commotio* or damag* or trauma*)) ) | 36,725 |
| S29 | TI ( (tbi or tbis or mtbi or concussion*) ) OR AB ( (tbi or tbis or mtbi or concussion*) ) | 17,344 |
| S30 | TI ( (“cerebral palsy” or (diplegia N1 spastic) or “Little* disease”) ) OR AB ( (“cerebral palsy” or (diplegia N1 spastic) or “Little* disease”) ) | 15,004 |
| S31 | TI ( ((brain* or central*) N2 palsy) ) OR AB ( ((brain* or central*) N2 palsy) ) | 167 |
| S32 | TI ( ((brain or central* or cerebral*) N2 (paralys* or paresis or pareses)) ) OR AB ( ((brain or central* or cerebral*) N2 (paralys* or paresis or pareses)) ) | 165 |
| S33 | TI ( ((congenital* or birth) N2 (abnormal* or anomal* or defect* or deform* or malform*)) ) OR AB ( ((congenital* or birth) N2 (abnormal* or anomal* or defect* or deform* or malform*)) ) | 17,042 |
| S34 | TI “cystic fibros*” OR AB “cystic fibros*” | 9,443 |
| S35 | TI ( ((fibrocystic or “fibro-cystic”) N3 pancrea*) ) OR AB ( ((fibrocystic or “fibro-cystic”) N3 pancrea*) ) | 3 |
| S36 | TI mucoviscidos* OR AB mucoviscidos* | 33 |
| S37 | TI ( (development* N2 (delay* or deviat* or disabilit* or disabled or disorder*)) ) OR AB ( (development* N2 (delay* or deviat* or disabilit* or disabled or disorder*)) ) | 21,004 |
| S38 | TI ( ((autoimmune or brittle or “insulin-dependent” or juvenile) N3 diabet*) ) OR AB ( ((autoimmune or brittle or “insulin-dependent” or juvenile) N3 diabet*) ) | 4,649 |
| S39 | TI ( (("Type 1" or "Type I" or ID) N DM) ) OR AB ( (("Type 1" or "Type I" or ID) N DM) ) | 105 |
| S40 | TI ( (IDDM or T1D) ) OR AB ( (IDDM or T1D) ) | 4,745 |
| S41 | TI (Down* N2 syndrome*) OR AB (Down* N2 syndrome*) | 7,288 |
| S42 | TI ( (mongolism* or mongoloid*) ) OR AB ( (mongolism* or mongoloid*) ) | 54 |
| S43 | TI ( ("trisomy 21" or "trisomy G1" or "trisomy (G)1" or "trisomy G-1" or "trisomy GM" or "trisomy G" or "21 trisomy" or "G1 trisomy" or "G(1) trisomy" or "G-1 trisomy" or "GM trisomy" or "G trisomy") ) OR AB ( ("trisomy 21" or "trisomy G1" or "trisomy (G)1" or "trisomy G-1" or "trisomy GM" or "trisomy G" or "21 trisomy" or "G1 trisomy" or "G(1) trisomy" or "G-1 trisomy" or "GM trisomy" or "G trisomy") ) | 1,264 |
| S44 | TI ( ((chromosom* N1 triplicat*) or trisom*) ) OR AB ( ((chromosom* N1 triplicat*) or trisom*) ) | 2,605 |
| S45 | TI (translocat* N2 DS) OR AB (translocat* N2 DS) | 1 |
| S46 | TI ( “(petit mal” or “grand mal” or “absence status”) ) OR AB ( “(petit mal” or “grand mal” or “absence status”) ) | 173 |
| S47 | TI ( (epileps* or epilept* or seizure* or convulsi*) ) OR AB ( (epileps* or epilept* or seizure* or convulsi*) ) | 39,580 |
| S48 | TI ( ((Dravet* or “Landau-Kleffner*” or “Lennox Gastaut*” or Doose* or Ohtahara* or “Sturge-Weber*” or West or "West's") N2 syndrome*) ) OR AB ( ((Dravet* or “Landau-Kleffner*” or “Lennox Gastaut*” or Doose* or Ohtahara* or “Sturge-Weber*” or West or "West's") N2 syndrome*) ) | 1,143 |
| S49 | TI ( ((sturge* or weber*) N2 (disease* or syndrome*)) ) OR AB ( ((sturge* or weber*) N2 (disease* or syndrome*)) ) | 474 |
| S50 | TI ( (“myoclonic encephalopath*” or “action myoclonus-renal failure syndrome*” or “atypical inclusion-body disease*” or “biotin-responsive encephalopath*” or “haw river syndrome*” or “may white syndrome*” or “myoclonus-nephropathy syndrome*” or “naito oyanagi disease*”) ) OR AB ( (“myoclonic encephalopath*” or “action myoclonus-renal failure syndrome*” or “atypical inclusion-body disease*” or “biotin-responsive encephalopath*” or “haw river syndrome*” or “may white syndrome*” or “myoclonus-nephropathy syndrome*” or “naito oyanagi disease*”) ) | 40 |
| S51 | TI SMEI OR AB SMEI | 27 |
| S52 | TI ( (MERRF or “fukuhara disease*” or “fukuhara disorder*” or “myoencephalopathy ragged-red fiber disease*” or “myoencephalopathy ragged-red fibre disease*”) ) OR AB ( (MERRF or “fukuhara disease*” or “fukuhara disorder*” or “myoencephalopathy ragged-red fiber disease*” or “myoencephalopathy ragged-red fibre disease*”) ) | 35 |
| S53 | TI Lafora OR AB Lafora | 83 |
| S54 | TI ( ((Unverricht* N1 Lundborg) or “Baltic Myoclonus” or “Unverricht disease*” or “Unverricht* syndrome*”) ) OR AB ( ((Unverricht* N1 Lundborg) or “Baltic Myoclonus” or “Unverricht disease*” or “Unverricht* syndrome*”) ) | 25 |
| S55 | TI ( ((infantile or nodding) N2 spasm*) ) OR AB ( ((infantile or nodding) N2 spasm*) ) | 452 |
| S56 | TI hypsarrhythmi* OR AB hypsarrhythmi* | 115 |
| S57 | TI ( ((cardiac* or cardio* or heart*) N2 (abnormalit* or anomal* or atypical* or a-typical* or defect* or deficien* or deform* or disorder* or impair* or malform*)) ) OR AB ( ((cardiac* or cardio* or heart*) N2 (abnormalit* or anomal* or atypical* or a-typical* or defect* or deficien* or deform* or disorder* or impair* or malform*)) ) | 15,359 |
| S58 | TI (tetralog* N2 fallot*) OR AB (tetralog* N2 fallot*) | 1,932 |
| S59 | TI ( ((cardiac* or cardio* or heart*) N5 (congenital* or inborn* or hereditar* or inherit*)) ) OR AB ( ((cardiac* or cardio* or heart*) N5 (congenital* or inborn* or hereditar* or inherit*)) ) | 13,704 |
| S60 | TI ( ((genetic or hereditary or inherited or inborn) N2 (condition* or disease* or disorder*)) ) OR AB ( ((genetic or hereditary or inherited or inborn) N2 (condition* or disease* or disorder*)) ) | 16,177 |
| S61 | TI “single gene defect*” OR AB “single gene defect*” | 76 |
| S62 | TI ( (haemophili* or hemophili* or (("factor VIII" or "factor 8") N3 deficien*)) ) OR AB ( (haemophili* or hemophili* or (("factor VIII" or "factor 8") N3 deficien*)) ) | 2,604 |
| S63 | TI ( ((blood or haematologic* or hematologic*) N2 (condition* or disease* or disorder*)) ) OR AB ( ((blood or haematologic* or hematologic*) N2 (condition* or disease* or disorder*)) ) | 6,508 |
| S64 | TI ( (“HIV-1” or HIV1 or “HIV-I” or HIVI or (HIV N1 “type 1”) or (HIV N1 “type I”)) ) OR AB ( (“HIV-1” or HIV1 or “HIV-I” or HIVI or (HIV N1 “type 1”) or (HIV N1 “type I”)) ) | 6,773 |
| S65 | TI ( (“HIV-2” or HIV2 or “HIV-II” or HIVII or (HIV N1 “type 2”) or (HIV N1 “type II”)) ) OR AB ( (“HIV-2” or HIV2 or “HIV-II” or HIVII or (HIV N1 “type 2”) or (HIV N1 “type II”)) ) | 328 |
| S66 | TI (“immunodeficiency virus*” N2 human*) OR AB (“immunodeficiency virus*” N2 human*) | 17,708 |
| S67 | TI (“immuno-deficiency virus*” N2 human*) OR AB (“immuno-deficiency virus*” N2 human*) | 44 |
| S68 | TI ( ((“acquired immunodeficiency” or “acquired immuno-deficiency") N2 virus*) ) OR AB ( ((“acquired immunodeficiency” or “acquired immuno-deficiency") N2 virus*) ) | 592 |
| S69 | TI “AIDS virus*” OR AB “AIDS virus*” | 106 |
| S70 | TI ( ((kidney* or renal) N2 (condition* or disease* or disorder*)) ) OR AB ( ((kidney* or renal) N2 (condition* or disease* or disorder*)) ) | 46,278 |
| S71 | TI ( (meningomyelocele or “meningo-myelocele” or myelocele) ) OR AB ( (meningomyelocele or “meningo-myelocele” or myelocele) ) | 152 |
| S72 | TI ( ((musculoskelet* or “musculo-skelet*” or muscle* or muscular or skelet* or orthopedic* or orthopaedic*) N2 (abnormalit* or deformit* or disorder* or disease*)) ) OR AB ( ((musculoskelet* or “musculo-skelet*” or muscle* or muscular or skelet* or orthopedic* or orthopaedic*) N2 (abnormalit* or deformit* or disorder* or disease*)) ) | 13,033 |
| S73 | TI ( (MSD or MSDs or MSKD or MSKDs) ) OR AB ( (MSD or MSDs or MSKD or MSKDs) ) | 1,522 |
| S74 | TI ( ((spina* or status) N (bifida* or dysraphi*)) ) OR AB ( ((spina* or status) N (bifida* or dysraphi*)) ) | 203 |
| S75 | TI ( (((cleft or open) N spine*) or rachischis* or schistorrhach*) ) OR AB ( (((cleft or open) N spine*) or rachischis* or schistorrhach*) ) | 169 |
| S76 | TI ( (bowel* N3 inflammatory N3 (condition* or disease* or illness*)) ) OR AB ( (bowel* N3 inflammatory N3 (condition* or disease* or illness*)) ) | 12,139 |
| S77 | TI ( (celiac or coeliac) ) OR AB ( (celiac or coeliac) ) | 6,579 |
| S78 | TI Pain | 137,240 |
| S79 | TI ( (chromosomal N2 (anomal* or abberation* or abnormalit*)) ) OR AB ( (chromosomal N2 (anomal* or abberation* or abnormalit*)) ) | 2,209 |
| S80 | TI ( ((“cranio facial” or craniofacial) N2 (abnormalit* or anomal*)) ) OR AB ( ((“cranio facial” or craniofacial) N2 (abnormalit* or anomal*)) ) | 640 |
| S81 | TI ( ((cleft or lip) N2 palate) ) OR AB ( ((cleft or lip) N2 palate) ) | 4,647 |
| S82 | TI “Bronchopulmonary Dysplasia” OR AB “Bronchopulmonary Dysplasia” | 3,026 |
| S83 | TI scoliosis OR AB scoliosis | 9,356 |
| S84 | TI ( (skin N2 (disease* or disorder*)) ) OR AB ( (skin N2 (disease* or disorder*)) ) | 6,093 |
| S85 | TI eczema OR AB eczema | 3,372 |
| S86 | TI ( (sleep N2 (apnea or disorder*)) ) OR AB ( (sleep N2 (apnea or disorder*)) ) | 21,876 |
| S87 | TI hydrocephal* OR AB hydrocephal* | 3,503 |
| S88 | S1 OR S2 OR S3 OR S4 OR S5 OR S6 OR S7 OR S8 OR S9 OR S10 OR S11 OR S12 OR S13 OR S14 OR S15 OR S16 OR S17 OR S18 OR S19 OR S20 OR S21 OR S22 OR S23 OR S24 OR S25 OR S26 OR S27 OR S28 OR S29 OR S30 OR S31 OR S32 OR S33 OR S34 OR S35 OR S36 OR S37 OR S38 OR S39 OR S40 OR S41 OR S42 OR S43 OR S44 OR S45 OR S46 OR S47 OR S48 OR S49 OR S50 OR S51 OR S52 OR S53 OR S54 OR S55 OR S56 OR S57 OR S58 OR S59 OR S60 OR S61 OR S62 OR S63 OR S64 OR S65 OR S66 OR S67 OR S68 OR S69 OR S70 OR S71 OR S72 OR S73 OR S74 OR S75 OR S76 OR S77 OR S78 OR S79 OR S80 OR S81 OR S82 OR S83 OR S84 OR S85 OR S86 OR S87 | 1,600,184 |
| S89 | (MH "Support, Social") OR (MH "Family Support") OR (MH "Support, Psychosocial") | 107,307 |
| S90 | (MH "Psychotherapy") OR (MH "Art Therapy") OR (MH "Dance Therapy") OR (MH "Dignity Therapy") OR (MH "Guided Imagery") OR (MH "Music Therapy") OR (MH "Pet Therapy") OR (MH "Play Therapy") OR (MH "Psychosocial Intervention") OR (MH "Psychotherapy, Brief") OR (MH "Reality Therapy") OR (MH "Psychotherapy, Psychodynamic") OR (MH "Reminiscence Therapy") OR (MH "Behavior Therapy") OR (MH "Cognitive Therapy") OR (MH "Acceptance and Commitment Therapy") OR (MH "Mindfulness") OR (MH "Dialectical Behavior Therapy") OR (MH "Interpersonal Psychotherapy") | 84,677 |
| S91 | (MH "Alternative Therapies") OR (MH "Aromatherapy") OR (MH "Mind Body Techniques") OR (MH "Hypnosis") OR (MH "Meditation") OR (MH "Relaxation Techniques") OR (MH "Tai Chi") OR (MH "Yoga") | 65,195 |
| S92 | (MH "Creative Arts Therapy") OR (MH "Psychodrama") OR (MH "Recreational Therapy") OR (MH "Adaptation, Psychological") OR (MH "Coping") OR (MH "Family Coping") OR (MH "Self-Efficacy") OR (MH "Counseling") OR (MH "Anticipatory Guidance") OR (MH "Motivational Interviewing") OR (MH "Quality of Life/PF/PC") OR (MH "Stress, Psychological/PC/PF") OR (MH "Anxiety/PF/PC") OR (MH "Depression/PC/PF") OR (MH "Spirituality") OR (MH "Spiritual Care") OR (MH "Financial Stress") OR (MH "Stress, Occupational") OR (MH "Information Seeking Behavior") OR (MH "Help Seeking Behavior") | 233,992 |
| S93 | TI ( (social N3 (support* or network* or system*)) ) OR AB ( (social N3 (support* or network* or system*)) ) | 60,904 |
| S94 | TI ( (support N3 (group* or system* or emotion* or physical)) ) OR AB ( (support N3 (group* or system* or emotion* or physical)) ) | 39,991 |
| S95 | TI ( ((psychosocial or “psycho-social” or biopsychosocial) N3 (support* or intervention* or program* or care or outcome* or therap* or distress or problem* or need* or adjust* or wellbeing or “well-being” or wellness or burden* or factor*)) ) OR AB ( ((psychosocial or “psycho-social” or biopsychosocial) N3 (support* or intervention* or program* or care or outcome* or therap* or distress or problem* or need* or adjust* or wellbeing or “well-being” or wellness or burden* or factor*)) ) | 35,718 |
| S96 | TI ( ((psycho* or behavior* or behaviour* or education* or group* or cogniti* or family) N3 therapy) ) OR AB ( ((psycho* or behavior* or behaviour* or education* or group* or cogniti* or family) N3 therapy) ) | 43,763 |
| S97 | TI ( ((cope* or coping) N3 (skill* or strateg* or intervention* or program* or behavio*)) ) OR AB ( ((cope* or coping) N3 (skill* or strateg* or intervention* or program* or behavio*)) ) | 20,197 |
| S98 | TI ( ((psychologic* or emotion* or stress or distress or anxiety or depress*) N3 (adjust* or adapt* or reduce or reduction or support* or “well-being” or wellbeing or wellness or burden* or factor*)) ) OR AB ( ((psychologic* or emotion* or stress or distress or anxiety or depress*) N3 (adjust* or adapt* or reduce or reduction or support* or “well-being” or wellbeing or wellness or burden* or factor*)) ) | 100,924 |
| S99 | TI “self-efficacy” OR AB “self-efficacy” | 29,318 |
| S100 | TI ( (“mental health” N3 (need* or care or support* or intervention* or program*)) ) OR AB ( (“mental health” N3 (need* or care or support* or intervention* or program*)) ) | 32,183 |
| S101 | TI ( (spiritual* or pastoral or religio*) ) OR AB ( (spiritual* or pastoral or religio*) ) | 42,159 |
| S102 | TI ( ((financial or economic or occupation* or work or employ* or job*) N3 (stress* or toxicity or burden* or insecurit*)) ) OR AB ( ((financial or economic or occupation* or work or employ* or job*) N3 (stress* or toxicity or burden* or insecurit*)) ) | 24,792 |
| S103 | TI ( (information N3 (need* or requirement* or health or request* or seek*)) ) OR AB ( (information N3 (need* or requirement* or health or request* or seek*)) ) | 49,888 |
| S104 | S89 OR S90 OR S91 OR S92 OR S93 OR S94 OR S95 OR S96 OR S97 OR S98 OR S99 OR S100 OR S101 OR S102 OR S103 | 684,700 |
| S105 | (MH "Patients") OR (MH "Adolescent, Hospitalized") OR (MH "Child, Hospitalized") OR (MH "Critically Ill Patients") OR (MH "Infant, Hospitalized") OR (MH "Inpatients") | 116,782 |
| S106 | (MH "Hospitals") OR (MH "Hospitals, Pediatric") OR (MH "Hospital Units") OR (MH "Nurseries, Hospital") OR (MH "Intensive Care Units, Pediatric") OR (MH "Intensive Care Units, Neonatal") OR (MH "Patient Admission") OR (MH "Hospitalization") | 175,126 |
| S107 | (MH "Patient Admission") OR (MH "Patient Discharge") OR (MH "Early Patient Discharge") OR (MH "Transfer, Discharge") OR (MH "Readmission") OR (MH "Continuity of Patient Care") OR (MH "Hospital to Home Transition") OR (MH "Transitional Care") | 81,621 |
| S108 | TI ( ((pediatric* or paediatric*) N3 (room* or department* or facilit*)) ) OR AB ( ((pediatric* or paediatric*) N3 (room* or department* or facilit*)) ) | 7,932 |
| S109 | TI ( (ward* or wards or unit or units) ) OR AB ( (ward* or wards or unit or units) ) | 210,720 |
| S110 | TI ( (PICU* or ICU or ICUS or NICU* or “intensive care” or “critical care” or hospital* or inpatient*) ) OR AB ( (PICU* or ICU or ICUS or NICU* or “intensive care” or “critical care” or hospital* or inpatient*) ) | 663,756 |
| S111 | TI ( (discharg* or postdischarg* or “post-discharg*” or readmission or “re-admission” or readmit* or “re-admit*”) ) OR AB ( (discharg* or postdischarg* or “post-discharg*” or readmission or “re-admission” or readmit* or “re-admit*”) ) | 112,043 |
| S112 | TI (transition* N3 home) OR AB (transition* N3 home) | 1,839 |
| S113 | TI (hospital N3 home) OR AB (hospital N3 home) | 8,333 |
| S114 | TI ( ((transfer* or coordinat* or transitional) N3 care) ) OR AB ( ((transfer* or coordinat* or transitional) N3 care) ) | 16,538 |
| S115 | TI ( (home N3 (care or healthcare)) ) OR AB ( (home N3 (care or healthcare)) ) | 38,975 |
| S116 | S105 OR S106 OR S107 OR S108 OR S109 OR S110 OR S111 OR S112 OR S113 OR S114 OR S115 | 946,422 |
| S117 | (MH "Child") OR (MH "Child, Preschool") OR (MH "Infant") OR (MH "Infant, Newborn") OR (MH "Adolescence") | 1,115,997 |
| S118 | TI ( (pediatric* or paediatric* or child*) ) OR AB ( (pediatric* or paediatric* or child*) ) | 677,158 |
| S119 | TI ( (boy or boys or girl* or juvenile* or teen* or tween* or preteen* or “pre-teen*” or youth* or adolesc* or prepubesc* or pubescen*or “pre-pubesc*”) ) OR AB ( (boy or boys or girl* or juvenile* or teen* or tween* or preteen* or “pre-teen*” or youth* or adolesc* or prepubesc* or pubescen*or “pre-pubesc*”) ) | 288,317 |
| S120 | TI ( (preschool* or “pre-school*” or “school-age*” or toddler* or infant* or baby or babies or newborn* or neonate*) ) OR AB ( (preschool* or “pre-school*” or “school-age*” or toddler* or infant* or baby or babies or newborn* or neonate*) ) | 223,777 |
| S121 | S117 OR S118 OR S119 OR S120 | 1,441,575 |
| S122 | (MH "Psychosocial Intervention") | 1,330 |
| S123 | (MH "Program Development") OR (MH "Program Evaluation") OR (MH "Program Implementation") OR (MH "Program Planning") OR (MH "Health Education") | 135,744 |
| S124 | TI ( (intervention* or program* or training or course* or education or project* or curriculum or model* or framework*) ) OR AB ( (intervention* or program* or training or course* or education or project* or curriculum or model* or framework*) ) | 1,900,860 |
| S125 | S122 OR S123 OR S124 | 1,942,988 |
| S126 | S88 AND S104 AND S116 AND S121 AND S125 | 4,442 |
| S127 | S88 AND S104 AND S116 AND S121 AND S125 | 4,199 |
